# Accumulation of copy number alterations and clinical progression across advanced prostate cancer

**DOI:** 10.1186/s13073-022-01080-4

**Published:** 2022-09-05

**Authors:** Emily Grist, Stefanie Friedrich, Christopher Brawley, Larissa Mendes, Marina Parry, Adnan Ali, Aine Haran, Alex Hoyle, Claire Gilson, Sharanpreet Lall, Leila Zakka, Carla Bautista, Alex Landless, Karolina Nowakowska, Anna Wingate, Daniel Wetterskog, A. M. Mahedi Hasan, Nafisah B. Akato, Malissa Richmond, Sofeya Ishaq, Nik Matthews, Anis A. Hamid, Christopher J. Sweeney, Matthew R. Sydes, Daniel M. Berney, Stefano Lise, STAMPEDE investigators, Mahesh K. B. Parmar, Noel W. Clarke, Nicholas D. James, Paolo Cremaschi, Louise C. Brown, Gerhardt Attard

**Affiliations:** 1 Cancer Institute, University College London, London, UK; 2 MRC Clinical Trials Unit at University College London, London, UK; 3 GU Cancer Research/FASTMAN Group, Manchester Cancer Institute, Manchester, UK; 4 The Christie and Salford Royal NHS Foundation Trusts, Manchester, UK; 5 The Institute of Cancer Research, London, UK; 6 Imperial College, London, UK; 7 Department of Medical Oncology, Dana-Farber Cancer Institute, Boston, MA USA; 8 Barts Cancer Institute, Queen Mary University of London, London, UK; 9 The Royal Marsden Hospital NHS Foundation Trust and The Institute of Cancer Research, London, UK

**Keywords:** Advanced prostate cancer, Genomic biomarkers, Copy number alteration, STAMPEDE trial

## Abstract

**Background:**

Genomic copy number alterations commonly occur in prostate cancer and are one measure of genomic instability. The clinical implication of copy number change in advanced prostate cancer, which defines a wide spectrum of disease from high-risk localised to metastatic, is unknown.

**Methods:**

We performed copy number profiling on 688 tumour regions from 300 patients, who presented with advanced prostate cancer prior to the start of long-term androgen deprivation therapy (ADT), in the control arm of the prospective randomised STAMPEDE trial. Patients were categorised into metastatic states as follows; high-risk non-metastatic with or without local lymph node involvement, or metastatic low/high volume. We followed up patients for a median of 7 years. Univariable and multivariable Cox survival models were fitted to estimate the association between the burden of copy number alteration as a continuous variable and the hazard of death or disease progression.

**Results:**

The burden of copy number alterations positively associated with radiologically evident distant metastases at diagnosis (*P*=0.00006) and showed a non-linear relationship with clinical outcome on univariable and multivariable analysis, characterised by a sharp increase in the relative risk of progression (*P*=0.003) and death (*P*=0.045) for each unit increase, stabilising into more modest increases with higher copy number burdens. This association between copy number burden and outcome was similar in each metastatic state. Copy number loss occurred significantly more frequently than gain at the lowest copy number burden quartile (*q*=4.1 × 10^−6^). Loss of segments in chromosome 5q21-22 and gains at 8q21-24, respectively including *CHD1* and *cMYC* occurred more frequently in cases with higher copy number alteration (for either region: Kolmogorov–Smirnov distance, 0.5; adjusted *P*<0.0001). Copy number alterations showed variability across tumour regions in the same prostate. This variance associated with increased risk of distant metastases (Kruskal-Wallis test *P*=0.037).

**Conclusions:**

Copy number alteration in advanced prostate cancer associates with increased risk of metastases at diagnosis. Accumulation of a limited number of copy number alterations associates with most of the increased risk of disease progression and death. The increased likelihood of involvement of specific segments in high copy number alteration burden cancers may suggest an order underlying the accumulation of copy number changes.

**Trial registration:**

ClinicalTrials.gov NCT00268476, registered on December 22, 2005. EudraCT 2004-000193-31, registered on October 4, 2004.

**Supplementary Information:**

The online version contains supplementary material available at 10.1186/s13073-022-01080-4.

## Background

Copy number alteration is common in malignancy and can define biologically relevant sub-groups with distinct outcomes [1–5]. Genomic copy number alterations often involve segments of DNA extending for thousands of bases, harbouring several putative cancer drivers. They can result from underlying chromosomal instability that in many cancers is associated with worse outcomes [4–6]. Prostate cancer is characterised by a well-described repertoire of copy number alterations that commonly include loss of regions involving tumour suppressor genes *NKX3.1*, *TP53*, *PTEN* and *RB1* [7–9]. In low- and intermediate-risk prostate cancer, increasing burden of copy number alteration is associated with shorter time to biochemical recurrence and death from prostate cancer [4, 5, 10].

We aimed to interrogate non-focal copy number alterations in tumour samples from patients presenting with advanced prostate cancer and linked to prospectively collected clinical outcome. We generated pan-genome copy number profiles from low coverage whole genome sequencing data on tumour samples obtained prior to the start of long-term androgen deprivation therapy (ADT) from 300 advanced prostate cancer patients, followed up for survival and disease progression, in the control arm of the Systemic Therapy in Advancing or Metastatic Prostate cancer: Evaluation of Drug Efficacy (STAMPEDE, MRC-PR08, NCT00268476) trial. This is a multi-stage platform trial that since 2005 has randomised men to a control arm of standard-of-care, including ADT, or one of seven previously reported and three as yet unreported contemporaneously recruited experimental comparisons [11–16]. While intense clinical trial evaluation has been performed in this disease setting, we have a limited understanding of the molecular underpinnings of clinical progression in this population.

## Methods

### Trial design and population

The patient cohort with advanced prostate cancer was recruited to the STAMPEDE trial which was prospectively registered as follows: https://clinicaltrials.gov/ct2/show/NCT00268476, https://www.isrctn.com/ISRCTN78818544.

The STAMPEDE patient cohort has been described previously [11, 12]. Briefly, prostate adenocarcinoma patients were eligible if they had either localised high-risk disease that was node-positive (M0N1) or if node-negative (M0N0), had at least two of tumour stage category T3/4, prostate-specific antigen (PSA) ≥40 ng/ml, or Gleason sum score 8–10; or extra-pelvic metastatic disease (M1) confirmed on conventional whole-body computed tomography (CT) and technetium bone scans. A full list of inclusion and exclusion criteria for the STAMPEDE trial is available in Additional file 3: supplemental methods. Pre-ADT PSA was obtained up to 6 months before randomisation. Grading group, age, stage, performance status and metastatic status, namely M0N0, M0N1 or M1, were recorded by clinical sites and accessed from the STAMPEDE trial database. Metastatic status was further classified into low and high volume based on central imaging review (CT and whole-body technetium bone scans), using the CHAARTED criteria; high volume is defined as the presence of visceral metastases or greater than or equal to 4 bone metastases with 1 or more beyond the vertebral bodies and pelvis [17].

STAMPEDE patients were eligible to be randomly selected for inclusion within this biomarker study if the following criteria were met; consent obtained for use of tumour tissue in additional research, randomised to the control arm of STAMPEDE at UK trial sites before the addition of docetaxel or AR-targeted therapies was allowed (Additional file 2: Table S1), diagnostic formalin-fixed, paraffin-embedded (FFPE) prostate biopsies available for analyses and if more than 10 ng of DNA could be extracted from at least one tumour-enriched region from a diagnostic core biopsy.

In a hypothesis-driven exploratory analyses to determine the association between copy number alteration and clinical outcome, we aimed to generate copy number profiles for 300 STAMPEDE cases (CN-300 cohort) randomly selected from patients eligible for these analyses (Additional file 1: Fig. S1)). All comparator arms within the STAMPEDE trial randomising patients to control arm treatment, prior to the addition of docetaxel or AR-targeted therapies to ADT, have now closed. The first patient was randomised to the control arm of STAMPEDE on 15 November 2005 and the last patient was randomised on 16 December 2015 prior to the addition of docetaxel or AR-targeted therapies to ADT. The first patient randomly selected for inclusion in the CN-300 cohort was randomised to the control arm of the STAMPEDE trial on 14 September 2006 and the last patient was randomised on 12 November 2015. The CN-300 cohort was followed up for a median of 7 years (range 6.8–8 years). All analyses linking molecular to clinical data were performed by the Medical Research Council Clinical Trials Unit (MRC CTU) statisticians (CB) who had sole access to clinical data.

Diagnostic formalin-fixed, paraffin-embedded tissue blocks were retrieved from STAMPEDE trial sites by the MRC CTU and centralised in the Wales Cancer Biobank (WCB), where all identifying details were removed, and samples and reports were labelled with the individual’s STAMPEDE trial number. Tumour samples were transferred to lab-based researchers who had no access to clinical data other than pseudo-anonymised local pathology reports.

### Data generation and analysis

Fresh haematoxylin and eosin slides from every tumour block were centrally assessed by two genitourinary pathologists (LM and DB) for tumour cellularity and scored using contemporary Gleason score and corresponding grade groups (ISUP2014/ WHO2016) [18, 19], referred to as central grade group. Local grade group was derived from the Gleason score recorded in the trial patient report forms by sites using local pathology reports. For every patient, an ‘index core’ was selected defined as the core harbouring the tumour-enriched area of the highest Gleason grade and tumour cellularity.

Blocks with sufficient tumour were cut at 10 microns, deparaffinised and stained with nuclear fast red. The tumour-enriched region from each core on each section was micro-dissected separately under a stereoscope to maximise tumour purity. Tissue from individual diagnostic cores was digested with proteinase K overnight and extracted using a column-based isolation method (Quick-DNA FFPE miniprep kit, Zymo) and quantified using Qubit (Invitrogen).

DNA was fragmented using Covaris E220 (Agilent), repaired using the NEBNext FFPE repair kit (NEB) and libraries were generated using the NEBNext Ultra II DNA library generation preparation kit (NEB) as per the manufacturer’s instructions. Equimolar pools were sequenced using the Illumina Novaseq on 50bp paired-end runs.

Genomes were aligned to the human genome assembly GRCh37 (hg19), and the Quantitative DNA Sequencing (QDNAseq R package, v3.12 [20]) package optimised for FFPE samples was applied as a depth of coverage method to determine copy number alterations [21]. Copy number calls and segmentation of 500-kb bins were performed using the CGHcall package. Segments are defined as merged neighbouring bins with similar copy number alteration. Copy number burden was defined as the number of genome segments classified as showing gain or loss compared to the median read count across the genome, divided by the total number of segments within the autosome. Given our read depth approach on low coverage samples, we report relative deviations in read count to the median read count across the sample and we are not able to report absolute copy number alterations. Per-segment residuals were estimated as the distance of each bin from the related segment; the standard deviation of the segment residuals was derived as a post-processing per-sample quality control measure (mean segment standard deviation).

STAMPEDE clinical trial outcome measures were defined in the STAMPEDE protocol and measured from time of randomisation to event. Patients without an event were censored at the most recent event-free clinical evaluation. The STAMPEDE clinical trial outcome measures of relevance to this ancillary biomarker study are as follows: primary outcome measure was overall survival (OS). Secondary outcome measures were (1) prostate cancer-specific survival (PCSS), reviewed centrally for cause; (2) failure-free survival (FFS) that included any of biochemical failure, local or distant radiographic progression or death from prostate cancer; (3) progression-free survival (PFS), failure-free survival but excluding biochemical failure; (4) metastatic progression-free survival (MPFS) defined as new distant metastases or death from prostate cancer. Clinical follow-up data for this analysis were frozen on 3 February 2021.

### Statistics

The sample size of 300 was not determined through power calculations. Unless otherwise specified, all hypothesis tests required evidence at the 5% significance level to consider rejecting the null hypothesis. All statistical tests were performed using R version 3.6.1 and STATA version 16.1. Student’s *t* tests and chi-squared tests were used to compare the baseline characteristics of the 300 patients selected for analysis with the 3106 patients in the whole STAMPEDE control arm who had been randomised during the same period. Univariable linear regression models with square root-transformed copy number alteration burden specified as the response variable were used to assess the association between the burden of copy number alterations and clinical variables. Univariable and multivariable Cox survival models were fitted to estimate the association between burden of copy number alteration identified in the index core as a continuous variable and the hazard of each trial outcome. Multivariable models included the following as adjustment variables: (1) grading group; (2) metastatic states (M0N0, M0N1, M1 low and M1 high); (3) pre-ADT serum PSA, log transformed; (4) age at randomisation (years); (5) percentage tumour cellularity. A more flexible functional form was used for burden where this was seen to improve the fit of the model. This was based on established fractional polynomial selection technique searching across the range of potential powers: −2, −1, −0.5, ln(), 0.5, 1, 2, 3, with a more complex specification used when there was evidence at the 10% significance level that this provided a better fit to the data than simpler alternatives. Comparisons of model fit were based on data from all patients to be included in the relevant analysis. Relevant details are presented in Additional file 2: Table S5. To ensure consistency between statistical models for different outcomes and given the limited ability to detect an improved fit with higher order specifications, we only considered first degree—FP(1)—fractional polynomial models. Each figure representing the relative hazard can be used to determine the relative change in hazard associated with a change in copy number burden for patients with the corresponding baseline metastatic state, conditional on grading group, pre-ADT PSA, age at randomisation and tumour cellularity. For example, for two patients with baseline M0N0 disease and identical values for all other clinical factors adjusted for, if one patient has a copy number burden of 10% and the other patient a 2% burden, the first patient is estimated to have a 46% higher hazard of a FFS event than the patient with lower burden. For two patients with baseline low volume metastatic disease, one with 10% copy number burden and the other 2% burden and all other factors identical, the patient with higher burden is estimated to have a 68% higher hazard of FFS event. The relative change in hazard is determined by taking the ratio of the *y*-axis values corresponding to the copy number burden values. Note that this depends on both the absolute difference in *y*-axis value as well as the ‘reference’ value. Post hoc analyses assessed whether there was evidence of differential association between the burden of copy number alteration and the hazard of the outcomes according to baseline metastatic state. An additional multivariable Cox survival model was fitted for each outcome as described above, with interaction terms added to reflect differences according to metastatic state. The functional form used for the burden of copy number alteration was the same as in the main multivariable model for each outcome. A likelihood ratio test was used to assess the evidence of improved model fit compared to the main model. Only the burden of copy number alteration identified in the index core was included in univariable and multivariable models.

## Results

### The STAMPEDE CN-300 cohort

**Fig. 1:**
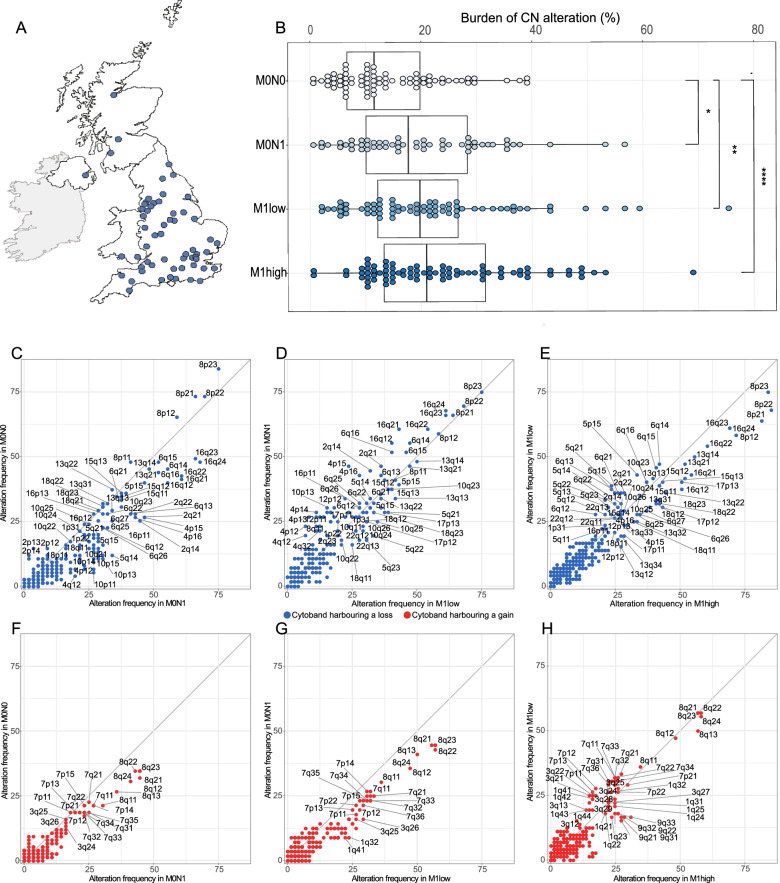
CN-300 cohort and association of the burden of copy number alteration with metastatic states. **A** Map demonstrating all UK STAMPEDE trial sites recruiting patients included in the CN-300 cohort. **B** Distribution of burden of copy number (CN) alteration (%) in tumour-enriched region of index core split by metastatic states (*N*=284; 16 metastatic patients with unknown designation for low versus high volume were excluded). **C–E** Alteration frequency (%) of patients with at least one segment of loss mapped to denoted cytobands in **C** M0N1 versus M0N0; **D** M1 low versus M0N1; **E** M1 high versus M1 low. **F–H** Alteration frequency (%) of patients with at least one segment of gain mapped to denoted cytobands in **F** M0N1 versus M0N0; **G** M1 low versus M0N1; and **H** M1 high versus M1 low

The STAMPEDE trial recruited 3106 patients between 2005 and 2015 to the control arm and after randomly selecting 359 patients iteratively who had diagnostic tissue available, we performed copy number profiling on the first 300 cases successfully sequenced, hereafter referred to as the CN-300 cohort. All cases randomly chosen started long-term ADT in the control arm of the STAMPEDE clinical trial at one of 58 clinical trial sites in the UK. The trial continued to recruit after 2015 but docetaxel and subsequently androgen receptor (AR) targeting agents became standard-of-care, so we elected to have 2015 as the cut-off for patient inclusion in order to have a homogenous population (Additional file 1: Fig. S1, Additional file 2: Table S1 and Fig. 1A). We retrieved diagnostic FFPE biopsies of the prostate and centrally reviewed tumour core cellularity and histological Gleason grade. We excluded patients that had been biopsied after the start of ADT (*N*=3) and had a tumour cellularity of less than 40% or insufficient DNA after extraction (*N*=50). Next-generation sequencing (NGS) libraries failed to meet our quality requirements for six patients (Additional file 1: Fig. S2). Amongst 131 (44%) patients who were non-metastatic by conventional imaging (M0), 56 patients had local lymph node involvement (M0N1) and 75 patients had no local lymph node involvement (M0N0). The 169 (56%) patients with radiologically confirmed metastases were divided into 81 high (M1 high) and 72 low (M1 low) volume based on previously described criteria [17]. We were unable to classify metastases into low or high volume for 16 patients. We observed no significant difference in baseline clinical characteristics between the CN-300 cohort and all 3106 patients assigned to the same standard-of-care group over the same time period (Additional file 1: Table S2). The median histopathologically defined tumour cellularity of the index tumour area from which we extracted DNA was lower in metastatic compared to non-metastatic patients (70% versus 80%, Kruskal-Wallis *P*=0.011; Additional file 1: Fig. S3).

Non-metastatic patients received ADT for at least 2 years and were recommended for curative-intent radiotherapy to the prostate and pelvic lymph nodes if indicated [22, 23] as previously reported [24]. Metastatic patients started life-long ADT and did not receive radiotherapy to the prostate. We confirmed that in the CN-300 cohort, the number of events at 4 years was higher with increasing disease volume (Additional file 1: Fig. S4 and Additional file 2: Table S3). Follow-up was for a median of 7 years (interquartile range 6.8–8 years) (Additional file 2: Table S4).

### Burden and frequency of copy number alteration across metastatic states

Unique to our study is the inclusion of patients with high-risk, non-metastatic or metastatic disease, accrued to the same prospective clinical trial protocol with all samples processed in the same way. We performed whole genome sequencing and through a series of modelling experiments concluded that allowing a minimum of 10 ng DNA input from micro-dissected tumour regions, we could achieve 1-5X pan-genome unique reads (Additional file 1: Fig S5) [21]. We derived the burden of copy number alteration for the histologically defined index core from every patient: the median percentage of genome segments that showed an alteration (Additional file 1: Fig. S6) in the CN-300 cohort was 18%, range, 0.2–75.4%. Prior reports have similarly measured the percentage of genome altered using different assays in low- to intermediate-risk prostate cancers and identified median copy number burdens ~7.5% [5, 10]. Targeted NGS assays in cohorts of metastatic hormone-sensitive prostate cancer patients (including primary and metastatic biopsies) reported a higher median ~32% [25], supporting this as a reliable estimation of copy number burden in aggressive primary tumours at presentation. We found no evidence of a relevant association between putative technical confounders and burden of copy number alteration, including coverage, mean segment standard deviation, DNA input and histopathologically defined tumour cellularity (Additional file 1: Fig S7 and Fig. S8).

Higher burden of copy number alteration identified in the index core per patient was positively associated with the presence of distant metastases (median 13.8% vs 20.9%; *P*=0.00006) and amongst non-metastatic patients, with the presence of pelvic lymph nodes (median M0N0 11.6% vs M0N1 17.7% *P*=0.008) (Fig. 1B). In metastatic patients, there was no detectable significant difference between metastatic low and high volume sub-groups (median: M1 low 19.8% vs M1 high 21.0%, *P*=0.356). Increasing burden of copy number alteration was also associated with grading group (*P*=0.03), although as nearly two-thirds of patients were grading group 5, this observation is less certain (Additional file 1: Fig. S9).

Given the differential outcomes and reports of enrichment of gene aberrations between metastatic states [9, 25, 26], we then plotted the frequency of segmental copy number alterations in each metastatic disease state (Fig. 1C–H). The frequency of segments harbouring deletions was higher in node-positive non-metastatic compared to node-negative but equivalent across higher volume metastatic states; segments with an amplification were more frequent in non-metastatic node-positive compared to node-negative and in metastatic patients compared to non-metastatic.

### Burden of copy number alterations and risk of clinical progression

**Fig. 2:**
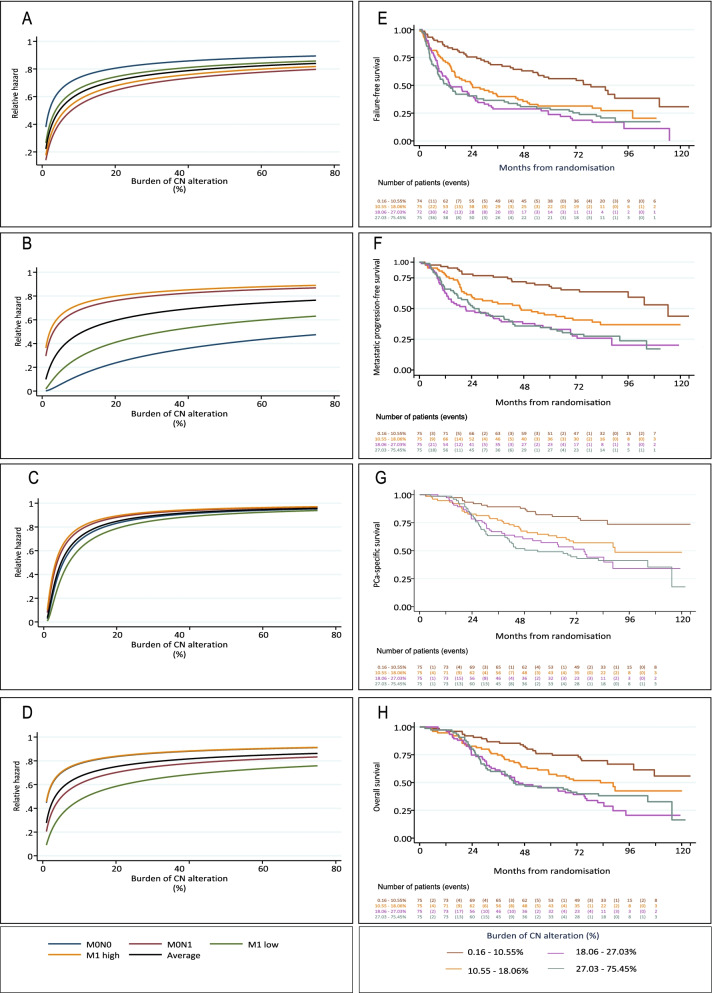
Association of copy number alteration with clinical outcome measures. **A–D** Cox survival model demonstrating adjusted estimate of impact of burden of copy number (CN) alteration as a continuous variable on hazard of **A** failure-free survival; **B** metastatic progression-free survival; **C** prostate cancer-specific survival; **D** overall survival. Variables included in adjusted analysis: (1) grading group; (2) metastatic state (M0N0, M0N1, M1 low and M1 high); (3) Pre-ADT serum PSA log transformed; (4) age at randomisation; (5) tumour cellularity (%). Black line represents the impact of the burden of copy number alteration on relative risk for 284/300 patients for which we could determine disease state (16 M1 patients with unknown designation for low versus high volume were excluded). Each coloured line represents sub-groups of the CN-300 cohort defined by metastatic state. **E–H** Kaplan-Meier estimates. CN-300 cohort split into quartile groups determined by the burden of copy number alteration. Time-to event; **E** failure-free survival; **F** metastatic progression-free survival; **G** prostate cancer-specific survival; **H** overall survival

We next determined the association between the burden of copy number alteration and clinical outcome. We found evidence of a positive, non-linear association between increasing burden of copy number alteration and the hazard of all outcome measures in unadjusted univariable models (failure-free survival *P*=4.6 × 10^−8^, metastatic progression-free survival *P*=5.9 × 10^−10^, prostate cancer-specific survival *P*=4.9 × 10^−9^ and overall survival *P*=3.3 × 10^*−7*^*)* (Additional file 1: Fig S10 and Additional file 2: Table S5). We then performed multivariable analyses adjusting for clinically relevant variables, namely (1) grading group, (2) log PSA prior to starting ADT, (3) age at randomisation (years), (4) metastatic status (M0N0, M0N1, M1 low and M1 high) and (5) tumour cellularity (%). Controlling for these variables, copy number burden was significantly associated with an increased risk of treatment failure (*P*=0.004), metastatic progression (*P=*0.003), death from prostate cancer (*P*=0.01) and death from any cause (*P*=0.045) (Fig. 2A–D and Additional file 2: Table S5). For each outcome, a positive, non-linear relationship was characterised by steep increases in the relative hazard of an event when copy number burden was increased from a low starting point. Increases in relative risk associated with increasing copy number burden were smaller at higher burden levels. We found no evidence to indicate that this association differed across the four metastatic states (interaction *P-*value for risk: of failure, 0.95; metastatic progression, 0.40; death from prostate cancer, 0.95; death from any cause, 0.85; Fig. 2A–D, Additional file 2: Table S6): although the risk of progression and death in a metastatic patient is worse than for a tumour with the same burden of copy number alteration in a non-metastatic patient, the increase in the risk of an event per unit increase in copy number burden is equivalent and diminishes as the burden increases beyond a threshold, which is similar across metastatic states.

To explore the non-linear association, we plotted Kaplan-Meier survival estimates for equal-sized quartiles of the CN-300 cohort (burden of copy number alteration up to 10.55%, 10.55–18.06%, 18.06–27.03%, 27.03–75.45%) and visually confirmed that patients within the lowest burden quartile had better outcomes than all three higher burden quartiles: at 4 years, fewer than 25% in the lowest burden quartile had died from prostate cancer compared to more than 40% in the upper two copy number burden quartiles (Fig. 2E–H and Table 1).

**Table 1:** Survival at 4 years follow-up for burden of copy number alteration quartile sub-groups (Kaplan-Meier estimates)

Clinical trial endpoint	Estimated survival (%)			
Copy number alteration burden (Quartile)	Q1	Q2	Q3	Q4
Failure-free survival	63	36	29	31
Progression-free survival	77	50	40	43
Metastatic progression-free survival	82	57	45	43
Prostate cancer-specific survival	88	67	61	52
Overall survival	83	64	48	48

Legend: range of copy number burdens (%) included in quartiles: (Q1: less than 10.55; Q2: 10.55–18.06; Q3: 18.06–27.03; Q4: 27.03–75.45)

### Frequency of gains compared to losses with increasing copy number burden

**Fig. 3:**
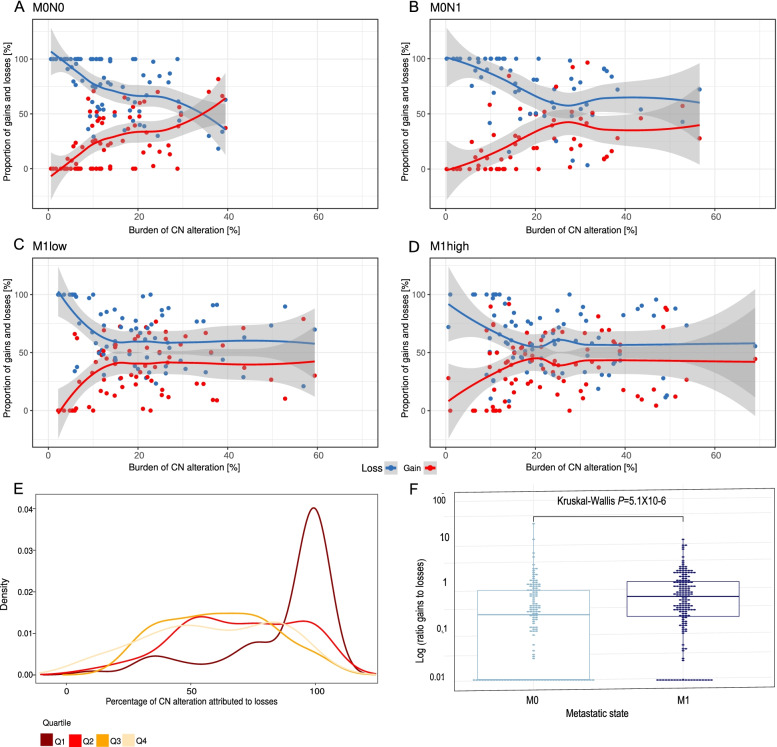
Proportion of copy number alteration attributable to loss as compared to gain. **A–D** Patients are ranked with ascending burden of copy number (CN) alteration (%) identified in the index core on the *x*-axis. *Y*-axis represents the proportion of copy number alteration (%) attributable to a gain (red dot) as compared to a loss (blue dot). The line represents the conditional mean (blue=loss, red=gain), i.e. an estimate of the mean proportions conditional on the number of patients (LOESS function). The grey band indicates the confidence interval (level of confidence 0.95) **A** M0N0 patients; **B** M0N1 patients; **C** M1 low patients; **D** M1 high patients. **E** Density plot representing the proportion of the genome altered by a copy number loss in the index core. Each line sub-groups the CN-300 cohort into burden of copy number alteration quartiles. **F** Boxplot demonstrating the difference in ratio of the burden of copy number alteration due to a gain as compared to a loss (log transformed) between non-metastatic (M0) and metastatic (M1) patients

It has been reported that in carcinogenesis, copy number deletions are earlier events than copy number gains [2, 27]. We evaluated the proportion of copy number alterations that constituted either a loss or gain split by metastatic state (Fig. 3A–D). We observed a consistent trend for more segmental losses at lower copy number burden with gains increasing in frequency with rising copy number burden across all metastatic states (Fig. 3E). We compared the actual ratio of losses to gains with an ‘expected’ ratio that assumed alterations occurred randomly. We confirmed that deletions occurred significantly more frequently in the lowest and second lowest quartiles (Fisher’s exact test corrected with Benjamini-Hochberg method, *q* = 4.1 × 10^−6^ and 0.002 respectively) but not the third or fourth quartile (*q* = 0.1 and 0.3 respectively). We then determined that the ratio of copy number alteration attributable to gains as compared to losses was higher in tumours from patients with metastatic as compared to non-metastatic disease (Kruskal-Wallis *P*=5.1 × 10^−6^, Fig. 3F). We conclude that deletions account for the majority of alterations at low copy number burden, but as copy number alteration increases, gains occur more frequently until they reach similar proportions.

### Frequency distribution of alterations in copy number burden quartiles

**Fig. 4:**
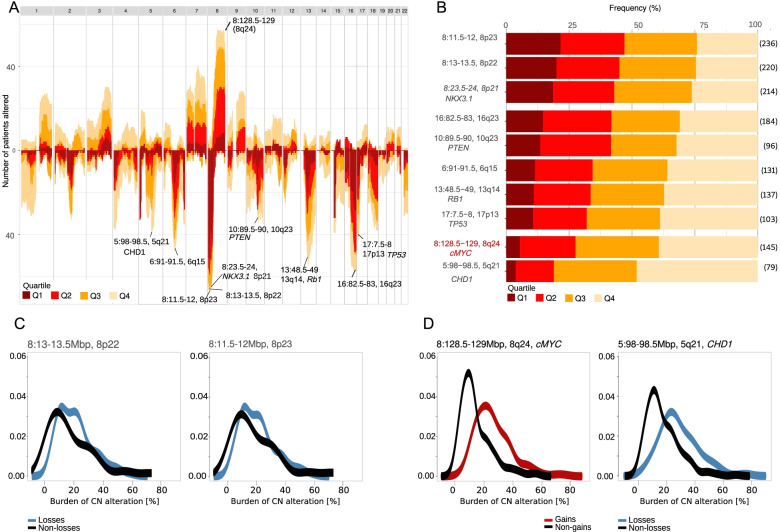
Frequency of copy number alteration. **A** Landscape of copy number alteration across the autosome. The CN-300 cohort is split into quartile groups defined by burden of copy number alteration (%) in index core (red to yellow=quartile1-4). *Y*-axis=Number of patients with an alteration (above midline=copy number gain; below midline=copy number loss). *X*-axis=genomic location. Regions of interest are annotated by chromosome followed by genomic location and mapped cytoband. **B** Stacked bar chart of selected copy number altered segments in the CN-300 cohort index cores. Regions ordered putatively ‘early’ (top) to ‘late’ alterations (bottom). Each bar divides the patients harbouring the specific genomic alteration (total number of patients annotated at the end of each bar) into burden of copy number alteration quartile groups. Regions of interest are represented by chromosome number, genomic location and cytoband (blue=copy number loss, pink=copy number gain). Regions containing known prostate cancer genes of interest are listed as follows: 8:23.4–24 (*NXK3.1*), 10:89.5–90 (*PTEN*), 13:48.5–49 (*RB1*), 17:7.5–8 (*TP53*), 8:128.5–129 (*cMYC*), 5:98–98.5 (*CHD1*). **C** Density plots demonstrating distribution of burden of copy number (CN) alteration (%) identified in the index core of patients with and without 8p segment deletions (low KS distance). All CN-300 patients harbouring 8:13–13.5 (8p22) and/or 8:11.5–12 (8p23) segment deletions are represented by a blue line versus no 8p22 and 8p23 segment deletion represented by a black line (8p22 deletion *N*=220, 8p23 deletion *N*=236). **D** Density plots demonstrating two regions with a high KS score that are associated with a higher burden of CN alteration (%); 8:128.5–129 (8q24 harbours *cMYC*) and 5:98–98.5 (5q21 harbours *CHD1*). All CN-300 patients harbouring 8:128.5–129 (8q24) gain are represented by a red line (*N*=145) versus no alteration at that segment (black line). All CN-300 patients harbouring 5:98–98.5 (5q21) loss are represented by a blue line (*N*=79) versus no alteration at that segment (black line)

We then hypothesised that copy number alteration showed an order with higher relative frequencies of specific alterations, putatively occurring early in carcinogenesis, in tumours with a low burden of copy number alteration. Similarly, we hypothesised that high burdens of copy number alteration would associate with specific alterations that either conferred survival advantages to prostate cancer cells in highly disordered cancers or contributed to increased copy number instability. Enrichment in tumours at low burdens of copy number alteration would present at similar frequencies across all copy number burdens. We found this to be the case notably for deletions of segments across 8p21-23 that occurred at a similar frequency in the first relative to second, third and fourth quartiles (Fig. 4A, B). This aligns with models using clonality assessment that suggested deletions at 8p21, involving *NKX3.1*, are common clonal and putatively early events [27]. Intermediate events such as loss of regions including other tumour suppressor genes such as *PTEN* (10q23), *TP53* (17p13) and *RB1* (13q14) [2, 27], occurred at progressively increasing frequencies across quartiles, showing the largest increase in frequency between the first and second quartile. Finally, to identify regions occurring at different frequencies across copy number states, we used the Kolmogorov–Smirnov (KS) test to compare the distributions of copy number burden of tumours with and without each of the commonly occurring (>20%) alterations (Additional file 1: Fig. S11). We identified that loss of segments in 5q21-22 (KS distance 0.5, adjusted *P*<0.0001) and gains at segments in 8q21-24 (KS distance 0.5–0.6 and adjusted *P*<0.0001) were amongst the most significant. These two regions most notably include *CHD1* and *cMYC* that have been shown to contribute to genome instability [28] (Fig. 4C, D).

### Variability of copy number alterations in multi-region sequencing of individual tumours

**Fig. 5:**
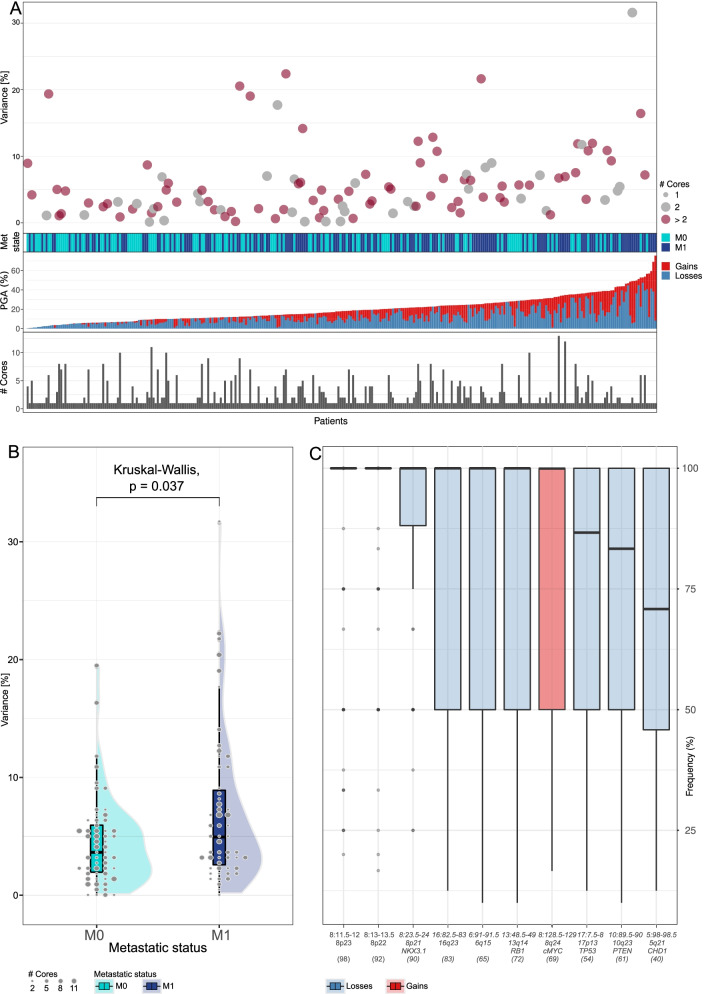
Variance in copy number alteration across multi-region diagnostic cores. **A** For a sub-set of 112 patients within the CN-300 cohort, we were able to copy number profile multiple diagnostic core biopsies from the same prostate (*N*=500, median 4 diagnostic core biopsies per patient). We calculated the variance in burden of copy number alteration (%) defined as the standard deviation across cores from the same prostate, represented as a dot. The colour and size of the dot represents the number of diagnostic cores copy number profiled per patient (grey=2, brown=>2) ranked in ascending order of burden of copy number alteration identified in the index core. Metastatic status of each patient is annotated (green=non-metastatic, blue=metastatic). Burden of copy number alteration (PGA=percentage genome altered) is represented as a bar with each patient split by proportion of gain (red) and loss (blue). Bottom bar chart represents number of diagnostic cores sequenced per patient. **B** Distribution of variance (%) of burden of copy number alteration per patient compared between non-metastatic (green violin plot) and metastatic (blue violin plot). Dot size represents number of cores sequenced per patient. **C** Boxplot demonstrating intra-patient heterogeneity of selected regions of interest. Regions annotated on *x*-axis labelled with chromosome number and genomic location mapped to cytoband. Blue=segment loss, red=segment gain. Within each bar, patients are only included if we sequenced more than one core and they harbour at least one core with the annotated alteration (numbers of patients annotated beneath *x*-axis). *Y*-axis represents the percentage of cores within patients harbouring the alteration and the boxplot line represents the median across patients

We finally sought to determine whether copy number alterations evolved uniformly across an individual’s primary tumour. From a sub-set of 112 patients with multiple diagnostic cores available (57 metastatic and 55 non-metastatic), we dissected multiple tumour-enriched areas per patient across separate diagnostic prostate biopsies (500 biopsies, median 4 biopsies per patient, range 2–13). We observed patients with considerable variation in the burden of copy number alteration and therefore calculated the variance, defined as the standard deviation between biopsies taken from the same prostate (Fig. 5A). We found no strong evidence of an association between copy number burden variance and biopsy number (Spearman *R*=0.15, *P*=0.12). Given copy number heterogeneity can associate with worse outcome in other cancer types [6, 29, 30], we performed an exploratory analysis and observed that the variance in copy number burden was significantly higher in metastatic compared to non-metastatic patients (Fig. 5B, Kruskal-Wallis test *P*=0.037). As the copy number alteration variance across cores and total burden of copy number alteration identified in the index core may be related (Spearman *R*=0.71, *P*<0.0001), we have here not tested the association for variance and outcome (Additional file 1: Fig. S12). Additionally, when a deletion of a segment in chromosome 8p21 was detected in a core, consistent with reports that this is an early event in carcinogenesis, we found it occurred in every core in that prostate whereas when a putatively later event such as a deletion in 5q21 occurred, it occurred in a median of 70.8% cores per case (interquartile range 45.8–100%) and prevalence was more heterogeneous (Fig. 5C).

## Discussion

We make the novel observation that the burden of copy number alteration is non-linearly associated with risk of clinical progression and death in advanced prostate cancer, with an initial sharp increase in relative risk that reaches a plateau and could represent a threshold effect. Similarly, the ratio of gains to losses increases in the first and second quartiles of copy number burden but is equivalent in the third and fourth. This leads us to propose a model that supports ordered accumulation of copy number change that is similar across metastatic states: at low copy number burden, we observe distinct ‘early’ events, primarily involving losses, with incremental increases in the frequency of intermediate events in tumours with higher copy number burden, most notably between the first and second quartiles. This overlaps with the initial worsening followed by a plateau of relative risk/hazard with increasing burden of copy number alteration. Of note, loss of individual genes in these regions (including *PTEN*, *RB1* and *TP53*) has been shown to be prognostic, but not independently of copy number burden [9].

As our study is cross-sectional across advanced cancers at diagnosis, we were unable to explicitly define temporal order. We split cancers into quartiles based on the proportion of the genome affected by copy number change, but this does not imply incremental accumulation of alterations: it is possible that a single disruptive event could result in a large increase in copy number burden [27]. Nonetheless, our results align with prior cross-sectional studies using clonality to define temporal order and, additionally, we observe that tumours in the highest quartile of copy number burden are more likely to have deletion at 5q21. Whereas this order may result in cell survival advantages for specific alterations at distinct stages of cancer progression, loss of specific genes such as *CHD1* may directly contribute to an increase in copy number burden [28].

To link copy number profiling to mature clinical follow-up, we have accepted a number of limitations. We used small amounts of diagnostic biopsy tissue of variable quality and subject to DNA formaldehyde artefacts. We obtained somatic mutation calls for a sub-set of cancers but as we reported previously, a high failure rate using current research and commercial assays limited integration of mutation with copy number calls [31]. We have focused on implementing a robust and scalable assay for analysing segmental copy number change and used microscopically dissected regions enriched for tumour. Our approach assumes stable tumour ploidy and may miss focal alterations and copy number neutral structural rearrangements that can disrupt gene structure and function. Nonetheless, the prevalence of alterations of commonly altered genes is similar in our analyses to prior studies [9, 32]. Future studies could further split the low copy number burden cancers by mutation load or other structural events such as tandem duplicates that are putatively associated with worse outcome [33, 34]. These groups are uncommon in prostate cancer but may further emphasise the effect of copy number change on worse outcome. By calculating copy number change from the genome median, our focus is on events additional to whole genome doubling that is common in prostate cancer but remains of uncertain prognostic relevance.

## Conclusions

Our study builds on prior copy number assessments in less advanced disease that showed copy number burden could have prognostic utility [5, 10]; our analysis of advanced prostate cancer with long-term follow-up has identified that accumulation of a relatively limited number of non-focal copy number alterations is associated with most of the increase in the relative risk of disease progression and death. In conclusion, we propose that copy number burden should be controlled for when evaluating individual gene alterations and could be further evaluated in prognostic tests for risk stratification of aggressive disease.

## Supplementary Information

**Additional file 1.** Contains supplemental figures 1-12.

**Additional file 2: Table S1.** STAMPEDE trial sites contributing patients to the CN-300 cohort. **Table S2.** Cohort characteristics of the CN-300 biomarker cohort compared to the full trial comparison group. **Table S3.** Survival at 4 years follow-up for different metastatic states (Kaplan-Meier estimates). **Table S4.** Number of events within the CN-300 cohort within trial endpoints. **Table S5.** Summary of estimated association between copy number burden and hazard of outcome from univariable and multivariable survival modelling. **Table S6.** Summary of estimated association between copy number burden and hazard of outcome from multivariable survival models, including interaction with metastatic status.

**Additional file 3.** Contains supplemental methods-full list of STAMPEDE trial eligibility criteria and criteria for inclusion in the CN-300 cohort.

**Additional file 4.** Genome segments for the CN-300 cohort.

**Additional file 5.** List of STAMPEDE investigators.

## Data Availability

The low coverage whole genome sequencing data for all samples analysed in this study is accessible at the European genome-phenome archive website under the accession number EGAD00001008800 [21].

## Abbreviations

ADT: Androgen deprivation therapy
ALT: Alanine aminotransferase
AST: Aspartate aminotransferase
AR: Androgen receptor
Bp: Base pair
CHAARTED: Chemohormonal Therapy Versus Androgen Ablation Randomized Trial for Extensive Disease in Prostate Cancer
CI: Confidence interval
CN: Copy number
CT: Computerised tomography scan
DNA: Deoxyribonucleic acid
FFPE: Formalin fixed paraffin embedded
FFS: Failure-free survival
FP: Fractional polynomial
GFR: Glomerular filtration rate
HR: Hazard ratio
IQR: Interquartile range
kb: Kilobase
KS: Kolmogorov–Smirnov
Ln: Natural logarithm transformation
LR: Likelihood ratio
M0: Non-metastatic
M1: Metastatic
M0N0: Non-metastatic and no local lymph node involvement
M0N1: Metastatic and local lymph node involvement
M1 high: High volume metastatic
M1 low: Low volume metastatic
MPFS: Metastatic progression-free survival
MRC CTU: Medical Research Council Clinical Trial Unit
NYHA: New York Heart Association
OS: Overall survival
PCSS: Prostate cancer-specific survival
PFS: Progression-free survival
PSA: Prostate-specific antigen
REC: Research ethics committee
Rtx: Radiotherapy
STAMPEDE: Systemic Therapy in Advancing or Metastatic Prostate cancer: Evaluation of Drug Efficacy
STRATOSPHERE: STratification for RAtional Treatment-Oncomarker Pairings of STAMPEDE patients starting long-term Hormone treatment
ULN: Upper limit of normal
WCB: Wales Cancer Bank
WHO: World Health Organization
