# Supplementary material for: Accumulation of copy number alterations and clinical progression across advanced prostate cancer
Source: Genome Med. 2022 Sep 5;14:102. doi: 10.1186/s13073-022-01080-4 (PMC9442998; doi:10.1186/s13073-022-01080-4)
Supplement: Supplementary file 1 — Additional file 1. Contains supplemental figures 1-12. [file 13073_2022_1080_MOESM1_ESM.pdf]

SUPPLEMENTAL FIGURES

Fig S1. STAMPEDE trial schema

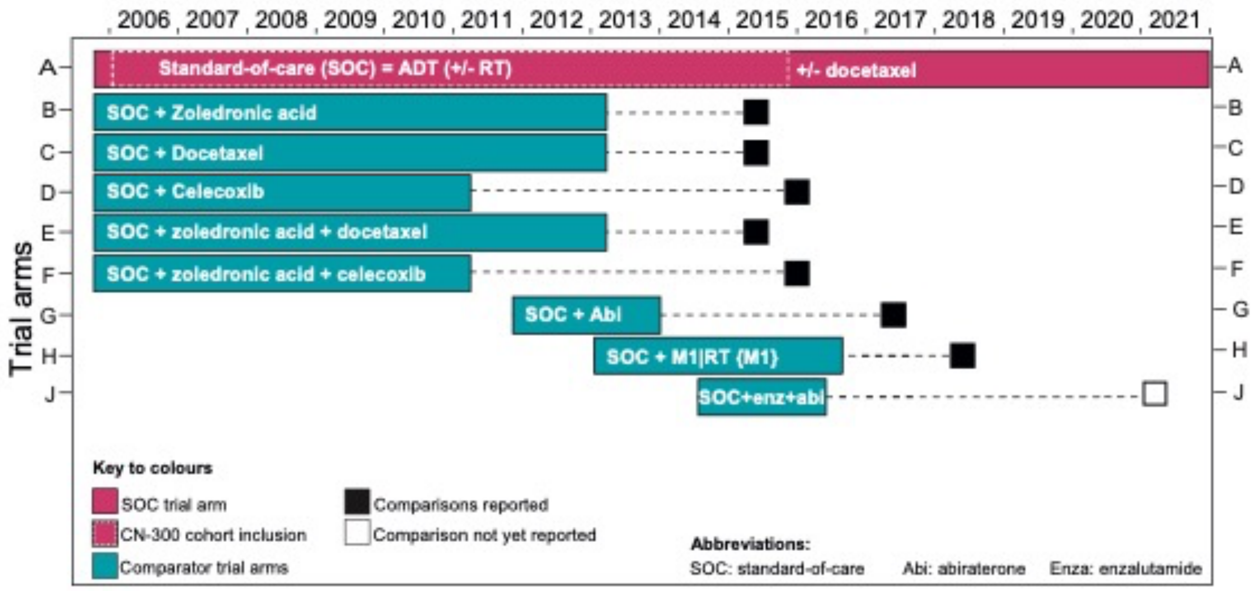

Fig S1. Sample flow chart

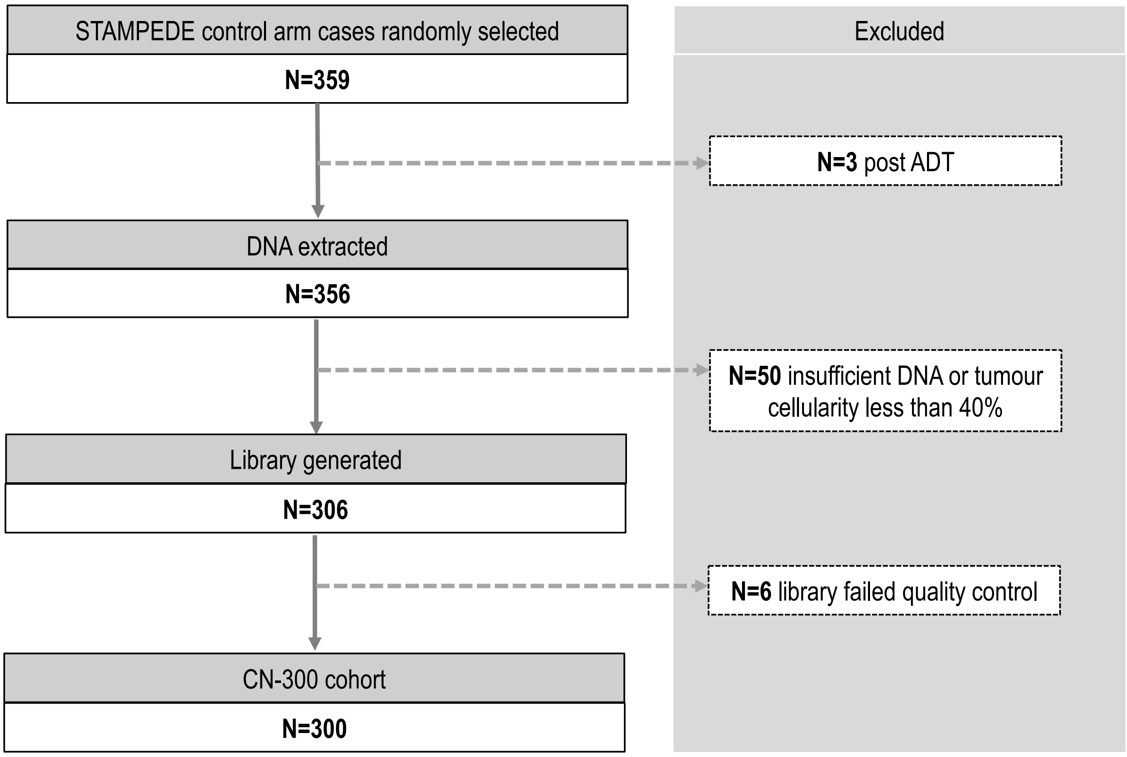

Fig S2. Boxplot demonstrating tumour cellularity (%) of index diagnostic core in CN-300 cohort

(A) Metastatic (M1) and non-metastatic (M0) cases (M0 median=80% versus M1 median=70%; range 40%-100%, Kruskal-Wallis *P*=0.011) and (B) split further into different metastatic states; non-metastatic and no local lymph node involvement (M0N0 median=80%); non-metastatic with local lymph node involvement (M0N1 median=70%); low volume metastatic (M1 low median=70%); high volume metastatic (M1 high median= 70%). Range across all metastatic states 40%-100%; Kruskal-Wallis *P*=0.1

(A)

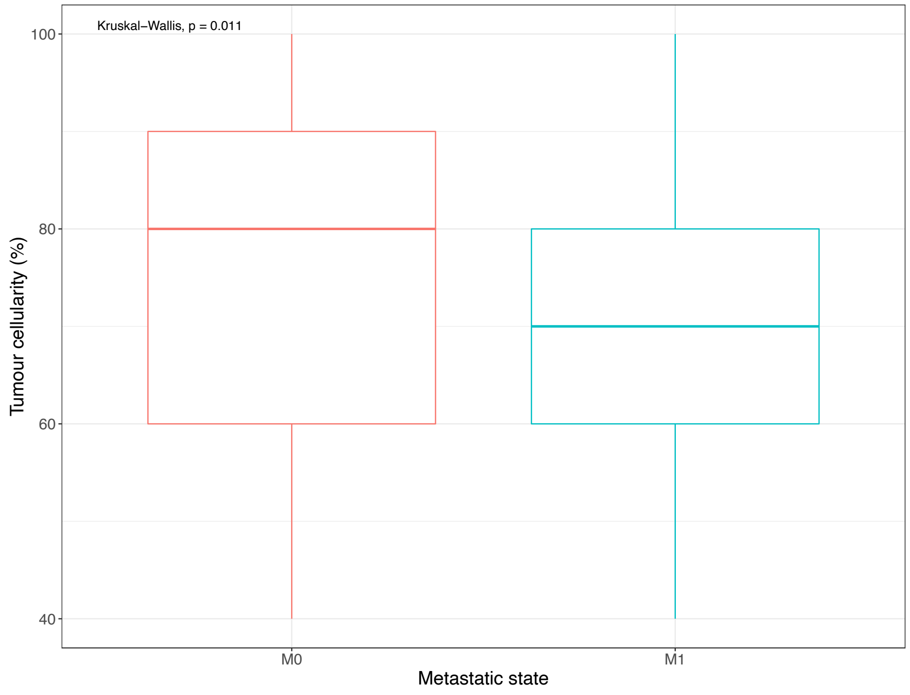

(B)

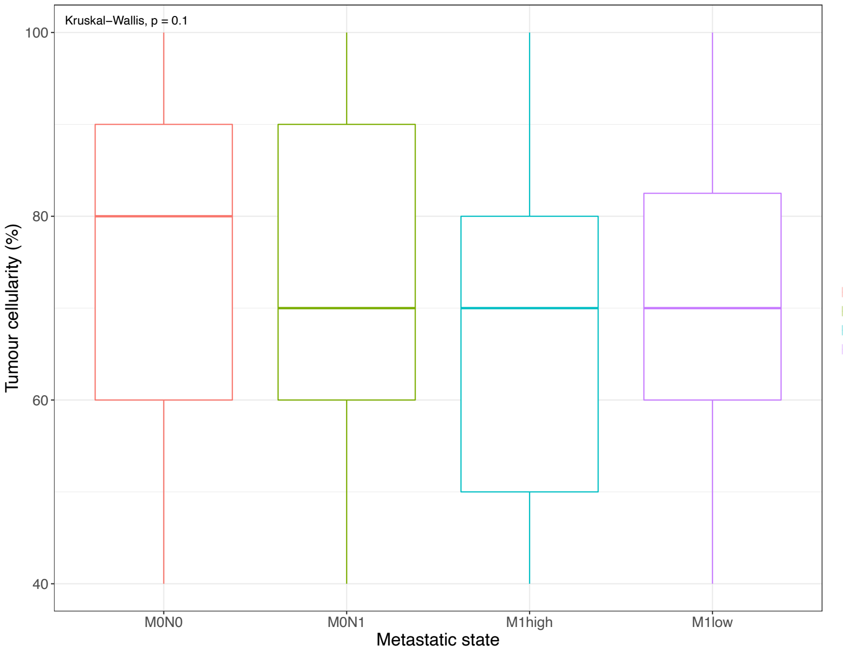
Kruskal–Wallis, p = 0.1
100
80
60
40
Tumour cellularity (%)
M0N0
M0N1
M1high
M1low
Metastatic state

Fig S3. Kaplan-Meier estimates of time-to-event (months) within each metastatic state

(A) Failure-free survival (FFS); (B) Progression-free survival (PFS); (C) Metastatic progression-free survival (MPFS); (D) Prostate cancer specific survival (PCa-specific survival); (E) Overall survival (OS)

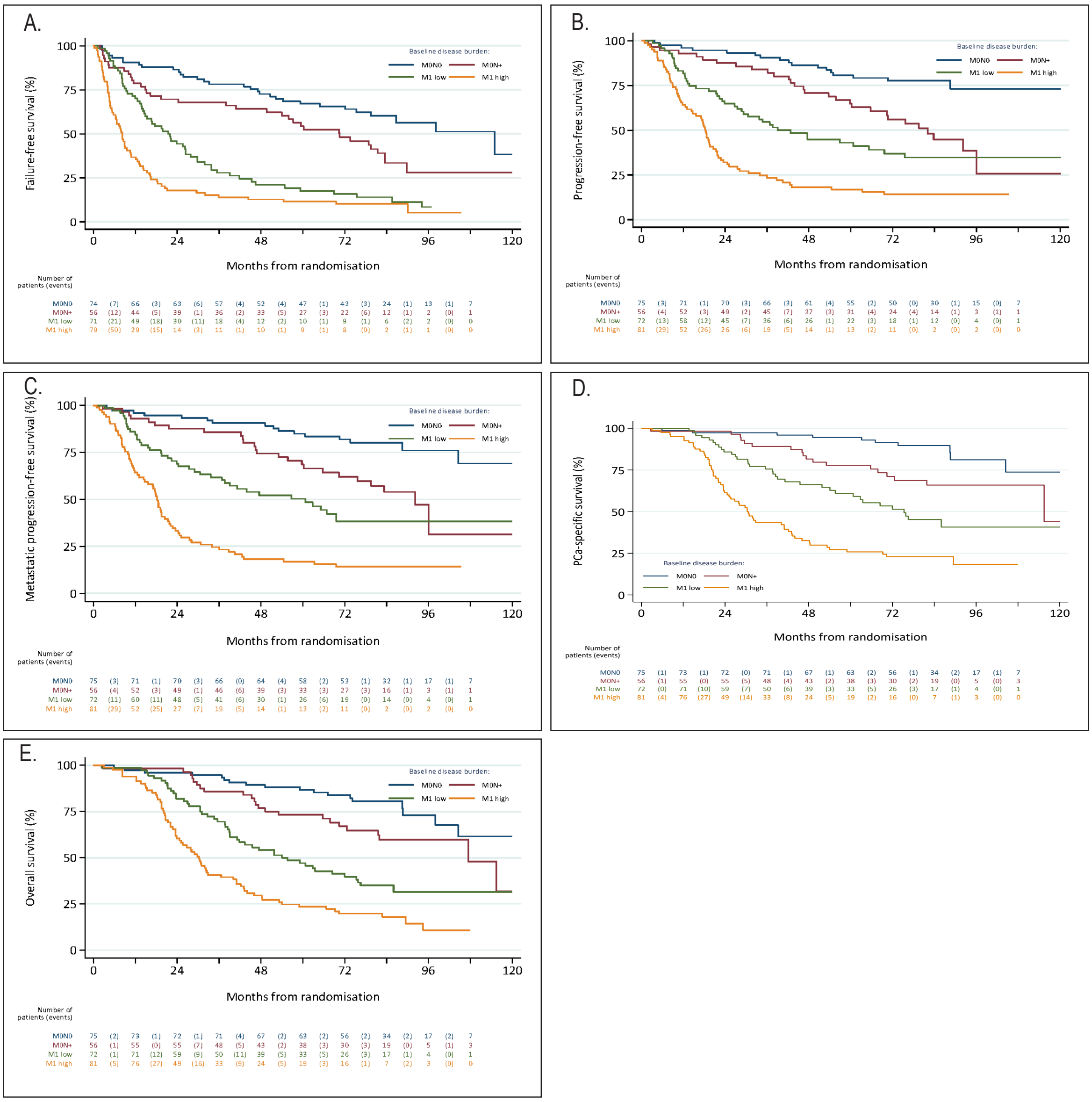

Fig S4. Sample coverage

(A) Modelling demonstrating 1-5X coverage of unique reads can be achieved with a minimum of 10ng DNA input. All samples included from the CN-300 cohort sequenced across eight Illumina Novaseq flow cells with DNA input ranging from 10-60ng depending on available DNA extracted from tumour enriched regions. X-axis=reads increased by a factor of 1-20 for each sequencing run and y-axis=coverage (unique reads) achieved. (B) Scatterplot of mean coverage and duplication rate (%) for 300 index core samples. Spearman correlation coefficient and *P*-value presented

(A)

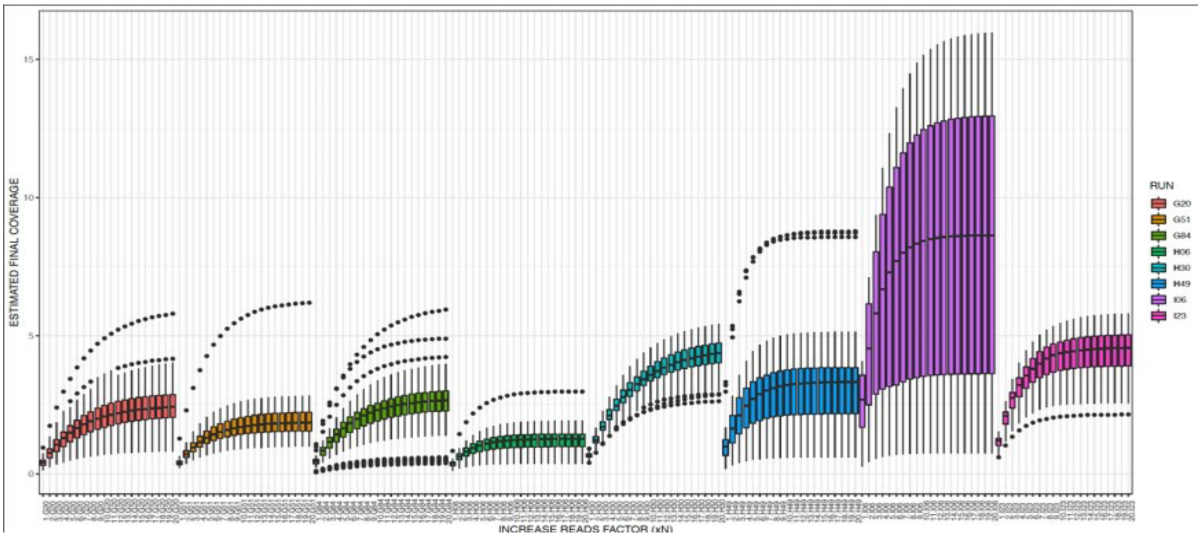

(B)

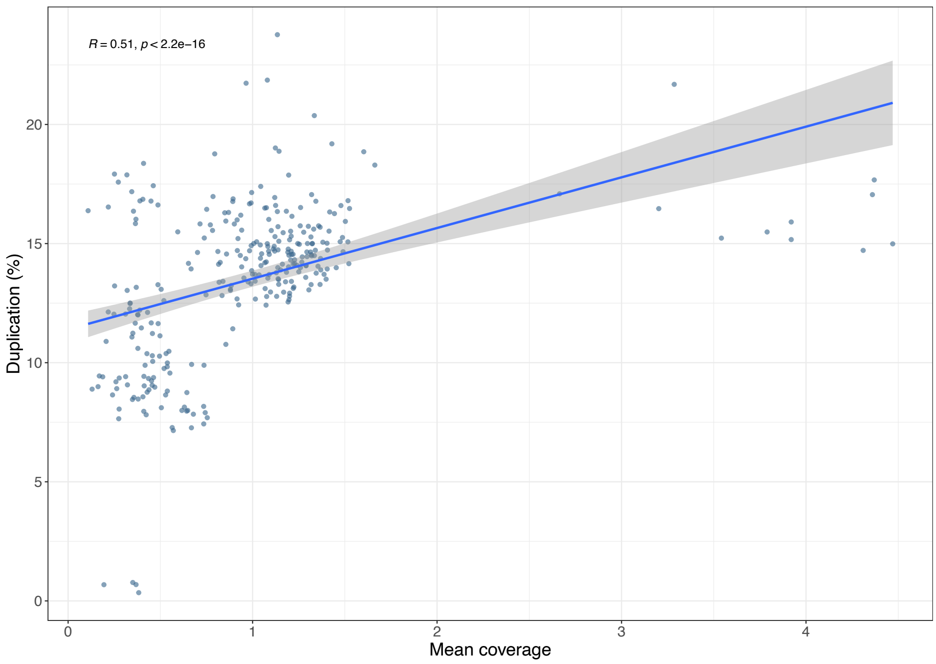

Fig S5. Burden of copy number (CN) alteration (%) in the index core of each case in the CN-300 cohort

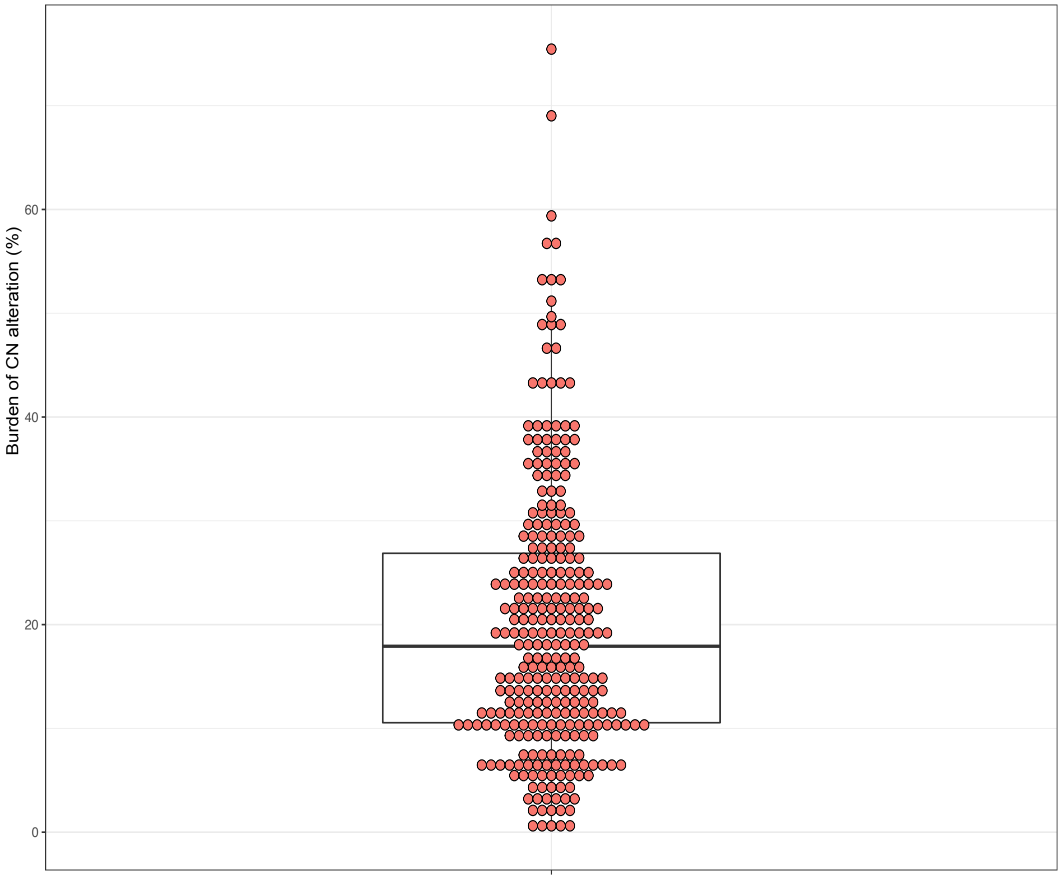

Fig S6. Association between mean segment copy number standard deviation per index core and technical variable

(A) Burden of copy number (CN) alteration (%); (B) tumour cellularity (%); (C) DNA input into library generation workflow (ng); (D) block age; (E) percentage of genome 1X coverage. Spearman correlation coefficient and *P*-value reported for each

(A)

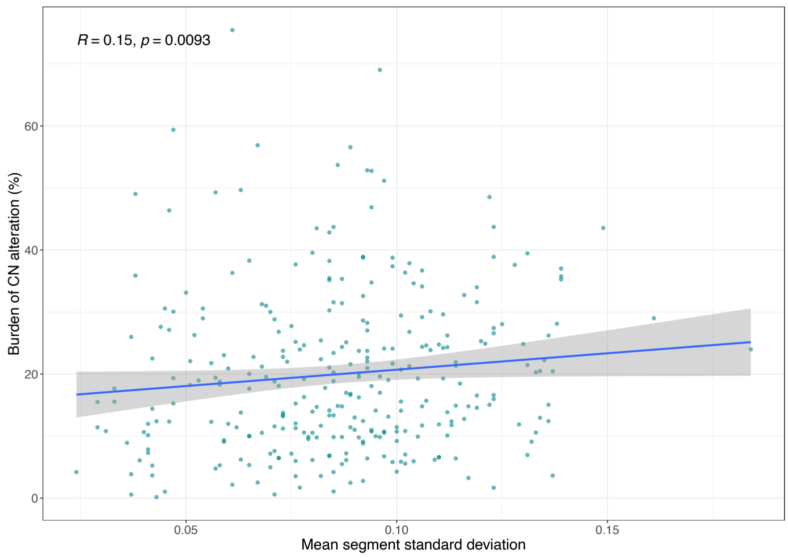

(B)

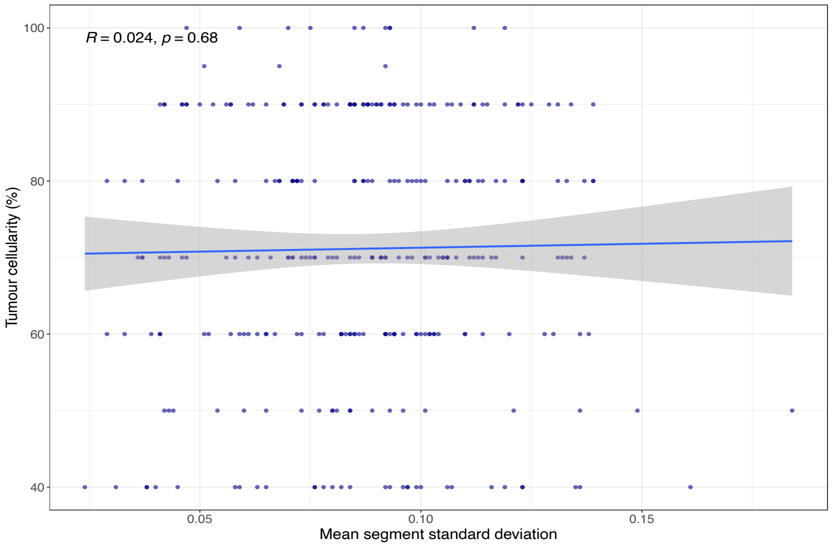

(C)

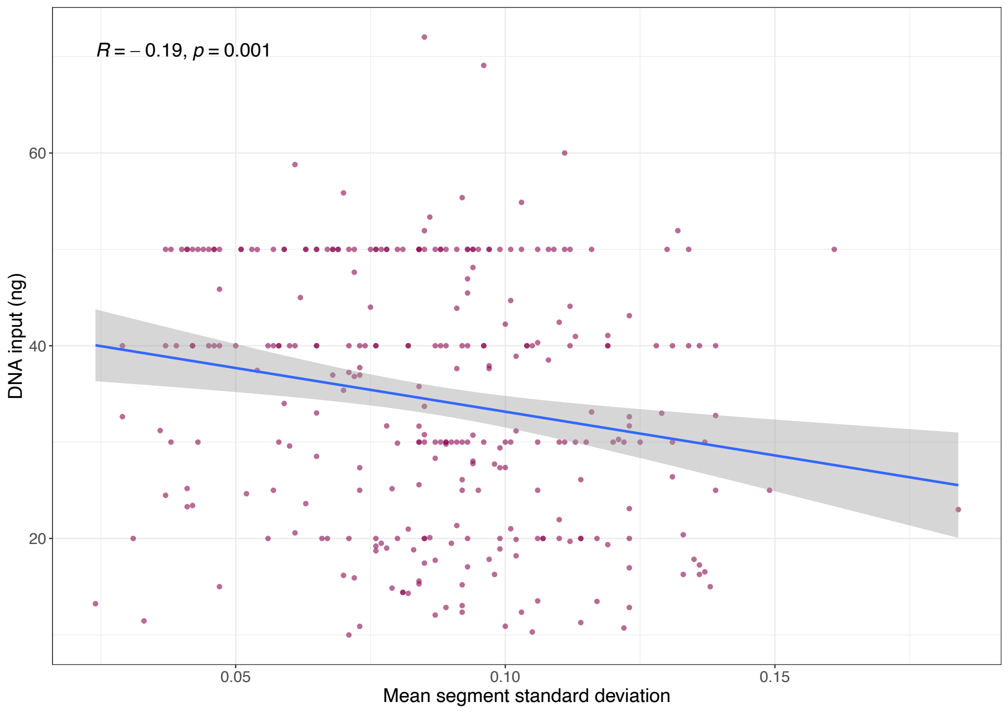
R = − 0.19, p = 0.001
DNA input (ng)
60
40
20
0.05
0.10
0.15
Mean segment standard deviation

(D)

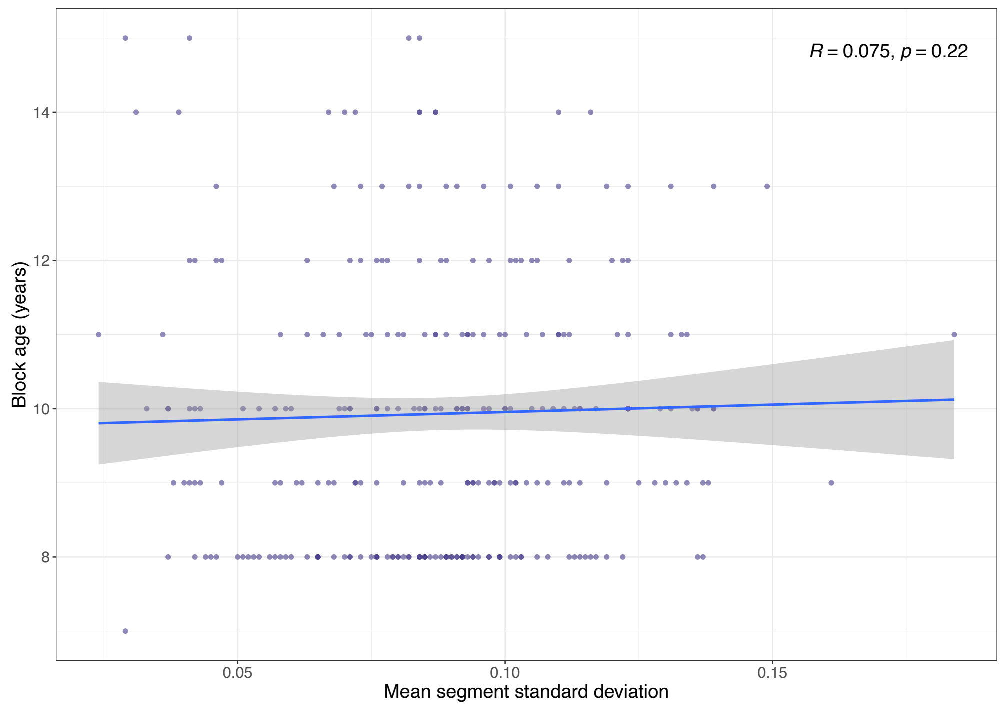
R = 0.075, p = 0.22
Block age (years)
14
12
10
8
0.05
0.10
0.15
Mean segment standard deviation

(E)

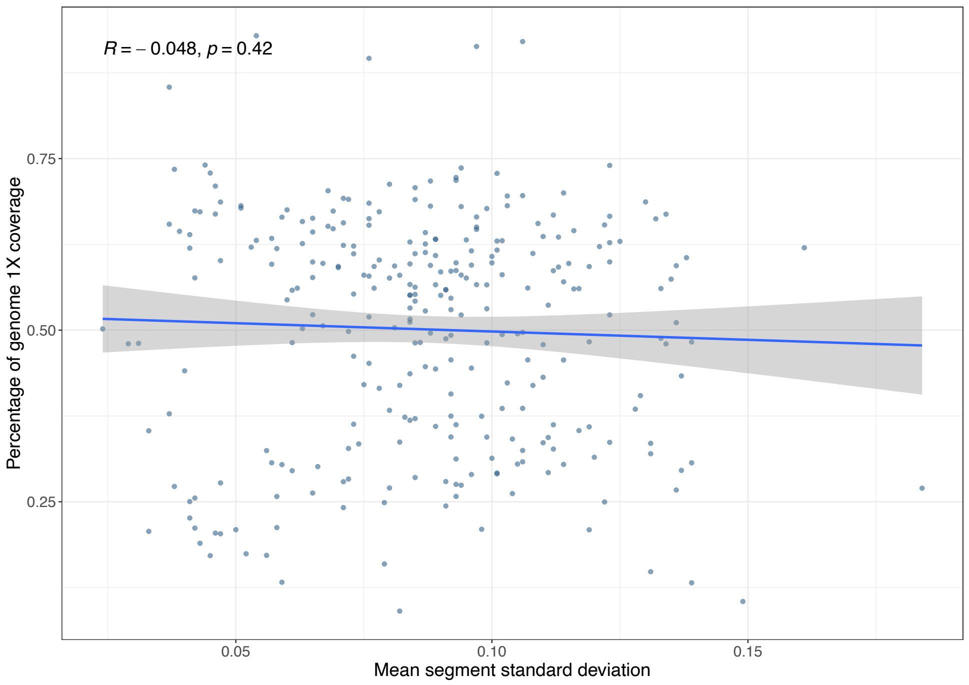

R = − 0.048, p = 0.42
Percentage of genome 1X coverage
0.75
0.50
0.25
0.05
0.10
0.15
Mean segment standard deviation

Fig S7. Association between the burden of copy number (CN) alteration (%) and technical variables;

(A) tumour cellularity of index core (%); (B) DNA input (ng) to library generation workflow; (C) block age; (D) the percentage of genome 1X coverage. Spearman correlation coefficient and *P*-value presented

(A)

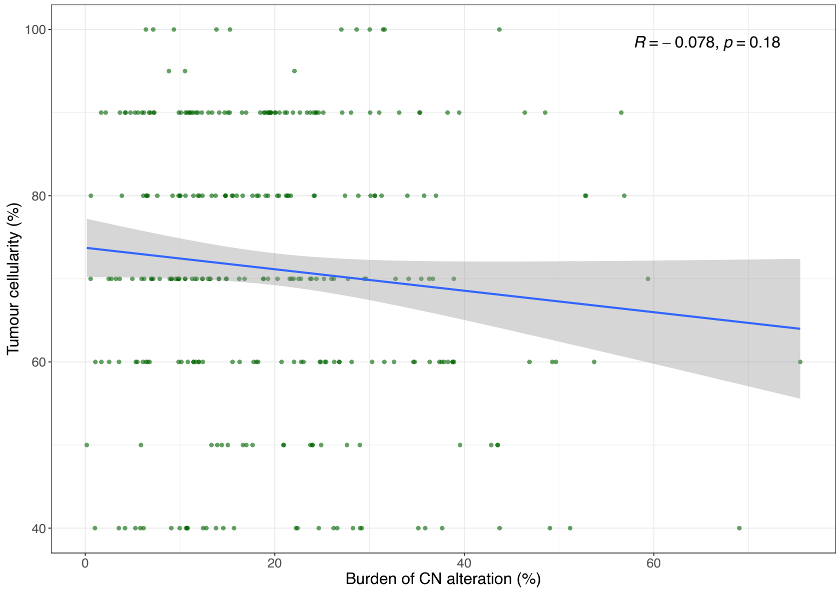

(B)

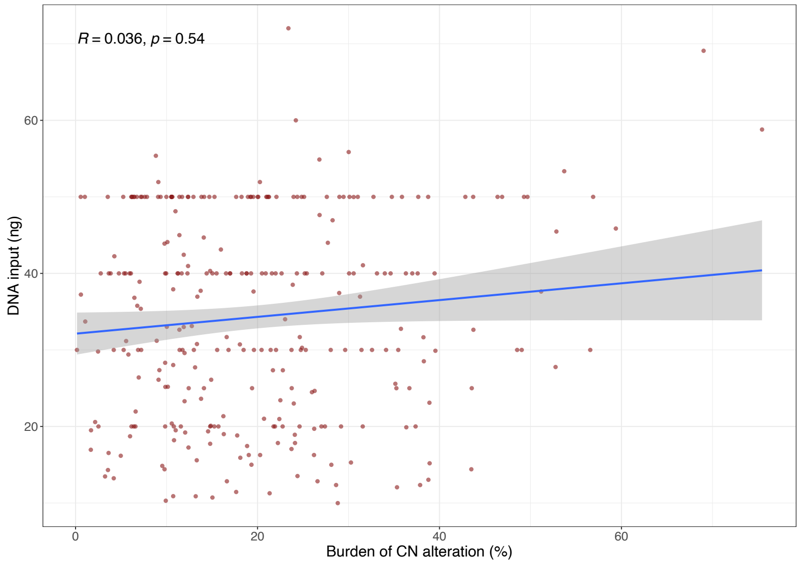

(C)

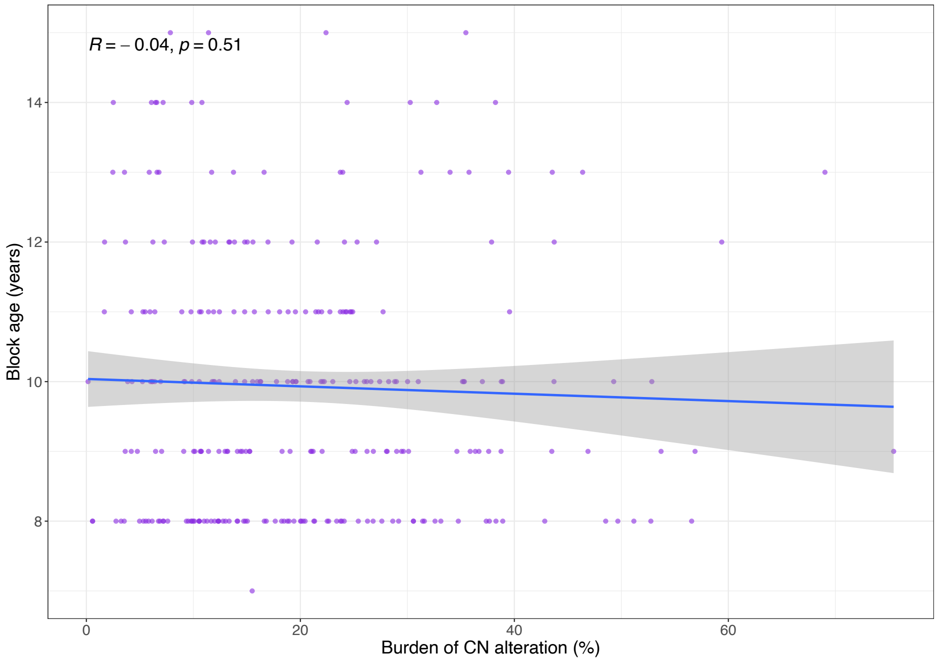
R = − 0.04, p = 0.51
Block age (years)
14
12
10
8
0
20
40
60
Burden of CN alteration (%)

(D)

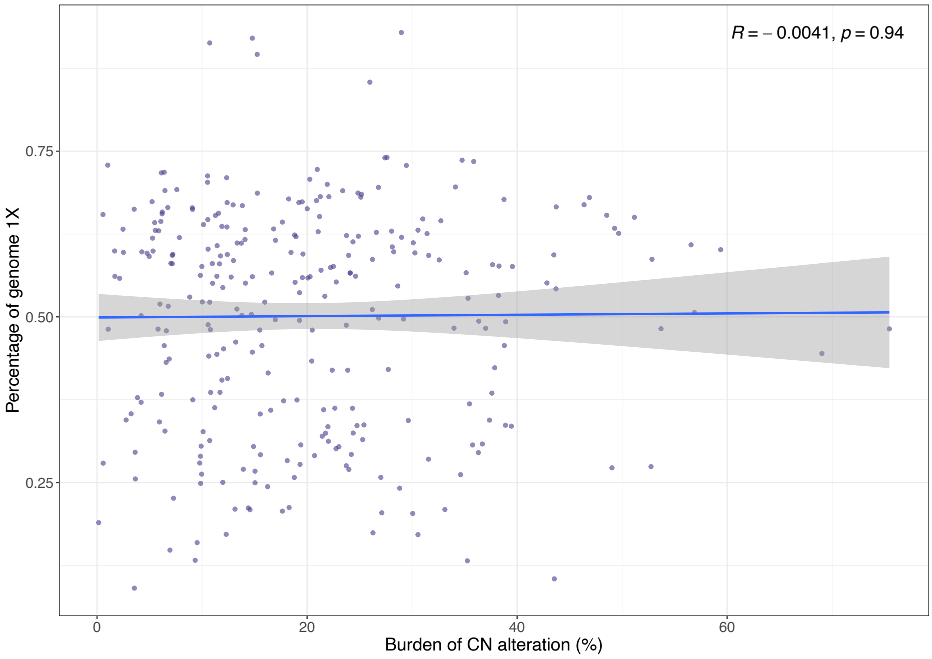
R = − 0.0041, p = 0.94
Percentage of genome 1X
0.75
0.50
0.25
0
20
40
60
Burden of CN alteration (%)

Fig S8. Association between burden of copy number (CN) alteration (%) and grading group for CN-300 cohort

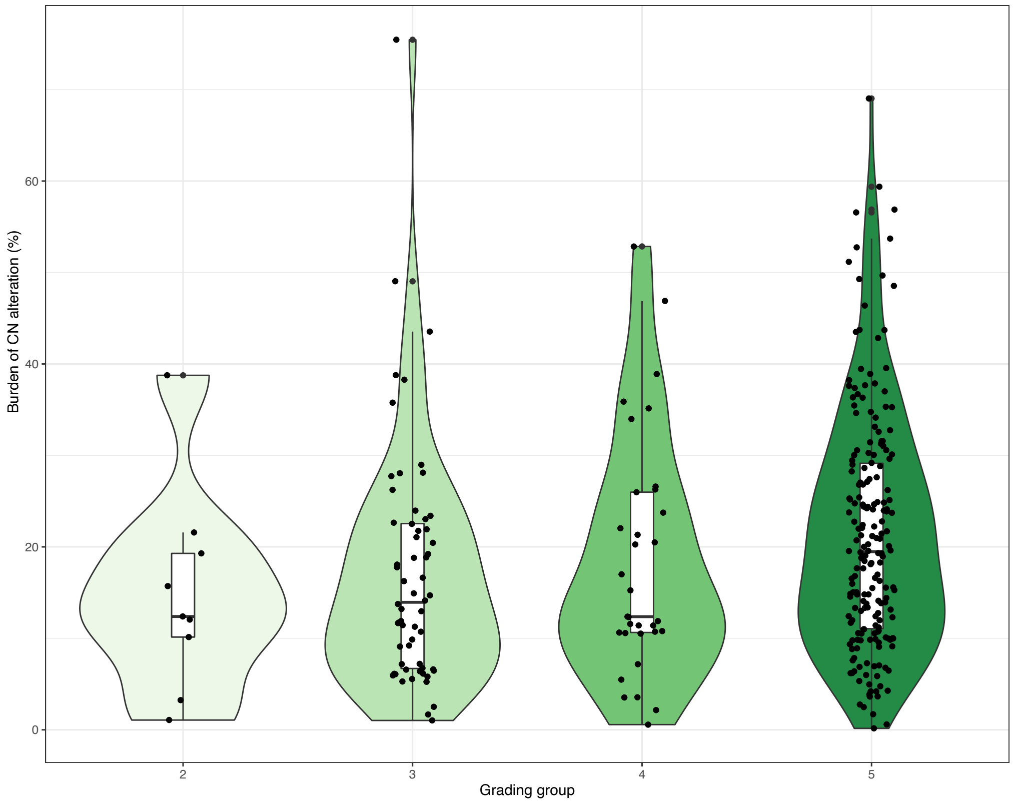

Fig S9. Univariable analyses association of burden of copy number alteration with clinical outcome

(A) Failure-free survival (FFS); (B) Metastatic progression-free survival (MPFS); (C) Prostate cancer specific survival (PCSS); (D) Overall survival (OS)

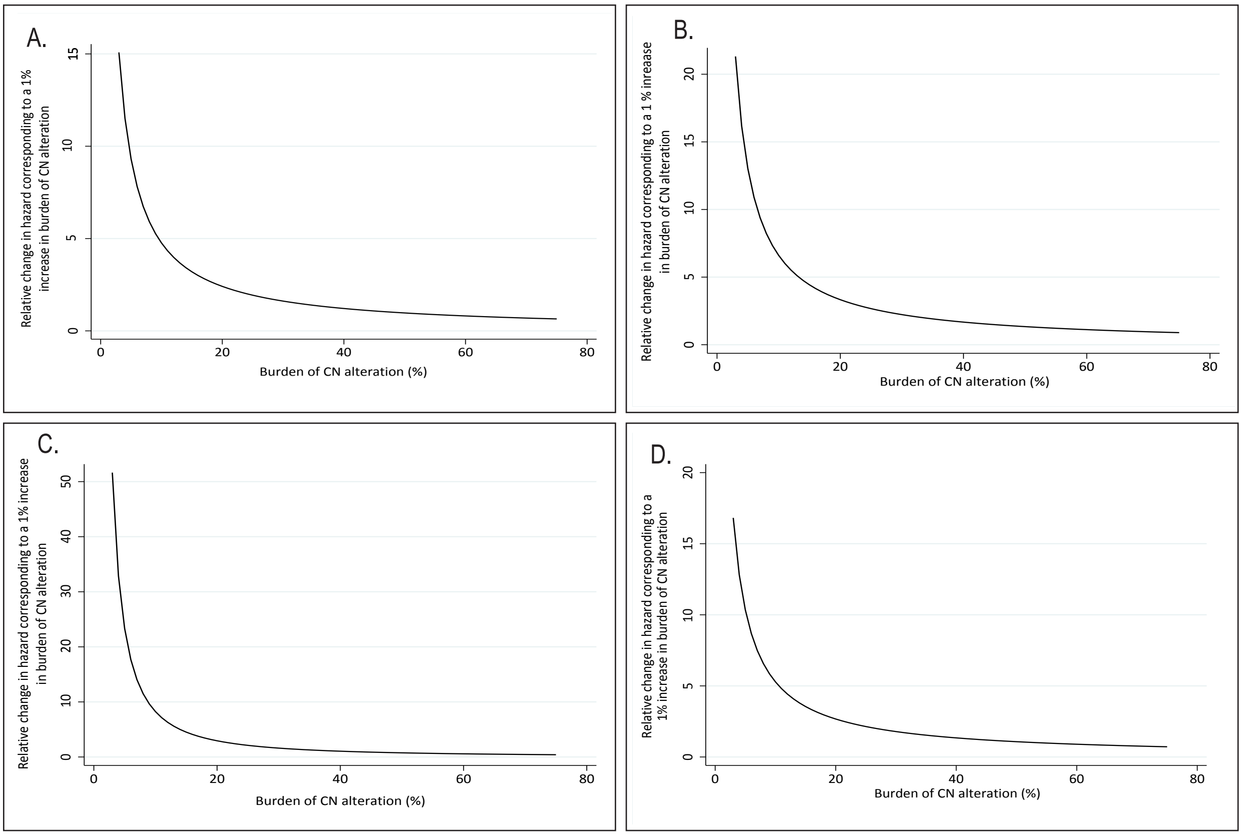

Fig S101. Summary of Kolmogorov-Smirnov test statistics

Bar chart of cytobands harbouring copy number (CN) alteration either gain (pink) or loss (blue) in at least 20% of CN-300 index cores, associated with a Kolmogorov-Smirnov (KS) distance >0.4 when compared with the overall distribution of CN alteration. Density refers to the q-value associated with each KS test

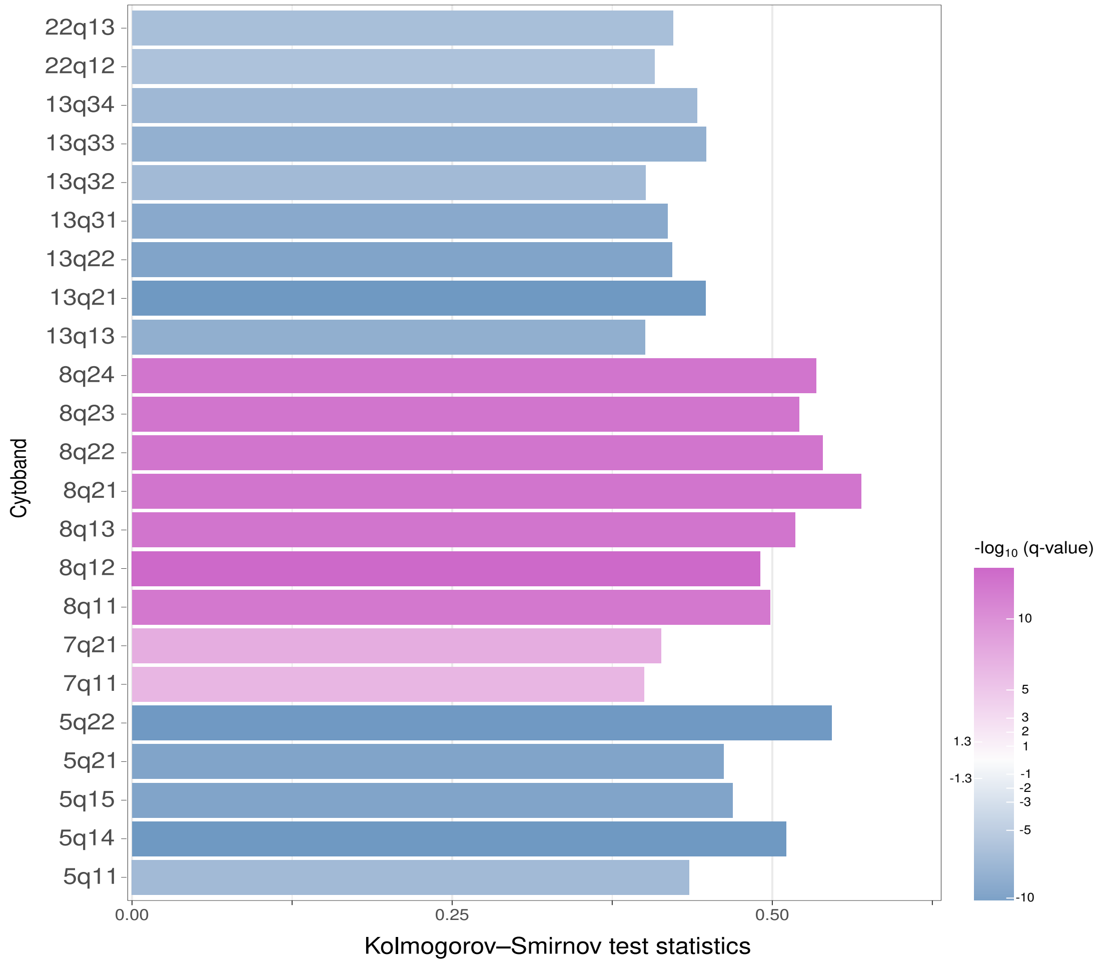

Fig S12. Variance in the burden of copy number alteration per patient

(A) Scatter plot demonstrating association between variance in burden of copy number alteration across multiple cores from the same case and the burden of copy number alteration identified in the index core per case. Size and colour of dot equates to the number of cores sequenced per case; (B) Demonstration of burden of copy number alteration across cores from the same case. 112 cases with more than or equal to two cores available to be sequenced included. Dot size represents Gleason grade and colour represents tumour cellularity (%). Index core per case outlined in red

(A)

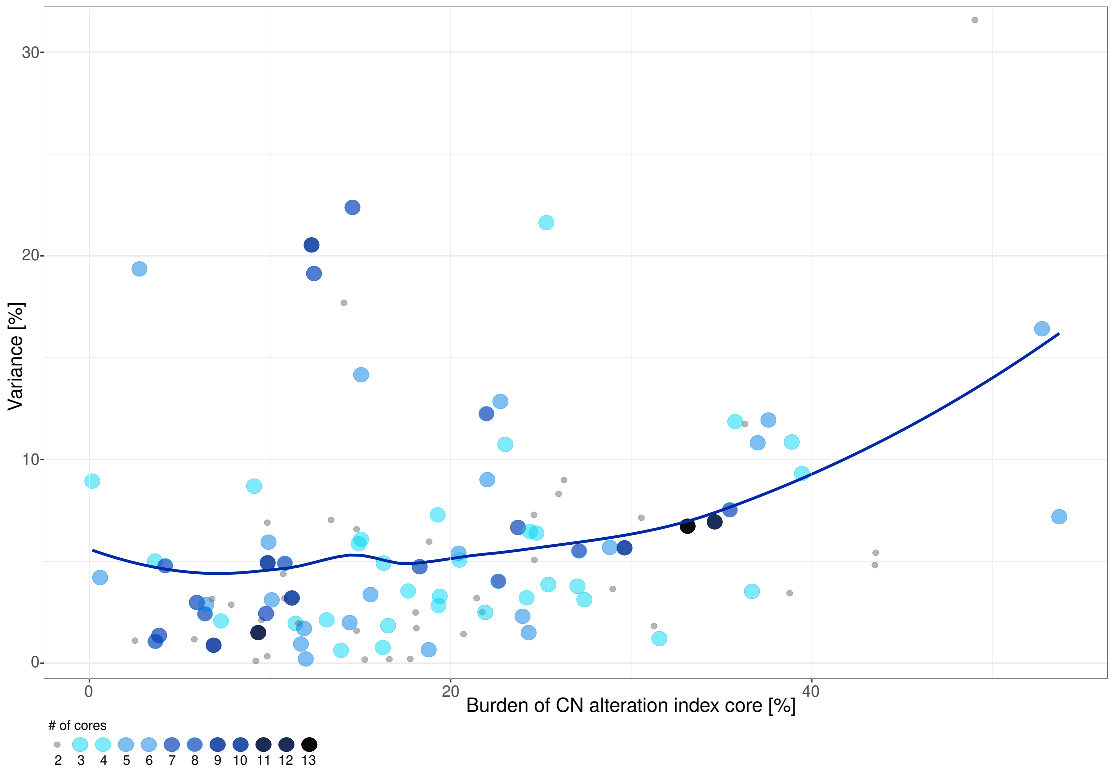

(B)

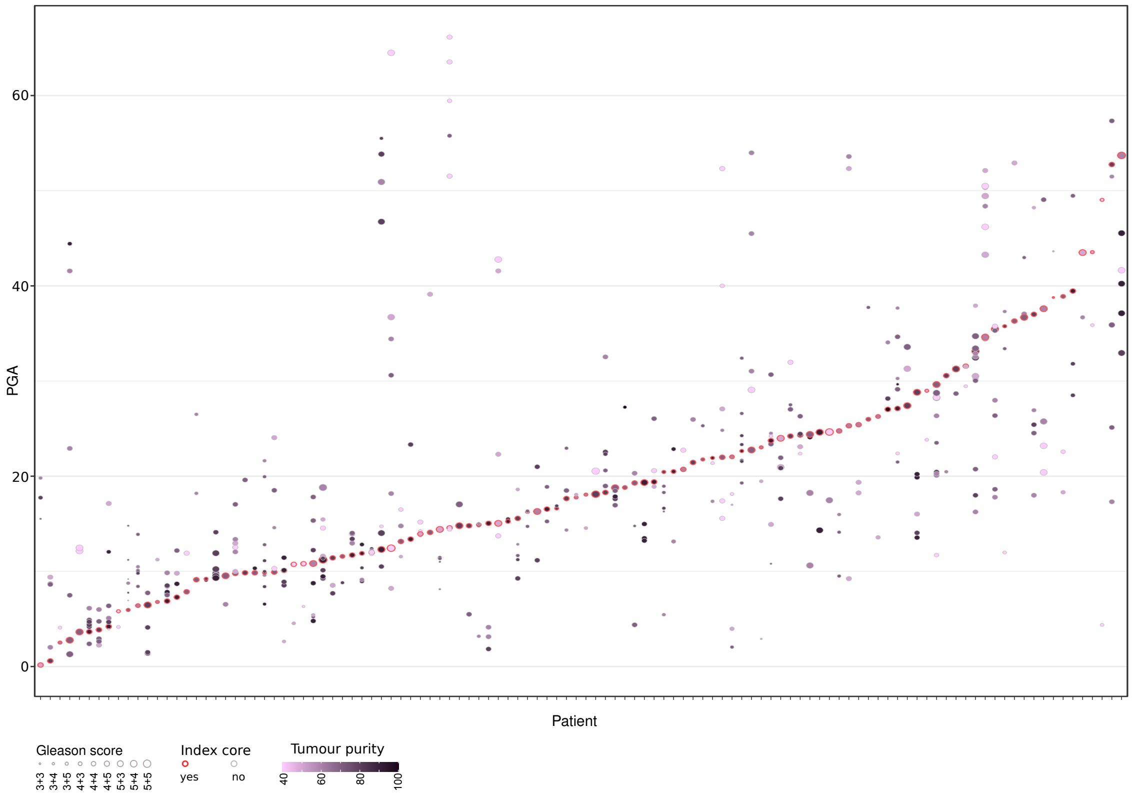

PGA
60
40
20
0
Patient
Gleason score
3+3
3+4
3+5
4+3
4+4
4+5
5+3
5+4
5+5
Index core
yes
no
Tumour purity
40
60
80
100
