# Supplementary material for: Accumulation of copy number alterations and clinical progression across advanced prostate cancer
Source: Genome Med. 2022 Sep 5;14:102. doi: 10.1186/s13073-022-01080-4 (PMC9442998; doi:10.1186/s13073-022-01080-4)
Supplement: Supplementary file 2 — Additional file 2: Table S1. STAMPEDE trial sites contributing patients to the CN-300 cohort. Table S2. Cohort characteristics of the CN-300 biomarker cohort compared to the full trial comparison group. Table S3. Survival at 4 years follow-up for different metastatic states (Kaplan-Meier estimates). Table S4. Number of events within the CN-300 cohort within trial endpoints. Table S5. Summary of estimated association between copy number burden and hazard of outcome from univariable and multivariable survival modelling. Table S6. Summary of estimated association between copy number burden and hazard of outcome from multivariable survival models, including interaction with metastatic status. [file 13073_2022_1080_MOESM2_ESM.pdf]

SUPPLEMENTAL TABLES

Table S1. STAMPEDE trial sites contributing patients to the CN-300 cohort

Trial sites contributing to CN-300 cohort
Cardiff, Velindre Cancer Centre
Hull, Castle Hill Hospital
Exeter, Royal Devon and Exeter Hospital NHS Trust
Manchester, The Christie NHS Foundation Trust
Swansea, Singleton Hospital
Belfast, Belfast City Hospital
London, Guy's and St Thomas' NHS Foundation Trust
Glasgow, Beatson Oncology Centre (BOC)
Southampton, Southampton General Hospital
Sunderland Tyne and Wear, Sunderland Royal Hospital
Torbay District, Torbay and South Devon NHS Foundation Trust
Shropshire, Shrewsbury and Telford Hospital NHS Trust
Bournemouth, Royal Bournemouth Hospital
Swindon, Great Western Hospital
Bristol, Bristol Haematology and Oncology Centre
Maidstone, Maidstone and Tunbridge Wells NHS Trust Kent Oncology Centre
Staffordshire, Queen's Hospital Burton
Guildford, Royal Surrey County Hospital
Middlesbrough, The James Cook University Hospital
Essex, Broomfield Hospital - Mid Essex Hospital Trust
Northwood, Mount Vernon Hospital
University College London Hospitals NHS Foundation Trust
Suffolk, Ipswich Hospital - East Suffolk and North Essex NHS Foundation Trust
Huddersfield, Huddersfield Royal Infirmary
Stockport, Stepping Hill Hospital
Hereford, The County Hospital
West Sussex, Worthing Hospital
Hampshire, Basingstoke & North Hampshire Hospital
London, North Middlesex University Hospital
Buckinghamshire, Wycombe Hospital Buckinghamshire Healthcare NHS Trust
Somerset, Musgrove Park Hospital
East Sussex, Eastbourne District General Hospital
Durham, Darlington Memorial Hospital

Hertfordshire - Stevenage, Lister Hospital
Farnworth, Royal Bolton Hospital
Inverness, Raigmore Hospital
Nottingham University Hospitals (City Campus)
Blackburn, East Lancashire NHS Trust
West Yorkshire, Airedale General Hospital
West Midlands, Russells Hall Hospital
Great Manchester, Royal Albert Edward Infirmary
Cheshire, Warrington Hospital
Kent, Kent and Canterbury Hospital
London, St Georges Hospital
Bath, Royal united Hospital
Ayr, University Hospital Ayr
London, Royal Free Hospital
Portsmouth, Queen Alexandra Hospital
Chester, Countess of Chester Hospital NHS Foundation Trust
North Devon, North Devon District Hospital
Liverpool, Royal Liverpool University Hospital
Bury St Edmunds, West Suffolk Hospital
Sutton-in-Ashfield, King's Mill Hospital
Birmingham, Birmingham City Hospital
Liverpool, Aintree University Hospitals NHS Foundation Trust
Somerset, Weston General Hospital
Lincolnshire, Lincoln County Hospital
Lincolnshire, Pilgrim Hospital Boston

Table S2. Cohort characteristics of the CN-300 biomarker cohort compared to the full trial comparison group

Characteristic		Biomarker (n=300)	Trial comparison (n=3106)	p-value*
Age at randomisation	Median (IQR)	67.0 (62.5 - 72.0)	67.0 (62.0 - 72.0)	0.97
(years)	Range	43.0 - 82.0	37.0 - 86.0	
Pre-ADT PSA	Median (IQR)	59.4 (22.6 - 158.0)	59.7 (20.4 - 171.5)	0.76
(ng/ml)	Range	2.7 - 7019.0	0.1 - 20590.0	
WHO Performance	0	238 (79%)	2374 (76%)	0.51
Status	1	60 (20%)	703 (23%)	
	2	2 (1%)	29 (1%)	
Disease burden	M0N0	75 (26%)	797 (28%)	0.55
	M0N+	56 (20%)	502 (18%)	
	M1 Low	72 (25%)	650 (23%)	
	M1 High	81 (29%)	884 (31%)	
	*Missing^*	*16*	*273*	
Tumour stage	T0	1 (<1%)	9 (<1%)	0.2
	T1	4 (1%)	36 (1%)	
	T2	37 (13%)	249 (9%)	
	T3	195 (67%)	2073 (71%)	
	T4	53 (18%)	554 (19%)	
	Tx	10	185	
Grade Group#	1	7 (2%)	66 (2%)	0.18
	2	29 (10%)	227 (8%)	
	3	42 (14%)	363 (12%)	
	4	80 (27%)	700 (23%)	
	5	141 (47%)	1626 (55%)	
	Missing	*1*	*124*	
RTx planned at randomisation	No	195 (65%)	2040 (66%)	0.81
	Yes	105 (35%)	1066 (34%)	
Recurrent vs	Recurrent	10 (3%)	132 (4%)	0.45
de novo	De novo	290 (97%)	2974 (96%)	
Pain	Absent	251 (84%)	2661 (86%)	0.19
	Present	49 (16%)	418 (14%)	
	Missing	*0*	*27*	

Legend: * 2-sided P-value from unequal variance t-test of null hypothesis of equal distributions provided for age and pre-ADT PSA; 2-sided P-value from Pearson's chi-

squared test of independence given for all other variables. ^ M1, volume classification unknown. # Determined from primary and secondary Gleason score recorded by treating site, based on local histopathology.

Table S3. Survival at 4 years follow-up for different metastatic states (Kaplan-Meier estimates)

Clinical endpoint	Estimated survival (%)			
	**M0N0**	**M0N1**	**M1 low**	**M1 high**
**Failure-Free Survival**	73	64	21	13
**Progression-Free Survival**	86	71	45	18
**Metastatic Progression-Free Survival**	91	74	52	18
**Prostate Cancer Specific Survival**	96	82	66	33
**Overall Survival**	89	77	54	30

Legend: Non-metastatic and no local lymph node involvement (M0N0), non-metastatic with local lymph node involvement (M0N1), low volume metastatic (M1 low) and high volume metastatic (M1 high)

Table S4. Number of events within the CN-300 cohort within trial endpoints

Clinical endpoint	Number of events (percentage of CN-300 cohort)
**Failure-Free Survival**	217 (72)
**Progression-Free Survival**	170 (57)
**Metastatic Progression-Free Survival**	161 (54)
**Prostate Cancer Specific Survival**	132 (44)
**Overall Survival**	164 (55)

Table S5. Summary of estimated association between copy number burden and hazard of outcome from univariable and multivariable survival modelling

Clinical event	Analysis	Power transformation *	LR *P*-value from comparison of simpler model with linear specification for PGA	HR	95% CI	*P*- value
**FFS**	Univariable	Ln	0.003	1.629	1.352-1.964	$4.57X10^{-8}$
	Multivariable	-0.5	0.027	0.222	0.064-0.766	0.0043
**MPFS**	Univariable	Ln	<0.001	1.957	1.558-2.459	$5.91X10^{-10}$
	Multivariable	-0.5	0.012	0.098	0.016-0.602	0.0026
**PCSS**	Univariable	-0.5	0.004	0.005	0.001-0.041	$4.87X10^{-9}$
	Multivariable	-1	0.095	0.036	0.001-1.433	0.011
**OS**	Univariable	Ln	0.001	1.717	1.376-2.142	$3.28X10^{-7}$
	Multivariable	-0.5	0.038	0.277	0.064-1.204	0.045

Legend: Multivariable analyses included 1) grading group 2) log PSA prior to starting ADT 3) age at randomisation (years) 4) metastatic status (M0N0, M0N1, M1 low and M1 high) and 5) tumour cellularity (%). Clinical events include failure-free survival (FFS), metastatic progression-free survival (MPFS), prostate cancer specific survival (PCSS) and overall survival (OS). Univariable analyses includes N=300, multivariable

analyses includes N=284 (16 cases in which burden of metastatic disease was not known have been excluded)

* Applied to variable representing burden of copy number alteration prior to model fitting; note that estimated hazard ratio, confidence interval and likelihood ratio (LR) test *P*-value apply to this transformed variable. Ln = natural logarithm transformation.

Table S6 Summary of estimated association between copy number burden and hazard of outcome from multivariable survival models, including interaction with metastatic status

Clinical event	Power transformation	*Interaction test *P*-value*	Metastatic status	HR	95% confidence interval
**FFS**	-0.5	0.955			
			M0N0	0.380	0.0310 - 4.66
			M0N1	0.141	0.00730 - 2.71
			M1 low	0.265	0.0231 - 3.03
			M1 high	0.176	0.0175 - 1.76
**MPFS**	-0.5	0.398			
			M0N0	0.00275	7.25 x 10-6; 1.04
			M0N1	0.227	0.00964 - 5.36
			M1 low	0.0484	0.00178 - 1.32
			M1 high	0.321	0.0351 - 2.93
**PCSS**	-1	0.950			
			M0N0	0.0247	2.61x10-6; 234
			M0N1	0.0787	7.64 x 10-5; 81.2
			M1 low	0.00837	8.81 x 10-6; 7.96
			M1 high	0.104	4.43 x 10-4; 24.5
**OS**	-0.5	0.851			
			M0N0	0.447	0.0206; 9.69
			M0N1	0.205	0.00602 - 6.97
			M1 low	0.0909	0.00357 - 2.32
			M1 high	0.455	0.0565 - 3.67

Legend: Summary of metastatic state interaction models. Clinical events include failure-free survival (FFS), metastatic progression-free survival (MPFS), prostate cancer specific survival (PCSS) and overall survival (OS)

* Result of likelihood ratio test comparing model with interaction terms included against nested model with no interaction specified
