# Supplementary material for: Accumulation of copy number alterations and clinical progression across advanced prostate cancer
Source: Genome Med. 2022 Sep 5;14:102. doi: 10.1186/s13073-022-01080-4 (PMC9442998; doi:10.1186/s13073-022-01080-4)
Supplement: Supplementary file 3 — Additional file 3. Contains supplemental methods-full list of STAMPEDE trial eligibility criteria and criteria for inclusion in the CN-300 cohort. [file 13073_2022_1080_MOESM3_ESM.pdf]

SUPPLEMENTAL METHODS

Stampede trial inclusion and exclusion criteria are as follows:

Inclusion criteria

Participants must fulfil all the criteria in one of the following three categories:

1. HIGH-RISK NEWLY DIAGNOSED NON-METASTATIC NODE-NEGATIVE DISEASE - Both:

1.1. At least two of: Stage T3/4, PSA ≥ 40 ng/ml or Gleason sum score 8-10

1.2. Intention to treat with radical radiotherapy (unless there is a contra-indication; exemption can sought in advance of consent, after discussion with MRC CTU)

OR

2. NEWLY DIAGNOSED METASTATIC OR NODE-POSITIVE DISEASE - At least one of:

2.1. Stage Tany N+ M0

2.2. Stage Tany Nany M+

OR

3. PREVIOUSLY TREATED WITH RADICAL SURGERY AND/OR RADIOTHERAPY, NOW RELAPSING - At least one of:

3.1. PSA ≥ 4 ng/ml and rising with doubling time less than 6 months

3.2. PSA ≥ 20 ng/ml

3.3. Lymph node positive

3.4. Metastatic disease

All patients were required to meet the following criteria:

- Histologically confirmed prostate adenocarcinoma
- Intention to treat with long-term androgen deprivation therapy
- Fit for all protocol treatment and follow-up
- WHO performance status 0-2
- Have completed the appropriate investigations prior to randomisation
- Adequate haematological function: neutrophil count ≥1.5x109 /l and platelets ≥100x109 /l
- Adequate renal function, defined as GFR ≥30ml/min/1.73m2
- Written informed consent
- Willing and expected to comply with follow-up schedule
- Using effective contraceptive method if applicable

Exclusion criteria:

- Prior systemic therapy for locally-advanced or metastatic prostate cancer
- Prior exposure to hormone therapy for a duration of > 12 months, or prior exposure completing < 12 months before randomisation
- Metastatic brain disease or leptomeningeal disease
- Abnormal liver functions consisting of any of the following:

  Serum bilirubin ≥1.5 x ULN (except for participants with Gilbert's disease, for whom the upper limit of serum bilirubin is 51.3µmol/l or 3mg/dl)

  Aspartate aminotransferase (AST) or alanine aminotransferase (ALT) ≥2.5 x ULN

- Any other previous or current malignant disease which, in the judgement of the responsible clinician, is likely to interfere with STAMPEDE treatment or assessment
- Any surgical wound which in the judgement of the responsible clinician may interfere with or be exacerbated by protocol treatment
- Participants with significant cardiovascular disease, including:
    - Severe/unstable angina
    - Myocardial infarction less than 6 months prior to randomisation
    - Arterial thrombotic events less than 6 months prior to randomisation
    - Clinically significant cardiac failure requiring treatment, defined as New York Heart Association (NYHA) class II or above
    - Cerebrovascular disease (e.g. stroke or transient ischaemic episode) less than 6 months prior to randomisation
    - Any other significant cardiovascular disease that in the investigator's opinion means the participant is unfit for any of the study treatments

Cases were randomly selected to generate copy number profiles if the following criteria were met:

- Consent obtained for the use of prostate tissue in additional research
- Randomised to the control arm of STAMPEDE to receive ADT, from UK trial sites
- Diagnostic FFPE tissue available for biomarker analysis

- More than 10ng DNA extracted from at least one diagnostic biopsy for low coverage WGS
