# Supplementary material for: Accumulation of copy number alterations and clinical progression across advanced prostate cancer
Source: Genome Med. 2022 Sep 5;14:102. doi: 10.1186/s13073-022-01080-4 (PMC9442998; doi:10.1186/s13073-022-01080-4)
Supplement: Supplementary file 5 — Additional file 5. List of STAMPEDE investigators. [file 13073_2022_1080_MOESM5_ESM.pdf]

INVESTIGATORS AND COLLABORATORS: SITE STAFF

Staff on site delegation logs

City	Care_Site	Person_Name	Site_PI
Abergavenny, UK	Nevill Hall Hospital	Christian Smith	
Aberystwyth, UK	Bronglais General Hospital	Elin Jones	PI
~	~	Russel Canavan	
~	~	Kirsty Marie Dennett	
~	~	Claire Duggan	
~	~	Sajid Durrani	
~	~	Bleddyn Edwards	
~	~	John Edwards	
~	~	Sandra Evens	
~	~	Abigail Hynes	
~	~	Basharat Jameel	
~	~	Gwenan Parry Jones	
~	~	Philip Jones	
~	~	Rhian Elin Jones	
~	~	Sarah Jones	
~	~	Christine Kotonya	
~	~	Ronda Loosley	
~	~	Heather McGuinness	
~	~	Cerith Morgan	
~	~	Geraint Morgan	
~	~	Mark Narain	
~	~	Emma Nurse	
~	~	Donna Robson	
~	~	Llinos Strange	
~	~	Helen Tench	
~	~	Sean Thomas	
~	~	Toby Frederick Trugeion-Smith	
~	~	Kenneth Richard Williams	
~	~	Rebecca Wolf-Roberts	
Ashford, UK	William Harvey Hospital	Carys Thomas	PI
~	~	Albert Edwards	Co-I
~	~	Jessica Little	Co-I
~	~	Natasha Mithal	Co-I

INVESTIGATORS AND COLLABORATORS: SITE STAFF

Staff on site delegation logs

City	Care_Site	Person_Name	Site_PI
~	~	Rakesh Raman	Co-I
~	~	Jennifer Turner	Co-I
~	~	Louise Allen	
~	~	Bonny Appleby	
~	~	Sharon Beesley	
~	~	Hayley Blackgrove	
~	~	Tracy Boakes	
~	~	Patryk Brulinski	
~	~	Julie Buckley	
~	~	Miguel Capo-Mir	
~	~	Natalie Catt	
~	~	Mathilda Cominos	
~	~	Denise Crawford	
~	~	Nikki Crisp	
~	~	Steve Dann	
~	~	Julie-Ann Davies	
~	~	Susan Drakeley	
~	~	Clary Evans	
~	~	Sam Gibson	
~	~	Andrew Gillian	
~	~	Louise Gladwell	
~	~	Coral Greenstreet	
~	~	Tessa Hammond	
~	~	Sandra Holness	
~	~	Laura Kehoe	
~	~	Sue Kelly	
~	~	Rachel Larkins	
~	~	Kathryn Lees	
~	~	Sarah Lightfoot	
~	~	Sarah Lines	
~	~	Margaret Lipsham	
~	~	Sydnie Loveland	
~	~	Rohit Malde	

INVESTIGATORS AND COLLABORATORS: SITE STAFF
Staff on site delegation logs

City	Care_Site	Person_Name	Site_PI
~	~	Kim Mears	
~	~	Sharon Middleton	
~	~	Arafat Mirza	
~	~	Kannon Nathan	
~	~	Udaiveer Panwar	
~	~	Claire Pelham	
~	~	Karen Robinson	
~	~	Susan Rogers	
~	~	Lesley Rose	
~	~	Cindy Slater	
~	~	Mathini Sridharan	
~	~	Stephane Tankoua	
~	~	Katy Taylor	
~	~	Kim Travis	
~	~	Alba Tubau	
~	~	Ifigenia Vasiliadou	
~	~	Kathleen (Kathy) Walsh	
~	~	Paula Whichelo	
~	~	Claire White	
~	~	Joanne Williams	
~	~	Elizabeth Williamson	
~	~	Victoria Williamson	
~	~	Marian Wood	
~	~	Linda Wray	
~	~	Hilary Zurakovsky	
Aylesbury, UK	Stoke Mandeville Hospital	Katherine Hyde	PI
~	~	Philip Camilleri	Co-I
~	~	Thinn Pwint	Co-I
~	~	Christopher Alcock	
~	~	Maggie Aldersley	
~	~	Gerard Andrade	
~	~	Bhavna Badiani	
~	~	Jasvinder Bains	

INVESTIGATORS AND COLLABORATORS: SITE STAFF

Staff on site delegation logs

City	Care_Site	Person_Name	Site_PI
~	~	Margaret Bowerbank	
~	~	Joanne Brady	
~	~	Chrissie Butcher	
~	~	Janice Carpenter	
~	~	Prabir Chakraborti	
~	~	Christine Collins	
~	~	Siobhan Gettings	
~	~	Jonathan Greenland	
~	~	Kathryn Herbert	
~	~	Iram Husain	
~	~	Manisha Joshi	
~	~	Roisin Kavanagh	
~	~	Rahul Kurup	
~	~	Rossana Mancinelli	
~	~	Sarah Manyangadze	
~	~	Moncy Mathew	
~	~	Alice Ngumo	
~	~	Sean O'Cathail	
~	~	Anna Osadcow	
~	~	Cheryl Padilla-Harris	
~	~	Niki Panakis	
~	~	Andrew Protheroe	
~	~	Ami Sabharwal	
~	~	Tracey Stammers	
~	~	Michelle Taylor-Siddons	
~	~	Andy Theobold	
~	~	Neil Trew-Smith	
~	~	Gail Varley	
~	~	Janet Weir	
~	~	Hazel Wynn	
Ayr, UK	Ayr Hospital	Hilary Glen	PI
~	~	Xia Ren	Co-I
~	~	Jawaher Ansari	

INVESTIGATORS AND COLLABORATORS: SITE STAFF

Staff on site delegation logs

City	Care_Site	Person_Name	Site_PI
~	~	Helena Belikova	
~	~	Philip Cannon	
~	~	Deborah Dunn	
~	~	Danielle Gilmour	
~	~	Dianne Hunter	
~	~	Ricky Hunter	
~	~	Jennifer Keith	
~	~	Esfandiyar Khan	
~	~	Christina Lai	
~	~	Kirsten Laws (nee Borthwick)	
~	~	Clare Love	
~	~	Nicholas Macleod	
~	~	Rana Mahmood	
~	~	Jane McClements	
~	~	Brian McGlynn	
~	~	David McIntosh	
~	~	Margaret McKernan	
~	~	Lynne McNeil	
~	~	Sharon Meehan	
~	~	Jenna Mitchell	
~	~	Rebecca Muirhead	
~	~	Alison Murphy	
~	~	Stefan Nowich	
~	~	Kirsty O'Hara	
~	~	Kristy Ross	
~	~	Kathleen Smith	
~	~	Maureen Templeton	
~	~	Lye Mun Tho	
~	~	Aisha Tufail	
~	~	Claudia Turley	
~	~	Susan Walton	
~	~	Elaine Watson	
~	~	Lillian White	

INVESTIGATORS AND COLLABORATORS: SITE STAFF

Staff on site delegation logs

City	Care_Site	Person_Name	Site_PI
~	~	Mark Wilson	
~	~	Diane Woodburn	
~	~	Danna Yorston	
Barnet, UK	Barnet General Hospital	Sarah Needleman	PI
~	~	Ursula McGovern	Ex-PI
~	~	Kimberley Durno	Co-I
~	~	Magdalena Kubiak	Co-I
~	~	Kate Smith	Co-I
~	~	Anita Amadi	
~	~	Alice Coady	
~	~	Danielle Collier	
~	~	Veronica Conteh	
~	~	Andie David	
~	~	Andrew Eichholz	
~	~	Christine Ellis	
~	~	Annette Hawkins	
~	~	Heather Hughes	
~	~	Gillian Marks	
~	~	Anita Mitra	
~	~	Panayiotis Panayiotou	
~	~	Prital Patel	
~	~	Emily Scott	
Barnstaple, UK	North Devon District Hospital	Denise Sheehan	PI
~	~	Victoria Ford	Co-I
~	~	Peter Stephens	Co-I
~	~	Lynsey Balmbra-Jenks	
~	~	Maria Beaumont	
~	~	Helen Black	
~	~	Andy Bull	
~	~	Susan Collard	
~	~	Jenna Furse	
~	~	Henry Goss	
~	~	Joshua Gregory	

INVESTIGATORS AND COLLABORATORS: SITE STAFF
Staff on site delegation logs

City	Care_Site	Person_Name	Site_PI
~	~	Laura Hanson	
~	~	Becky Holbrook	
~	~	Katherine Horder	
~	~	Faisal Hussain	
~	~	Natalie Kemp	
~	~	Elizabeth Kershaw	
~	~	Michal Ian Lamparski	
~	~	Samantha Ley	
~	~	Judyta Lomza	
~	~	Ajaz Lone	
~	~	Nyasha Manomano	
~	~	Maria Martinez	
~	~	Martin Moody	
~	~	Chantal Oelofse	
~	~	Eng Ong	
~	~	Hannah Ong	
~	~	Sarah Park	
~	~	Chloe Peters	
~	~	Rufus Smith	
~	~	Amy Thomas	
~	~	Fiona Thomas	
~	~	Elizabeth Toy	
~	~	Lynne Van Koutrik	
~	~	Lynne Van-Koutrik	
~	~	Faye Windsor	
Basingstoke, UK	Basingstoke and North Hampshire Hospital	Sangeeta Paisey	PI
~	~	Richard Shaffer	Ex-PI
~	~	Katherine Aitken	
~	~	David Barlow	
~	~	Nanda Basker	
~	~	Louise Beattie	
~	~	Godfrey Bownie-Mukumbu	
~	~	Rachel Bryan	

INVESTIGATORS AND COLLABORATORS: SITE STAFF

Staff on site delegation logs

City	Care_Site	Person_Name	Site_PI
~	~	Jo-Anna Conyngham	
~	~	Duncan Cooke	
~	~	Victoria Corner	
~	~	Abigail Edwards	
~	~	Sara Fawcitt	
~	~	Adrienn Fazekasne Fulep	
~	~	Angela Frith	
~	~	Teresa Guerrero-Urbano	
~	~	Julie Gwilt	
~	~	Liz Happle	
~	~	Roger Hudson	
~	~	Lauriane Kerwood	
~	~	Kathryn Leach (nee Noake)	
~	~	Eva Letalova	
~	~	Christina Narh	
~	~	Jenny Nobes	
~	~	Bintha Paruthickal	
~	~	Christine Podesta	
~	~	Pennie Porter	
~	~	Helen Richards	
~	~	Catherine Rimington	
~	~	Fasar Sarwar	
~	~	Jackie Smith	
~	~	Joanna Stokoe	
~	~	Sree Susaria	
~	~	Rao Vuyyuru	
~	~	Katharine Webb	
~	~	Rosalyne Westley	
~	~	Ingrid White	
~	~	Claire Williams	
~	~	Rebecca Wills	
~	~	Katie Wood	
~	~	Carmen Wu	

INVESTIGATORS AND COLLABORATORS: SITE STAFF
Staff on site delegation logs

City	Care_Site	Person_Name	Site_PI
~	~	Hilawati Yusof	
Bath, UK	Royal United Hospital	Mark Beresford	PI
~	~	Olivera Frim	Co-I
~	~	Catherine McDonald	Co-I
~	~	Nathalie Webber	Co-I
~	~	Tom Wilson	Co-I
~	~	Rowan Appleby	
~	~	Joanne Avis	
~	~	Gareth Ayre	
~	~	Claire Barron	
~	~	Hannah Blades	
~	~	Rachael Bolitho	
~	~	Ruth Brydon-Hill	
~	~	Shaolin Chidavaenzi	
~	~	Vicki Clarke	
~	~	Christine Cox	
~	~	Claire Craige	
~	~	Jane Crozier	
~	~	Samantha Curtis	
~	~	Michael Daly	
~	~	Jackie Davies	
~	~	Claire Davis	
~	~	Frances Du Feu	
~	~	Claire Dyke	
~	~	Christine Elwell	
~	~	Rachael Exley	
~	~	Yuko Francis	
~	~	Beatrice Hamilton	
~	~	Leonie Harrison	
~	~	Lorna Hawley	
~	~	Abigail Jenner	
~	~	Penny Kehagioglou	
~	~	Carly Laxon-Takooree	

INVESTIGATORS AND COLLABORATORS: SITE STAFF

Staff on site delegation logs

City	Care_Site	Person_Name	Site_PI
~	~	Guillaume Livera	
~	~	Jill MacDonald-Burn	
~	~	Katarzyna Machura	
~	~	Margaret Macmillan	
~	~	Susan Masson	
~	~	Carey Milsom (nee Logan)	
~	~	Kate Moloney	
~	~	Sarah Murdoch	
~	~	Joseph Needham	
~	~	Hugh Newman	
~	~	Abigail Pocock	
~	~	Vicki Portingale	
~	~	Bryony Robertson	
~	~	Matthew Sephton	
~	~	Eve Tomlinson	
~	~	Tom Tylee	
~	~	Kristelle Vassallo	
~	~	Rebecca Wassall	
~	~	Jess White	
~	~	Chris Williams	
~	~	Samantha Williams	
~	~	Tania Williams (Née Allen)	
~	~	Joanna Wilson	
Bebington, UK	Clatterbridge Centre for Oncology	Zafar Malik	PI
~	~	Azman Ibrahim	Co-I
~	~	Ian Allen	
~	~	Wesley Artist	
~	~	Lisa Dobson (nee Child)	
~	~	Caroline Dunn	
~	~	Sharon Dunn (nee Johnson)	
~	~	Annemieke Earnshaw	
~	~	Diane Fildes	
~	~	Helen Flint	

INVESTIGATORS AND COLLABORATORS: SITE STAFF
Staff on site delegation logs

City	Care_Site	Person_Name	Site_PI
~	~	Elizabeth Gallimore	
~	~	Pat Gillis	
~	~	Sue Green	
~	~	Paul Griffiths	
~	~	Elizabeth Harrison	
~	~	Alison Hassall	
~	~	Jodie Henderson	
~	~	Kathryn Hughes	
~	~	Jess Hulse	
~	~	Helen Innes	
~	~	John Littler	
~	~	Laurie Lomax	
~	~	Linda Lyons	
~	~	Suzanne Maloney	
~	~	Laura McAllister	
~	~	Amir Montazeri	
~	~	Priyank Patel	
~	~	Dawn Porter	
~	~	Sandra Robinson	
~	~	Peter Robson	
~	~	Katie Sloan	
~	~	Matthew Stott	
~	~	Isabel Syndikus	
~	~	Shaun Tolan	
~	~	Emma Whitby	
~	~	Burhan Zavery	
Belfast, UK	Belfast City Hospital	Joe O'Sullivan	PI
~	~	Suneil Jain	Co-I
~	~	Swati Ray	Co-I
~	~	Poh Lin Shum	Co-I
~	~	Melvyn Ang	
~	~	Ruth Boyd	
~	~	Ellen Brown	

INVESTIGATORS AND COLLABORATORS: SITE STAFF

Staff on site delegation logs

City	Care_Site	Person_Name	Site_PI
~	~	Aishleen Brunton	
~	~	Patricia Calisaya	
~	~	Karen Campfield	
~	~	Peter Clarke	
~	~	Aiden Cole	
~	~	Wendy Cunningham	
~	~	Benedict Dadebo	
~	~	Prantik Das	
~	~	Catherine Davidson	
~	~	Mairead Devine	
~	~	Eileen Dillon	
~	~	Geraldine Douris	
~	~	Ruth Eakin	
~	~	Rachel Ellis	
~	~	Rhun Evans	
~	~	Ciaran Fairmichael	
~	~	Rebecca Goody	
~	~	Chris Hagan	
~	~	Emma Hanna	
~	~	Michael Hanna	
~	~	Jackie Harney	
~	~	Barbara Harvey	
~	~	Eimear Henry	
~	~	Stacey Hetherington	
~	~	Naomi Hill	
~	~	Sharon Hynds	
~	~	Lucy Jellett	
~	~	Ruth Johnston	
~	~	Sai Jonnada	
~	~	Patrick Keane	
~	~	Grace Lavery	
~	~	Diane Law	
~	~	Alison Logie	

INVESTIGATORS AND COLLABORATORS: SITE STAFF

Staff on site delegation logs

City	Care_Site	Person_Name	Site_PI
~	~	Jonathan McAleese	
~	~	Chryelle McAlister	
~	~	Seosamh McCauley	
~	~	Sharon McClean	
~	~	Paula McCloskey	
~	~	Kairen McCloy	
~	~	Sara McCusker(nee Stokes)	
~	~	Sarah McGahey	
~	~	Ciara McIlmunn	
~	~	Karen McKenna	
~	~	Shirley McKenna	
~	~	Aine McKeown	
~	~	Michael McMahon	
~	~	Linda McNeice	
~	~	Darren Mitchell	
~	~	Laura Mooney	
~	~	Angela Morrison	
~	~	Lynsey Morrow	
~	~	Lois Mulholland	
~	~	Kerry Nicholls	
~	~	Adrina O'Donnell	
~	~	Karen Parsons	
~	~	Jemma Robinson	
~	~	Claire Rooney	
~	~	Keith Rooney	
~	~	Angela Rosbotham	
~	~	William Snelling	
~	~	David Stewart	
~	~	Stephen Stranex	
~	~	Fiona Tarpey	
~	~	Jonathan Thompson	
~	~	Joanne Todd	
~	~	Phil Turner	

INVESTIGATORS AND COLLABORATORS: SITE STAFF
Staff on site delegation logs

City	Care_Site	Person_Name	Site_PI
~	~	Salil Vengalil	
Birmingham, UK	Birmingham Heartlands Hospital	Anjali Zarkar	PI
~	~	Kamaldeep Ajimal	
~	~	Chen Bartlett	
~	~	Madhura Chandrashekara	
~	~	Ellen Drew	
~	~	Mary (Ellen) Drew	
~	~	Penny Goodby (nee Harbach	
~	~	Samarah Haq	
~	~	Adrian Kelly	
~	~	Jill Lyons	
~	~	Alison Maidment	
~	~	Janet Prentice	
~	~	Julia Sampson	
~	~	Ann Schumacher	
~	~	Frances Shaw	
~	~	Michael Tarn	
~	~	James Whitehouse	
Birmingham, UK	City Hospital (Birmingham)	Emilio Porfiri	PI
~	~	Robert Stevenson	Co-I
~	~	Sachin Trivedi	Co-I
~	~	Laura Butler	
~	~	Yin May Chin	
~	~	Joanne Dasgin	
~	~	Debbie Devonport	
~	~	Daniel Ford	
~	~	Brian Gammon	
~	~	Harriet Goddard	
~	~	Jasbinder Kaur	
~	~	Alice Longe	
~	~	Amy Orme	
~	~	Lalit Pallan	
~	~	Steven Shanu	

INVESTIGATORS AND COLLABORATORS: SITE STAFF
Staff on site delegation logs

City	Care_Site	Person_Name	Site_PI
~	~	Julie Simpson	
~	~	Marion Tatman	
~	~	Angela Williams	
Birmingham, UK	Queen Elizabeth Hospital (Birmingham)	David Fackrell	PI
~	~	Nicholas James	Ex-PI
~	~	Daniel Ford	Co-I
~	~	Emilio Porfiri	Co-I
~	~	Kathryn Adams	
~	~	Salma Afzal	
~	~	Nicola Anderson	
~	~	Jay Ansari	
~	~	Biruk Asfaw	
~	~	Maria Bandeira	
~	~	Erica Beaumont	
~	~	Mahmoda Begum	
~	~	Shaleen Bishop	
~	~	Lea Booth	
~	~	Trish Brady	
~	~	Emma Bruce	
~	~	Laura Butler	
~	~	Laura Caley	
~	~	Helen Clarke	
~	~	Gemma Cole	
~	~	Jane Cook	
~	~	Amanda Davies	
~	~	Sara Diffley	
~	~	Claire Draycott	
~	~	Alison Grant	
~	~	Joanna Gray (nee Finney)	
~	~	Daniel Henderson	
~	~	Rosie Henvey	
~	~	Jenny Hiley	
~	~	Sharon Holmes	

INVESTIGATORS AND COLLABORATORS: SITE STAFF
Staff on site delegation logs

City	Care_Site	Person_Name	Site_PI
~	~	Sam Hopkins (nee Poole)	
~	~	Sameed Hussain	
~	~	Heather Jones	
~	~	Helen Jones	
~	~	Pamela Jones	
~	~	Alice Longe	
~	~	Daniella Lynch	
~	~	Fahd Niaz	
~	~	Andrew Palmer	
~	~	Stephanie Palmer	
~	~	Jenny Pascoe	
~	~	Zhane Peterkin	
~	~	Helen Preston	
~	~	Charlotte Sabine	
~	~	Rosemarie Seadon	
~	~	Amna Shah	
~	~	Tracy Soulsby	
~	~	Catherine Stead	
~	~	Lisa Thomas	
~	~	Syed Tirmazy	
~	~	Hannah Tolson	
~	~	Charlotte Trinham	
~	~	Arvind Tripathy	
~	~	Hannah Tween	
~	~	Vishy Veeranna	
~	~	Abel Zachariah	
~	~	Anjali Zarkar	
Blackburn, UK	Blackburn Royal Infirmary	Natalie Charnley	
Blackburn, UK	Royal Blackburn Hospital	Omi Parikh	PI
~	~	Zhu Oong	Co-I
~	~	Sophie Raby	Co-I
~	~	Danya Abdulwahid	
~	~	Ilyas Ahmed	

INVESTIGATORS AND COLLABORATORS: SITE STAFF
Staff on site delegation logs

City	Care_Site	Person_Name	Site_PI
~	~	Sarah Ainsworth	
~	~	Sue Ashworth	
~	~	Hazel Aston	
~	~	Ana Batista	
~	~	Karen Beard	
~	~	Gaynor Bowen	
~	~	Andrew Brocklehurst	
~	~	Fatima Butt	
~	~	Jackie Carey	
~	~	Naomi Charlton	
~	~	Helene Chorley	
~	~	Jenny Cockerill-Taylor	
~	~	Ruth Conroy	
~	~	Anthea Cree	
~	~	William Croxford	
~	~	Falalu Danwata	
~	~	Parth Desai	
~	~	Joseph Dykes	
~	~	Bethany Fielding	
~	~	Jan Flaherty	
~	~	Diane Forrest	
~	~	Helen Frankland	
~	~	James Grunshaw	
~	~	Samatha Guy	
~	~	Imran Haidar	
~	~	Hani Hanna	
~	~	Jeanette Hargreaves	
~	~	Kathryn Hayes	
~	~	Angela Hugill	
~	~	Andrew Hunnisett	
~	~	Rizwana Hussain	
~	~	Karen Jewers	
~	~	Sarah Keith	

INVESTIGATORS AND COLLABORATORS: SITE STAFF
Staff on site delegation logs

City	Care_Site	Person_Name	Site_PI
~	~	Prasad Kellati	
~	~	Tracey Kilduff	
~	~	Stephen Kilroy	
~	~	Jennifer King	
~	~	Andrew Lancaster	
~	~	Jasima Latif	
~	~	Matthew Lovell	
~	~	Jennifer McCallum	
~	~	Alexandra McCarrick	
~	~	Ajay Mehta	
~	~	Twesige Mugisa	
~	~	Tanmay Mukhopadhyay	
~	~	Jackie Nuttall	
~	~	Farzana Patel	
~	~	Karan Patel	
~	~	Graham Read	
~	~	Zia Rehman	
~	~	Karen Riley	
~	~	Christina Robinson	
~	~	Darren Rusk	
~	~	Janet Ryan-Smith	
~	~	Ahmed Salah	
~	~	Win Soe	
~	~	Helen Spickett	
~	~	Philippa Springle	
~	~	Dayle Squires	
~	~	Debbie Sutton	
~	~	Victoria Taylor	
~	~	Marianna Theodoulou	
~	~	Jacqueline Thomas	
~	~	Vivienne Tickle	
~	~	Richard Walshaw	
~	~	Lynsey Waring	

INVESTIGATORS AND COLLABORATORS: SITE STAFF

Staff on site delegation logs

City	Care_Site	Person_Name	Site_PI
~	~	Jessica Whiston	
~	~	Deborah Williamson	
~	~	Marcus Wise	
~	~	Maricica Zabrautanu	
Bolton, UK	Royal Bolton Hospital	Ling Lee	PI
~	~	Julie Chadwick	
~	~	Shirley Cocks	
~	~	Louise Dawson	
~	~	Tony Elliott	
~	~	Debbie Forkin	
~	~	Zoe Gall	
~	~	Robert Hull	
~	~	Collette Hunt	
~	~	Janine Hurst	
~	~	Karen Jewers	
~	~	Richard Jones	
~	~	Janet Keegan	
~	~	Karen Lee	
~	~	Charlotte Lever	
~	~	Ajay Mehta	
~	~	Raksha Mistry	
~	~	Gillian Mobb	
~	~	Michael Pantelides	
~	~	Hemant Patel	
~	~	Lindsay Rawlinson	
~	~	Sally Shaw	
Boston, UK	Pilgrim Hospital	Thiagarajan Sreenivasan	PI
~	~	Christian Arias	Co-I
~	~	David Ballesteros-Quintail	Co-I
~	~	Ana Fernandez-Ots	Co-I
~	~	Sekar (DV) Kittappa	Co-I
~	~	Miguel Panades	Co-I
~	~	Simon Archer	

INVESTIGATORS AND COLLABORATORS: SITE STAFF

Staff on site delegation logs

City	Care_Site	Person_Name	Site_PI
~	~	Giuseppe Banna	
~	~	Jayne Borley	
~	~	Eileen Busby	
~	~	Helen Carolan	
~	~	Prantik Das	
~	~	Jo Fletcher	
~	~	Andrew Judd	
~	~	Amy Kirkby	
~	~	Victoria Knight (n. Sherburn)	
~	~	Alice Latty	
~	~	Tara Lawrence nee Palmer	
~	~	Carol Lockwood	
~	~	Beverley Mashegede	
~	~	Karen Metcalf	
~	~	Sally Ann Molsher	
~	~	Kimberley Netherton	
~	~	Helen Palmer	
~	~	Kerry Pettitt	
~	~	Gunjan Phalod	
~	~	Sindhu Ramamurthy	
~	~	Amanda Roper	
~	~	Jenny Salmon	
~	~	Andrew Sloan	
~	~	Rebecca Spencer	
~	~	Kinga Szymiczek	
~	~	Isobel Thomas	
~	~	Laura Walsh	
~	~	Anita Young	
Bournemouth, UK	Royal Bournemouth Hospital	Sue Brock	PI
~	~	George Astras	
~	~	Natalya Boyd	
~	~	Eve Broadley	
~	~	David Chrastek	

INVESTIGATORS AND COLLABORATORS: SITE STAFF

Staff on site delegation logs

City	Care_Site	Person_Name	Site_PI
~	~	Joe Davies	
~	~	Deborah Hands	
~	~	Alison Hogan	
~	~	Lynsey Houlton	
~	~	Stephanie Jones	
~	~	Tiffany Joyce	
~	~	Katherine Major	
~	~	Rebecca Miln	
~	~	Nicky Naraine	
~	~	Natasha Ottley	
~	~	Kate Preece	
~	~	Laura Purandare	
~	~	Linda Purandare	
~	~	Cathie Purnell	
~	~	Taslima Rabbi	
~	~	Carlton Rowlands	
~	~	Sarah Savage	
~	~	Julie Thomson	
~	~	Luke Vamplew	
~	~	Rao Vuyyuru	
~	~	Min Wu	
Bradford, UK	Bradford Royal Infirmary	Simon Brown	PI
~	~	Michael Flatley	Co-I
~	~	Adel Jebar	Co-I
~	~	Lucy Jones	Co-I
~	~	Eldho Joseph	Co-I
~	~	Louise Karsera	Co-I
~	~	Sally Martin	Co-I
~	~	Sohail Mughal	Co-I
~	~	Lisa Owen	Co-I
~	~	Andrew Viggars	Co-I
~	~	Qamar Akbar	
~	~	Linda Bamford	

INVESTIGATORS AND COLLABORATORS: SITE STAFF
Staff on site delegation logs

City	Care_Site	Person_Name	Site_PI
~	~	Richard Benton	
~	~	Ian Boon	
~	~	Samuel Briggs	
~	~	Wendy Cardozo	
~	~	Sue Cheeseman	
~	~	Osman Chohan	
~	~	Ee Siang Choong	
~	~	Katy Clarke	
~	~	Kay Cockroft	
~	~	Victoria Drew	
~	~	Emma Dugdale	
~	~	Carol Firth	
~	~	Robina Ghulam	
~	~	Umair Hamid	
~	~	Catherine Handforth	
~	~	Ann Henry	
~	~	Hayley Inman	
~	~	Laura Jaques	
~	~	Ganesan Jeyasangar	
~	~	Charlotte Johnson-Smith	
~	~	Anne Marie Kay	
~	~	Lucille Kenyon	
~	~	Sophia Khan	
~	~	Leila Koudsi	
~	~	Jannika Lazarte	
~	~	Dan Lee	
~	~	Carmel Loughrey	
~	~	Reem Mahmood	
~	~	Leslie Masters	
~	~	Elizabeth McIntosh	
~	~	Dawn McNulty	
~	~	Chandran Nallathambi	
~	~	Gail Opio-Te	

INVESTIGATORS AND COLLABORATORS: SITE STAFF
Staff on site delegation logs

City	Care_Site	Person_Name	Site_PI
~	~	Shefali Parikh	
~	~	Mohammed Patel	
~	~	Charlotte Richardson	
~	~	Helen Robertshaw	
~	~	Sree Rodda	
~	~	Declan Ryan-Wakeling	
~	~	Jane Sewell	
~	~	Finbar Slevin	
~	~	Sophie Stephenson	
~	~	Kelvin Stewart	
~	~	Kim Storton	
~	~	Sarah Tinker	
~	~	Manitha Vinod	
~	~	Eleanor Waldron	
~	~	Lucy Ward	
~	~	Christopher Williams	
~	~	Helen Wilson	
~	~	You Yone	
~	~	Jamal Zekri	
~	~	Anthi Zeniou	
Bradford, UK	St Luke's (Bradford)	Susan Cheeseman	
Brighton, UK	Royal Sussex County Hospital	Angus Robinson	PI
~	~	George Plataniotis	Co-I
~	~	Dorota Bak-Blaz	
~	~	Lisa Barrott	
~	~	David Bloomfield	
~	~	Kirsty Bracewell	
~	~	Stephen Brown	
~	~	Maggie Cole	
~	~	Elizabeth Corbett	
~	~	Lucy Curtis	
~	~	George Devtsch	
~	~	Jane Dexter	

INVESTIGATORS AND COLLABORATORS: SITE STAFF

Staff on site delegation logs

City	Care_Site	Person_Name	Site_PI
~	~	Tarun Durga	
~	~	Rachel Rose Edmunds	
~	~	Emma Foreman	
~	~	Paul Frattaroli	
~	~	Lisa Furnival	
~	~	Jane Hanson	
~	~	Andrew Hart	
~	~	Daniel Henderson	
~	~	Samantha Hodges	
~	~	Catherine Hunter	
~	~	Summer Ibrahim	
~	~	Tamsin Kent	
~	~	Ranee Lactao	
~	~	Katie Langford	
~	~	Poppy Lavender	
~	~	Joanne Magennis	
~	~	Angela Man	
~	~	Pauline Martin	
~	~	Sebastien Martin	
~	~	Simon Matthews	
~	~	Helen Mitchell	
~	~	Amy Murray	
~	~	Monika Musiol	
~	~	Elaine Noon	
~	~	Annie Oliver	
~	~	Jane Peterson	
~	~	George Plantaniotis	
~	~	Alison Porges	
~	~	Tiago Rodrigues	
~	~	Tenesa Sargent	
~	~	Matthew Seal	
~	~	Victoria Sellick	
~	~	Jackie Sham	

INVESTIGATORS AND COLLABORATORS: SITE STAFF
Staff on site delegation logs

City	Care_Site	Person_Name	Site_PI
~	~	Jodie Smith	
~	~	Julie Smith	
~	~	Jean Tremlett	
~	~	Sue Trotter	
~	~	Vivien Tse	
~	~	Caroline Walker	
~	~	Karen Walker	
~	~	Chritianne Whitfield	
~	~	Marie Wilkins	
~	~	Bobbie Yoong	
Bristol, UK	Bristol Haematology & Oncology Centre	Amit Bahl	PI
~	~	Lloyd Abood	
~	~	Azeem Arshad	
~	~	Lindsay Ball	
~	~	Mark Beresford	
~	~	Sarah Bishop	
~	~	Jyothsna Chennupati	
~	~	Marc Coe	
~	~	Sibusiso Dhladhla	
~	~	Kay Drury	
~	~	Harvey Dymond	
~	~	Emily Foulstone	
~	~	Polly Gingell	
~	~	Tristan Grey	
~	~	Sally-Ann Hall	
~	~	Chris Herbert	
~	~	Serena Hilman	
~	~	Robert Hollister	
~	~	Amy Holloway	
~	~	Hayley Jones	
~	~	Stephen Lang	
~	~	Jayne Leonard	
~	~	Susan Masson	

INVESTIGATORS AND COLLABORATORS: SITE STAFF
Staff on site delegation logs

City	Care_Site	Person_Name	Site_PI
~	~	Shalini Mohan	
~	~	Hugh Newman	
~	~	Bryony Parrish	
~	~	Ian Penwarden	
~	~	Nick Robins	
~	~	Kimberly Rockley	
~	~	Helen Saldanha	
~	~	Sharon Short	
~	~	Beth Thorne	
~	~	Eve Watson	
~	~	Sandra Williams (nee Price)	
~	~	Paula Wilson	
~	~	Seonaid Wright	
Bristol, UK	Bristol Royal Infirmary	Lindsay Ball	
Burnley, UK	Burnley General Hospital	Omi Parikh	PI
~	~	Danya Abdulwahid	
~	~	Ilyas Ahmed	
~	~	Sarah Ainsworth	
~	~	Sue Ashworth	
~	~	Ana Batista	
~	~	Karen Beard	
~	~	Gaynor Bowen	
~	~	Andrew Brocklehurst	
~	~	Fatima Butt	
~	~	Jackie Carey	
~	~	Natalie Charnley	
~	~	Helene Chorley	
~	~	Ruth Conroy	
~	~	Anthea Cree	
~	~	Louise Dawson	
~	~	Bethany Fielding	
~	~	Jan Flaherty	
~	~	Diane Forrest	

INVESTIGATORS AND COLLABORATORS: SITE STAFF
Staff on site delegation logs

City	Care_Site	Person_Name	Site_PI
~	~	Helen Frankland	
~	~	Samatha Guy	
~	~	Imran Haidar	
~	~	Hani Hanna	
~	~	Jeanette Hargreaves	
~	~	Angela Hugill	
~	~	Rizwana Hussain	
~	~	Karen Jewers	
~	~	Sarah Keith	
~	~	Prasad Kellati	
~	~	Tracey Kilduff	
~	~	Stephen Kilroy	
~	~	Matthew Lovell	
~	~	Alexandra McCarrick	
~	~	Twesige Mugisa	
~	~	Tanmay Mukhopadhyay	
~	~	Farzana Patel	
~	~	Karan Patel	
~	~	Zia Rehman	
~	~	Karen Riley	
~	~	Christina Robinson	
~	~	Darren Rusk	
~	~	Janet Ryan-Smith	
~	~	Ahmed Salah	
~	~	Win Soe	
~	~	Helen Spickett	
~	~	Philippa Springle	
~	~	Dayle Squires	
~	~	Debbie Sutton	
~	~	Victoria Taylor	
~	~	Jacqueline Thomas	
~	~	Vivienne Tickle	
~	~	Richard Walshaw	

INVESTIGATORS AND COLLABORATORS: SITE STAFF
Staff on site delegation logs

City	Care_Site	Person_Name	Site_PI
~	~	Lynsey Waring	
~	~	Deborah Williamson	
~	~	Marcus Wise	
Burton-on-Trent, UK	Queen's Hospital Burton	Mike Smith-Howell	PI
~	~	Ann Adams	
~	~	Shahzad Ahmed	
~	~	Seheli Bandyopahdyay	
~	~	Gill Bell	
~	~	Jo Burns	
~	~	Lorraine Carter	
~	~	Prabir Chakraborti	
~	~	Shan Chetiyawardana	
~	~	Rosemary Corfield	
~	~	Helen Cox	
~	~	Helena Cox	
~	~	Chris Curtis	
~	~	Sudipta Datta	
~	~	Jacqueline Elliott	
~	~	Katy English (nee Parkes)	
~	~	Annette Fleet	
~	~	V Gajek	
~	~	Karzan Hama	
~	~	Sarah Hathaway-Lees	
~	~	Rajeev Kaushal	
~	~	Elizabeth Kemp	
~	~	Christopher Kent	
~	~	Ali Mahmmod	
~	~	Rohit Malde	
~	~	Chandrani Mallik	
~	~	Hanine Medani	
~	~	Clare Mewies	
~	~	Jennifer Moyes	
~	~	Dakshinamoorthy Muthukumar	

INVESTIGATORS AND COLLABORATORS: SITE STAFF
Staff on site delegation logs

City	Care_Site	Person_Name	Site_PI
~	~	Pugazhenthi Pattu	
~	~	Divya Ramadasan	
~	~	Anita Szita	
Bury St Edmunds, UK	West Suffolk Hospital	Cathryn Woodward	PI
~	~	Alex Martin	Co-I
~	~	Cherri Blades	
~	~	Gill Brett	
~	~	Deborah Clements-Dimmock	
~	~	James Curtis	
~	~	Elizabeth Devoy	
~	~	Yvonne Field	
~	~	Frances Flynn	
~	~	Susan Hale	
~	~	Mark Heath	
~	~	David Matter	
~	~	Tracey Murray	
~	~	Amanda Neal	
~	~	Lisa Patterson	
~	~	John Raja Ravendar	
~	~	Yvonne Rimmer	
~	~	Helen Small	
~	~	Jill Thain	
~	~	Fred Tuck	
Camarthen, UK	Glangwili General (formerly West Wales General)	Mau-Don Phan	PI
~	~	Sonya Goriah	Co-I
~	~	Samantha Coetzee	
~	~	Bleddyn Edwards	
~	~	Sandra Evens	
~	~	Ann Hewins	
~	~	Zohra Omar	
~	~	Bryan Phillips	
~	~	Meena Raj	
~	~	Rocio Riba	

INVESTIGATORS AND COLLABORATORS: SITE STAFF

Staff on site delegation logs

City	Care_Site	Person_Name	Site_PI
Cambridge, UK	Addenbrooke's Hospital	Danish Mazhar	PI
~	~	Tatiana Hernandez	Co-I
~	~	Rebecca Bradley	
~	~	Anita Chhabra	
~	~	Ellie Couch	
~	~	Gemma Cullen (née Godsall)	
~	~	Sandra Cunningham	
~	~	Mirela Hategan	
~	~	Carole Hewitt	
~	~	Luke Hughes-Davies	
~	~	Svitlana Iyevkova	
~	~	Gin Lee	
~	~	Rachel Lister	
~	~	Debra Mansergh	
~	~	Vanessa Moreira	
~	~	Isaac Opara	
~	~	Simon Pacey	
~	~	Glynn Rolland	
~	~	Matthew Stone	
~	~	Amy Strong n.Chandradass	
~	~	Andrew Styling	
~	~	James Tanner	
~	~	Safaa Therese	
~	~	Nicola Thompson	
~	~	Amanda Walker	
~	~	James Watson	
~	~	Han Wong	
~	~	Kamarul Zaki	
Canterbury, UK	Kent and Canterbury Hospital	Carys Thomas	PI
~	~	Patryk Brulinski	Co-I
~	~	Albert Edwards	Co-I
~	~	Joao Galante	Co-I
~	~	Jessica Gough	Co-I

INVESTIGATORS AND COLLABORATORS: SITE STAFF

Staff on site delegation logs

City	Care_Site	Person_Name	Site_PI
~	~	Jessica Little	Co-I
~	~	Natasha Mithal	Co-I
~	~	Rakesh Raman	Co-I
~	~	Alice Rendall	Co-I
~	~	Van Sim	Co-I
~	~	Ioannis Trigonis	Co-I
~	~	Jennifer Turner	Co-I
~	~	Ilyas Ahmed	
~	~	Louise Allen	
~	~	Bonny Appleby	
~	~	Sarah Beasley	
~	~	Sharon Beesley	
~	~	Hayley Blackgrove	
~	~	Tracy Boakes	
~	~	Julie Buckley	
~	~	Miguel Capo-Mir	
~	~	Natalie Catt	
~	~	Mathilda Cominos	
~	~	Denise Crawford	
~	~	Nikki Crisp	
~	~	Steve Dann	
~	~	Julie-Ann Davies	
~	~	Susan Drakeley	
~	~	Clary Evans	
~	~	Sam Gibson	
~	~	Andrew Gillian	
~	~	Louise Gladwell	
~	~	Coral Greenstreet	
~	~	Carolyn Hargreaves	
~	~	Gemma Hegarty	
~	~	Sandra Holness	
~	~	Laura Kehoe	
~	~	Sue Kelly	

INVESTIGATORS AND COLLABORATORS: SITE STAFF

Staff on site delegation logs

City	Care_Site	Person_Name	Site_PI
~	~	Rachel Larkins	
~	~	Kathryn Lees	
~	~	Sarah Lightfoot	
~	~	Sarah Lines	
~	~	Margaret Lipsham	
~	~	Diane Long	
~	~	Sydnie Loveland	
~	~	Rohit Malde	
~	~	Kim Mears	
~	~	Sharon Middleton	
~	~	Christos Mikropoulos	
~	~	Arafat Mirza	
~	~	Laura Mould	
~	~	Kannon Nathan	
~	~	Udaiveer Panwar	
~	~	Claire Pelham	
~	~	Karen Robinson	
~	~	Susan Rogers	
~	~	Lesley Rose	
~	~	Cindy Slater	
~	~	Mathini Sridharan	
~	~	Caroline Sunderland	
~	~	Stephane Tankoua	
~	~	Katy Taylor	
~	~	Kim Travis	
~	~	Alba Tubau	
~	~	Ifigenia Vasiliadou	
~	~	Kathleen (Kathy) Walsh	
~	~	Paula Whichelo	
~	~	Claire White	
~	~	Joanne Williams	
~	~	Elizabeth Williamson	
~	~	Victoria Williamson	

INVESTIGATORS AND COLLABORATORS: SITE STAFF
Staff on site delegation logs

City	Care_Site	Person_Name	Site_PI
~	~	Marian Wood	
~	~	Linda Wray	
~	~	Hilary Zurakovsky	
Cardiff, UK	University Hospital of Wales	Krishna Narahari	PI
~	~	Elizabeth Bois (nee Harris)	
~	~	Helen Clark	
~	~	Colette Clements	
~	~	Richard Coulthard	
~	~	Lynne Harry	
~	~	Samantha Holliday	
~	~	Clare Jones	
~	~	Howard Kynaston	
~	~	Kevin Pearse	
Cardiff, UK	Velindre Hospital	Jacob Tanguay	PI
~	~	Jim Barber	Co-I
~	~	Michael Button	Co-I
~	~	Aida Hanim Kamarudin	Co-I
~	~	Satish Kumar	Co-I
~	~	Malcolm Mason	Co-I
~	~	Nachiappan Palaniappan	Co-I
~	~	John Staffurth	Co-I
~	~	Kathy Bishop	
~	~	Clare Boobier	
~	~	Michael Brown	
~	~	Clair Brunner	
~	~	Lucy Chestney	
~	~	Helen Clark	
~	~	Lisa Victoria Jane Clayton	
~	~	Jessica Dermott (nee Platt)	
~	~	Clare Donnithorne	
~	~	Sarah Fry	
~	~	Sandra Greenslade	
~	~	Louise Harris	

INVESTIGATORS AND COLLABORATORS: SITE STAFF

Staff on site delegation logs

City	Care_Site	Person_Name	Site_PI
~	~	Nida Hassan	
~	~	Robert Henley	
~	~	Toby Hiscott	
~	~	Lynda Holman	
~	~	Gareth Hunt	
~	~	Amanda Jackson	
~	~	Rashmi Jadon	
~	~	Catherine John	
~	~	Alison Johnson	
~	~	Necia Jones	
~	~	Colette Kemp	
~	~	Lynette Lane	
~	~	Donna Lear	
~	~	Jason Lester	
~	~	Ross McLeish	
~	~	James Morgan	
~	~	Louise Morgan	
~	~	Phillip Morgan	
~	~	Diana Mort	
~	~	Debbie O'Connor	
~	~	Renata Poole	
~	~	Karen Pow	
~	~	Joanne Preece	
~	~	Leanne Quinn	
~	~	Tracy Rees	
~	~	Vicki Reynolds	
~	~	Cathy Richards	
~	~	Jayne Richards	
~	~	Emily Rumney	
~	~	Christian Smith	
~	~	Lisa Stafford	
~	~	Catherine Sullivan	
~	~	Loretta Sweeney	

INVESTIGATORS AND COLLABORATORS: SITE STAFF

Staff on site delegation logs

City	Care_Site	Person_Name	Site_PI
~	~	Hana Thomas	
~	~	Bethan Tranter	
~	~	Caroline Vitolo	
~	~	Lucy Wilbraham	
~	~	Gillian Willetts	
~	~	Kay Wilson	
~	~	Charlotte Young	
Carlisle, UK	Cumberland Infirmary	Fiona Douglas	PI
~	~	Anil Kumar	PI
~	~	Angela Birt	
~	~	Christopher Brewer	
~	~	Diane Donnelly	
~	~	Charlotte Eyles	
~	~	Grace Fryer	
~	~	Ivor Hughes	
~	~	Patricia Nicholls	
~	~	Jonathan Nicoll	
~	~	Muhammad Rahman	
~	~	Norma Sidek	
~	~	Jenna Wildey	
~	~	Beverley Wilkinson	
~	~	Fergus Young	
Chelmsford, UK	Broomfield Hospital	Abdel Hamid	PI
~	~	Gopalakrishnan Srinivasan	Co-I
~	~	Victoria Apps	
~	~	Christian Barnett	
~	~	Melanie Boxall	
~	~	Donna Briggs	
~	~	Frances Cairns	
~	~	Tracey Camburn	
~	~	Emma Cannon	
~	~	Jennifer Child	
~	~	Lucy Cooper	

INVESTIGATORS AND COLLABORATORS: SITE STAFF
Staff on site delegation logs

City	Care_Site	Person_Name	Site_PI
~	~	Elizabeth Dawson	
~	~	Sarah Ferguson	
~	~	Sian Gibson	
~	~	Jane Giles	
~	~	Dane Goodere-Bennett	
~	~	Kiran Kancherla	
~	~	Priscilla Leone	
~	~	Yvonne Lester	
~	~	Isabella Maund	
~	~	Emma Mitchell	
~	~	Udaiveer Panwar	
~	~	Enca Parsons	
~	~	Melanie Ruben	
~	~	Victoria Scott	
~	~	Bryan Singizi	
~	~	Edel Spruce	
~	~	Amon Wijunamai	
~	~	Lucy Willsher	
~	~	You Yone	
Cheltenham, UK	Cheltenham General Hospital	Jo Bowen	PI
~	~	Peter Jenkins	Co-I
~	~	Julie Allen	
~	~	Susan Anderson	
~	~	Charlotte Ayrton	
~	~	Helen Babbage	
~	~	Rehana Bakawala	
~	~	Sarah Beazer	
~	~	Victoria Bell	
~	~	Vishal Bhalla	
~	~	Lucy Blake	
~	~	Caitlin Bowden	
~	~	Rachel Carter	
~	~	Bethan Cartwright	

INVESTIGATORS AND COLLABORATORS: SITE STAFF

Staff on site delegation logs

City	Care_Site	Person_Name	Site_PI
~	~	Jyothsna Chennupati	
~	~	Jill Chittock	
~	~	Audrey Cook	
~	~	Samuel Croly	
~	~	Lin Crossley	
~	~	Jennifer Dewett	
~	~	Rachel Durrant	
~	~	Chris Ford	
~	~	Janet Forkes	
~	~	Julia Hall	
~	~	Jennifer Healey-Mariano	
~	~	Ian Ingledew	
~	~	Sai Jonnada	
~	~	Louise Kidner	
~	~	Laura Malins	
~	~	Rebecca Mesher	
~	~	Roger Owen	
~	~	Elisabeth Read	
~	~	Rachel Sayers	
~	~	Elaine Sizer	
~	~	Amy Skelton	
~	~	Jennifer Smith	
~	~	Sarah Stanley	
~	~	Duncan Stow	
~	~	Abi Stuart	
~	~	Catherine Stuart-Grumbar	
~	~	Matthew Tan	
~	~	Kate Trigg-Hogarth	
~	~	Richard Wallis	
~	~	Alex Williams	
~	~	Sue Wronski	
Chester, UK	Countess of Chester Hospital	Azman Ibrahim	PI
~	~	Mary Aldous	

INVESTIGATORS AND COLLABORATORS: SITE STAFF
Staff on site delegation logs

City	Care_Site	Person_Name	Site_PI
~	~	Ian Allen	
~	~	Denise Archer	
~	~	Wesley Artist	
~	~	Emma Barry	
~	~	Lucy Beresford	
~	~	Kathryn Cawley	
~	~	Lisa Dobson (nee Child)	
~	~	Helen Eccleson	
~	~	Chelcie Faulkner	
~	~	Elizabeth Gallimore	
~	~	Sue Green	
~	~	Rebecca Grogan	
~	~	Jenny Grounds	
~	~	Rebecca Hopcroft	
~	~	Sarah Illingworth	
~	~	Helen Elizabeth Jeffrey	
~	~	Grace McGrath	
~	~	Jenny Miller	
~	~	Judith Prince	
~	~	Shannon Spicer	
~	~	Janet Spriggs	
~	~	Joshua Williams	
Colchester, UK	Colchester General Hospital	Dakshinamoorthy Muthukumar	PI
~	~	Devy Basu	Co-I
~	~	Rana Mahmood	Co-I
~	~	Bruce Sizer	Co-I
~	~	Anita Szita	Co-I
~	~	Katrina Cooke	
~	~	Nicola Cutmore	
~	~	Celine Driscoll	
~	~	Michelle Fisher	
~	~	Richard Gant	
~	~	Hayley Hewer	

INVESTIGATORS AND COLLABORATORS: SITE STAFF

Staff on site delegation logs

City	Care_Site	Person_Name	Site_PI
~	~	Liz Hunting	
~	~	Jane Ketley-O'Donel	
~	~	Muthar Kumar	
~	~	Louies Mabelin	
~	~	Michelle Marshall	
~	~	Sunil Skaria	
~	~	Daisuke Takeuchi	
~	~	Lucy Thorogood	
Colchester, UK	Essex County Hospital	Devy Basu	
~	~	Lorna Dewar	
~	~	Celine Driscoll	
~	~	Hayley Hewer	
~	~	Liz Hunting	
~	~	Jane Ketley-O'Donel	
~	~	Muthar Kumar	
~	~	Michelle Marshall	
~	~	Pugazhenthi Pattu	
~	~	Bruce Sizer	
~	~	Lucy Thorogood	
Cottingham, UK	Castle Hill Hospital	Matthew Simms	PI
~	~	Faheem Bashir	Co-I
~	~	Mohammad Butt	Co-I
~	~	Mohan Hingorani	Co-I
~	~	Mateen Akhtar	
~	~	Ian Beckley	
~	~	Linzi Bone	
~	~	George Bozat	
~	~	Sarah Brown	
~	~	Suzy Bunton	
~	~	Bob Bush	
~	~	Mary Garthwaite	
~	~	Jonathan Gill	
~	~	John Hetherington	

INVESTIGATORS AND COLLABORATORS: SITE STAFF
Staff on site delegation logs

City	Care_Site	Person_Name	Site_PI
~	~	Carol Hodson	
~	~	Linda Hoggarth	
~	~	Louise Karsera	
~	~	Vicki Lowthorpe	
~	~	Jenny Marsden	
~	~	Sarah Moffat	
~	~	Iqtedar Muazzam	
~	~	Paula O'Reilly	
~	~	Sarah Palmer	
~	~	Kristian Plowman	
~	~	Dulani Ranatunge	
~	~	Julie Rawlings	
~	~	Lucy Richardson	
~	~	Karen Stubbs	
~	~	Adam Wolstencroft	
~	~	A Yousuff	
~	~	Khawaje Zahid	
Coventry, UK	Coventry and Warwickshire Hospital	Leila Fortunato	
Coventry, UK	University Hospital Coventry and Warwickshire	Jane Worlding	PI
~	~	Joanna Hamilton	Co-I
~	~	Shah Rafique	Co-I
~	~	Rebecca Aaron	
~	~	Jason Allen	
~	~	Senthil Kumar Athmanathan	
~	~	Rachel Bazeley	
~	~	Maggie Brown	
~	~	Vikki Browne	
~	~	Dannielle Burgess	
~	~	Luanne Carey	
~	~	Andrew Chan	
~	~	Rajbinder Deol	
~	~	Theresa Griffiths	
~	~	Kieran Jefferson	

INVESTIGATORS AND COLLABORATORS: SITE STAFF

Staff on site delegation logs

City	Care_Site	Person_Name	Site_PI
~	~	Mohammed Khan	
~	~	Yakhub Khan	
~	~	Donald Macdonald	
~	~	Fiona McGurk	
~	~	Lucy Miller	
~	~	Albert Mislang	
~	~	Mohamed Mooradun	
~	~	Su Ngwenya	
~	~	Zoe O'Neill	
~	~	Sarah O'Toole	
~	~	Karandeepu Pachoo	
~	~	Sonia Powell	
~	~	Sue Robinson	
~	~	Sukhbinder Salh	
~	~	Noor Ayesha Shah	
~	~	Elaine Simmons	
~	~	Laura Stanley	
~	~	Andrew Stockdale	
~	~	Vicky Sturgess	
~	~	Charlie-marie Suddens	
~	~	Rachel Thompson	
~	~	Fiona Tranter	
~	~	Jenny Warmington	
~	~	Mark Whitmore	
~	~	Linda Wimbush	
Crewe, UK	Leighton Hospital	Anna Tran	PI
~	~	Vanessa Adamson	
~	~	Carole Bennion	
~	~	Kim Best	
~	~	Michael Braun	
~	~	David Butterworth	
~	~	Lydia Buxton	
~	~	Osman Chohan	

INVESTIGATORS AND COLLABORATORS: SITE STAFF

Staff on site delegation logs

City	Care_Site	Person_Name	Site_PI
~	~	William Croxford	
~	~	Thiraviyam Elumalai	
~	~	Leanne Everall	
~	~	Julia Gemmell	
~	~	Sarah Hoswell	
~	~	Adele Hough	
~	~	Chris Hough	
~	~	P Irwin	
~	~	P Javle	
~	~	Taya Jones	
~	~	Tracy Larcombe	
~	~	Carolyn Mansfield	
~	~	Emma Margerum	
~	~	Julie Meir	
~	~	Gemma Nash	
~	~	Andrew Ritchings	
~	~	Rachel Smith	
~	~	Catherine Thompson	
~	~	Sarah Tinsley	
~	~	Caroline Walker	
~	~	James Wylie	
Croydon, UK	Croydon University Hospital	Cheryl Batish	
~	~	Yvonne Campbell	
~	~	Anne Haldeos	
~	~	Ann Payne	
~	~	Jane Thomson	
Darlington, UK	Darlington Memorial Hospital	Mohammed Kagzi	PI
~	~	Rachel Chatt	
~	~	Alison Chilvers	
~	~	Penny Gamble	
~	~	Helen Haley	
~	~	John Hardman	
~	~	Claire Henderson	

INVESTIGATORS AND COLLABORATORS: SITE STAFF

Staff on site delegation logs

City	Care_Site	Person_Name	Site_PI
~	~	Hyder Latif	
~	~	Julia McBride	
~	~	Lorna Morgan	
~	~	Tanmay Mukhopadhyay	
~	~	Richard Nendick	
~	~	Clive Peedell	
~	~	Calum Polwart	
~	~	Steven Pratt	
~	~	Asia Sarwar	
~	~	Jane Shaw	
~	~	Kimberly Stamp	
~	~	Lynsey Stephenson	
~	~	Jonathan Stoddard	
~	~	Fiona Strong	
~	~	John Vickers	
~	~	Susan Wadd	
Dartford, UK	Darent Valley Hospital	Louise Lacey	
Derby, UK	London Road Community Hospital	Kay Bowdler	
~	~	Prabir Chakraborti	
~	~	Debbie Davis	
~	~	Kristina Duggleby	
~	~	Sarah Hare	
~	~	Sarah Hathaway-Lees	
~	~	Heini Jussila	
~	~	Jane Lawrie	
~	~	Wendy Morrisroe	
~	~	Dakshinamoorthy Muthukumar	
~	~	Karen Simmonds	
~	~	Keeley Smith	
~	~	Colin Ward	
Derby, UK	Royal Derby Hospital	Prantik Das	PI
~	~	Wendy Abbott	
~	~	Shahzad Ahmed	

INVESTIGATORS AND COLLABORATORS: SITE STAFF
Staff on site delegation logs

City	Care_Site	Person_Name	Site_PI
~	~	James Aldous	
~	~	Donna Beal	
~	~	Elizabeth Bedford	
~	~	Liz Bedford	
~	~	Helen Beveridge	
~	~	Sathan Boonyaprapa	
~	~	Sonya Bradshaw	
~	~	Louise Brookes	
~	~	Alison Carrick	
~	~	Prabir Chakraborti	
~	~	Josephine Chmiel	
~	~	Caroline Coulson	
~	~	Kiran Das	
~	~	Julie Dockree	
~	~	Charlotte Downes	
~	~	Julie Edmonds	
~	~	Jodie Fitzgerald	
~	~	Aaron Gallagher	
~	~	Marie Ann Goldsworthy	
~	~	Sarah Hare	
~	~	Margaret Harper	
~	~	Gemma Irvine	
~	~	Christopher Kent	
~	~	Sarah Longhurst	
~	~	Fanuel Magaya	
~	~	Peter Mason	
~	~	Alastair McCabe	
~	~	Lucy McCandless	
~	~	Lorraine McDonald	
~	~	Nicole McKee	
~	~	Nicole McKee (nee Isitt)	
~	~	Jennifer Mitchell	
~	~	Wendy Morrisroe	

INVESTIGATORS AND COLLABORATORS: SITE STAFF
Staff on site delegation logs

City	Care_Site	Person_Name	Site_PI
~	~	Thangarajah Mugunthan	
~	~	Dakshinamoorthy Muthukumar	
~	~	Elizabeth Nadin	
~	~	Ajith Gopinathan Nair	
~	~	Pugazhenthi Pattu	
~	~	Ellie Piggott	
~	~	Timothy Podd	
~	~	Ayman Ramadan	
~	~	Gemma Redfern	
~	~	Manni Sandhu	
~	~	Karen Simmonds	
~	~	Virgil Sivoglo	
~	~	Kashmira Subramanian	
~	~	Sarah Taylor	
~	~	Janet Tomlinson	
~	~	Colin Ward	
~	~	Claire Wintle	
~	~	Chris Worth	
~	~	Georgia Wright	
Doncaster, UK	Doncaster Royal Infirmary	Carmel Pezaro	PI
~	~	Virgil Sivoglo	Ex-PI
~	~	Lucy Smith	Co-I
~	~	Jessica Tay	Co-I
~	~	Sharon Ann Allen	
~	~	Mymoona Alzouebi	
~	~	Sarah Brown	
~	~	Barbara Burlace	
~	~	Robert Chadwick	
~	~	Rachel Codling	
~	~	Joanne Derx	
~	~	Ben East	
~	~	Laura Ellis	
~	~	Catherine Ferguson	

INVESTIGATORS AND COLLABORATORS: SITE STAFF
Staff on site delegation logs

City	Care_Site	Person_Name	Site_PI
~	~	Janet Field	
~	~	Alexandra Firth	
~	~	Meredyth Harris	
~	~	Mark Holliday	
~	~	Nicole Jeffcutt	
~	~	Joanne McNally	
~	~	Amy Neal	
~	~	Muneeb Qureshi	
~	~	Janine Smedley (nee McCabe)	
~	~	Jennifer Taylor	
~	~	Deborah Walstow	
~	~	Lisa Warren	
~	~	Nicola Wilkinson	
~	~	Kim Wood	
Dorchester, UK	Dorset County Hospital	Benjamin Masters	PI
~	~	Naveed Afzal	
~	~	Beverley Anderson	
~	~	Stephen Andrews	
~	~	Pauline Ashcroft	
~	~	Piet Bakker	
~	~	Lynn Billett	
~	~	Robert Blegay	
~	~	Laura Bough	
~	~	Sally Breakspear	
~	~	Susan Carr	
~	~	Ananda Chakrabarti	
~	~	Andrew Cornaby	
~	~	Perric Crellin	
~	~	Andrew Gibbins	
~	~	Jackie Gibbins	
~	~	Tracy Glen	
~	~	Josie Goodsell	
~	~	Sarah Horton	

INVESTIGATORS AND COLLABORATORS: SITE STAFF

Staff on site delegation logs

City	Care_Site	Person_Name	Site_PI
~	~	Stephanie Jones	
~	~	Sally Love	
~	~	Louise O'Shea	
~	~	Andrew Rees	
~	~	Simon Sharpe	
~	~	Delia Whiteman	
~	~	Suzy Wignall	
~	~	Sarah Williams	
Dudley, UK	Russells Hall Hospital	Pek Keng-Koh	PI
~	~	Mano Joseph	Co-I
~	~	Joseph Mano	Co-I
~	~	Paul Anderson	
~	~	Joann Atkinson	
~	~	David Edwards	
~	~	Lesley Edwards	
~	~	Lawrence Emtage	
~	~	Irene Gardner	
~	~	Georgi Georgiev	
~	~	Dee Harris	
~	~	Kath Harrow	
~	~	Nadira Jilani	
~	~	Ruckie Kahlon	
~	~	Jayne Kanwar	
~	~	Karen Kanyi	
~	~	Sally Keates-Porter	
~	~	Julie Matthews	
~	~	Heather McClure	
~	~	Emily McDonald	
~	~	Karen McGarry	
~	~	Vanessa Moore	
~	~	Andrew Moores	
~	~	Jenny O'Grady	
~	~	Manesh Patel	

INVESTIGATORS AND COLLABORATORS: SITE STAFF
Staff on site delegation logs

City	Care_Site	Person_Name	Site_PI
~	~	Hayley Pearson	
~	~	Karen Pearson	
~	~	Prakash Ramachandra	
~	~	Ellen Shirley	
~	~	Lucy Smith	
~	~	Sara Smith	
~	~	Lucie Smith (nee Williams)	
~	~	Anna Summerfield	
~	~	Syed Tirmazy	
~	~	Ellie Traverse	
~	~	Angela Watts	
~	~	Abel Zachariah	
Dundee, UK	Ninewells Hospital	Sangeetha Ponnusamy	
Durham, UK	University Hospital of North Durham	Jean Dent	
~	~	Julie Elliot	
~	~	Julie Elliott	
~	~	Jeanette Maughan	
~	~	Rhona McMenemin	
~	~	Lorna Morgan	
~	~	Andrew Parker	
~	~	Dorothy Turnbull	
~	~	Sarah Welsh	
Eastbourne, UK	Eastbourne District General Hospital	Caroline Manetta	PI
~	~	Shelley Baumber	
~	~	Theresa Baumber	
~	~	Duncan Gilbert	
~	~	Prudence Hobbs	
~	~	Joanna Howard	
~	~	Kay Jones-Skipper	
~	~	William Lawrence	
~	~	Lauren McCrisken	
~	~	Fiona McKinna	
~	~	Peter Rimington	

INVESTIGATORS AND COLLABORATORS: SITE STAFF

Staff on site delegation logs

City	Care_Site	Person_Name	Site_PI
~	~	Neville Sharma	
~	~	David Sharp	
~	~	Aspasia Soultati	
~	~	Graham Watson	
~	~	Mark Whitfield	
Edinburgh, UK	Western General Hospital	Duncan McLaren	PI
~	~	Alistair Law	Co-I
~	~	Jahangeer Malik	Co-I
~	~	Richard Allan	
~	~	Claire Arthur	
~	~	Jennifer Baxter	
~	~	Prasad Bollina	
~	~	Tracy Brear	
~	~	Ewan Brown	
~	~	Caroline Bruce	
~	~	Alison Clark	
~	~	Ann Cochrane	
~	~	Heather Dalrymple	
~	~	Martin Doak	
~	~	Roland Donat	
~	~	Lisa Egan	
~	~	Ben Elliott	
~	~	Olvsola Faluyi	
~	~	Susan Forman	
~	~	Fiona Gardiner	
~	~	Nikki Gilluley	
~	~	Lynn Ho	
~	~	Grahame Howard	
~	~	Heather Howie	
~	~	David Jeffrey	
~	~	Emma Lewis	
~	~	Ailsa Liddle	
~	~	Hannah Lord	

INVESTIGATORS AND COLLABORATORS: SITE STAFF
Staff on site delegation logs

City	Care_Site	Person_Name	Site_PI
~	~	Sanjana Masinghe	
~	~	Barbara Mayne	
~	~	John McGrane	
~	~	Alison McKinlay	
~	~	Alan McNeill	
~	~	Heather McVicars	
~	~	Hazel Milligan	
~	~	Beverley Mitchell	
~	~	Kirsty Peebles	
~	~	Lois Pollock	
~	~	Brian Rogers	
~	~	Fionagh Ross	
~	~	Theresa Savage	
~	~	Andrea Stanton	
~	~	Mark Stares	
~	~	Sarah Thompson	
~	~	David Tulloch	
~	~	Vivienne Wilson	
~	~	Katie Wood	
~	~	Catherine Woods	
Edmonton, UK	North Middlesex Hospital	Nishi Gupta	PI
~	~	Chris Abbott	
~	~	Beatrice Balachandran	
~	~	Girish Bhome	
~	~	Debbie Blois	
~	~	Tom Caumont	
~	~	Bernadette Collins	
~	~	Judy Hill	
~	~	Lorraine Hurl	
~	~	Stephen Karp	
~	~	Ursula McGovern	
~	~	Lucinda Melcher	
~	~	Farhad Neave	

INVESTIGATORS AND COLLABORATORS: SITE STAFF
Staff on site delegation logs

City	Care_Site	Person_Name	Site_PI
~	~	Jackie Newby	
~	~	Kathy O'Farrell	
~	~	Asim Ray	
~	~	Kerri Rees	
~	~	Mausam Singhera	
~	~	Ferrial Syed	
~	~	Anna Thompson	
~	~	Chloe Van Someren	
Exeter, UK	Royal Devon and Exeter Hospital	Denise Sheehan	PI
~	~	San Aung	Co-I
~	~	Rajaguru Srinivasan	Co-I
~	~	Peter Stephens	Co-I
~	~	John Anderson	
~	~	Alison Augstburger	
~	~	Kizzy Baines	
~	~	Alan Betts	
~	~	David Jonathan Chambers	
~	~	Tamika Chapter	
~	~	Ross Curwen	
~	~	Susan Davenport	
~	~	Elizabeth Davey	
~	~	Melissa Davey	
~	~	Susan Downer	
~	~	Dawn Edwards	
~	~	Stephanie Ann Ellis	
~	~	Victoria Ford	
~	~	Tracey Foss	
~	~	Emma Guerin	
~	~	Anne Hong	
~	~	Frances Hood	
~	~	Beverley Kemp	
~	~	Theresa Lawless	
~	~	James Leavy	

INVESTIGATORS AND COLLABORATORS: SITE STAFF
Staff on site delegation logs

City	Care_Site	Person_Name	Site_PI
~	~	Christoph Lohan	
~	~	Anna Lydon	
~	~	Lyndel Moore	
~	~	Ayman Nassar	
~	~	Tim Norris	
~	~	Kate O'Connor	
~	~	Jane Piper	
~	~	Claire Ridler	
~	~	Alison Roantree	
~	~	Emma Robjohns	
~	~	Ingrid Seath	
~	~	Suzy Tasker	
~	~	Shirley Todd	
~	~	Elizabeth Toy	
~	~	Matt Trivett	
~	~	Elaine Vandcandelaere	
~	~	Fiona Walters (nee Hall)	
~	~	Sophie Warren	
~	~	Claire Webb	
Gillingham, UK	Medway Maritime Hospital	Stergios Boussios	PI
~	~	Henry Taylor	Ex-PI
~	~	Charlotte Abson	Co-I
~	~	Christos Mikropoulos	Co-I
~	~	Khalid Abdalla	
~	~	Philip Adeniran	
~	~	Diletta Bianchini	
~	~	Louise Black	
~	~	Corinne Borley	
~	~	Louise Brassington	
~	~	Deirdre Cooke	
~	~	Parool Darbar	
~	~	Charles Davis	
~	~	Tamara Diamond	

INVESTIGATORS AND COLLABORATORS: SITE STAFF
Staff on site delegation logs

City	Care_Site	Person_Name	Site_PI
~	~	Mary Everett	
~	~	Durga Maya Gurung	
~	~	Marie Louise Hollands	
~	~	Kay Jones	
~	~	Afroditi Karathanasi	
~	~	Tessa Lawrence	
~	~	Carol Mayger	
~	~	Peter Milverton	
~	~	Kevin Naicker	
~	~	Elizabeth Newman-Horne	
~	~	Lisa Parker	
~	~	Suzie Reyner	
~	~	Alison Richards	
~	~	Agne Sadauskaite	
~	~	James Sawyer	
~	~	Jodie Seymour	
~	~	Nicola Southwell	
~	~	Emma Sutton	
~	~	Swapna Thomas	
~	~	Richard Thornton	
~	~	Alba Tuban	
~	~	Katarzyna Urbanczyk	
~	~	Gayzel Vallejera	
~	~	Simon Wan	
Glasgow, UK	Beatson West of Scotland Cancer Centre	Rob Jones	PI
~	~	John Graham	Ex-PI
~	~	Kathryn Banfill	Co-I
~	~	Derek Grose	Co-I
~	~	Carolynn Lamb	Co-I
~	~	Tareq Abdullah	
~	~	Abdulla Al-hasso	
~	~	Mohammed Alfayez	
~	~	Jawaher Ansari	

INVESTIGATORS AND COLLABORATORS: SITE STAFF
Staff on site delegation logs

City	Care_Site	Person_Name	Site_PI
~	~	Miranda Ashton	
~	~	Patricia Baird	
~	~	Martin Ball	
~	~	Gillian Barmack	
~	~	Sophie Barrett	
~	~	Lorraine Barwell	
~	~	Karen Bell	
~	~	Jenny Brown	
~	~	Louise Bruce	
~	~	Nicola Cairns	
~	~	Ross Carruthers	
~	~	Almudena Cascales	
~	~	Annette Charlick	
~	~	Maureen Connolly	
~	~	Catriona Cowan	
~	~	Alice Coy	
~	~	Cicely Cunningham	
~	~	Judith Dixon	
~	~	David Dodds	
~	~	Gerard Forrest	
~	~	Ben Fulton	
~	~	Katie Galbraith	
~	~	Hilary Glen	
~	~	Jacqueline Gourlay	
~	~	Jan Graham	
~	~	Janet Graham	
~	~	Kathryn Graham	
~	~	Lynne Grieve	
~	~	Ailsa Griffen	
~	~	Sally Hall	
~	~	Maureen Hamill	
~	~	Maryon Hardie	
~	~	Paula Henry-Stephenson	

INVESTIGATORS AND COLLABORATORS: SITE STAFF
Staff on site delegation logs

City	Care_Site	Person_Name	Site_PI
~	~	Awris Jalil	
~	~	Sai Juan Jia	
~	~	Gemma Johnson	
~	~	Saranya Kakumanu	
~	~	Ashleigh Kerr	
~	~	Esfandiyar Khan	
~	~	Kirsten Laws (nee Borthwick)	
~	~	Graeme Lumsden	
~	~	Antonia MacMillan	
~	~	Nicholas Macleod	
~	~	Rana Mahmood	
~	~	Husam Marashi	
~	~	Brendan McCann	
~	~	Stephen McKay	
~	~	Fiona McQueen	
~	~	Rebecca Muirhead	
~	~	Maria Nicol	
~	~	Stefan Nowicki	
~	~	Ruth Orr	
~	~	Aqilah Othman	
~	~	Jennifer Petrie	
~	~	Linzi Rae	
~	~	Nathan Richardson	
~	~	Patricia Roxburgh	
~	~	Martin Russell	
~	~	Azmat Sadozye	
~	~	Ian Sanders	
~	~	Norma Sidek	
~	~	Claire Steele	
~	~	Kirsteen Stuart	
~	~	Diann Taggart	
~	~	Lye Mun Tho	
~	~	Aisha Tufail	

INVESTIGATORS AND COLLABORATORS: SITE STAFF

Staff on site delegation logs

City	Care_Site	Person_Name	Site_PI
~	~	Balaji Venugopal	
~	~	Jan Wallace	
~	~	Hannah Weir	
~	~	Christina Wilson	
Gloucester, UK	Gloucestershire Royal Hospital	Jo Bowen	PI
~	~	Peter Jenkins	Co-I
~	~	Julie Allen	
~	~	Charlotte Ayrton	
~	~	Sarah Beazer	
~	~	Victoria Bell	
~	~	Bethan Cartwright	
~	~	Audrey Cook	
~	~	Samuel Croly	
~	~	Lin Crossley	
~	~	Chris Ford	
~	~	Janet Forkes	
~	~	Julia Hall	
~	~	Sai Jonnada	
~	~	Laura Malins	
~	~	Sarah Matthews	
~	~	Louise Moore	
~	~	Roger Owen	
~	~	Elisabeth Read	
~	~	Claire Salter	
~	~	Rachel Sayers	
~	~	Elaine Sizer	
~	~	Amy Skelton	
~	~	Sarah Stanley	
~	~	Abi Stuart	
~	~	Kate Trigg-Hogarth	
~	~	Richard Wallis	
~	~	Sue Wronski	
Guildford, UK	Royal Surrey County Hospital	Carla Perna	PI

INVESTIGATORS AND COLLABORATORS: SITE STAFF

Staff on site delegation logs

City	Care_Site	Person_Name	Site_PI
~	~	Leslie Cheng	Co-I
~	~	Mahwish Karim	Co-I
~	~	Richmond Abeseabe	
~	~	Kavita Bhat	
~	~	Caterina Bissa	
~	~	Melanie Boafo-Yirenkyi	
~	~	Fiona Butler	
~	~	Marianne Dabbs	
~	~	Veronica Davis	
~	~	Sarah De Swert	
~	~	Maria Drzymala	
~	~	Daisy Floyd	
~	~	Teresa Guerrero-Urbano	
~	~	Lesley Harden	
~	~	Celia Harris	
~	~	Imogen Heenan	
~	~	Adele Hugg	
~	~	Stephy Joseph	
~	~	Jen Julius	
~	~	Teresa Keating	
~	~	Sara Khaksar	
~	~	Zephyrine King	
~	~	Robert Laing	
~	~	Emmanuel Larbi	
~	~	James Lowe	
~	~	Catherine Medcalf	
~	~	Julian Money-Kyrle	
~	~	Mahomed Moosa	
~	~	Angela Morgan	
~	~	Linda Nardone	
~	~	Kathrin Narvaez-Vega	
~	~	Jenny Nobes	
~	~	Kate Penhaligon	

INVESTIGATORS AND COLLABORATORS: SITE STAFF
Staff on site delegation logs

City	Care_Site	Person_Name	Site_PI
~	~	Nick Pilkington	
~	~	Sue Sargent	
~	~	Richard Shaffer	
~	~	Charlotte Shelley	
~	~	Frances Sidi	
~	~	Joanna Stokoe	
~	~	Sree Susaria	
~	~	Miriam White	
~	~	Julia Whittle	
~	~	Katie Wood	
~	~	Jane Woods	
Harlow, UK	Princess Alexandra Hospital (Harlow)	Lucinda Melcher	PI
~	~	Tasia Aghadiuno	Co-I
~	~	Nishi Gupta	Co-I
~	~	Anna Lerner	Co-I
~	~	Hamoun Rozati	Co-I
~	~	Zainab Wasim	Co-I
~	~	Gemma Cook	
~	~	Amelia Daniel	
~	~	Reena Davda	
~	~	Shroma De Silva	
~	~	Albert Edwards	
~	~	Sunjalee Fernando	
~	~	Ahmed Hnoosh	
~	~	Evelyn Holmes	
~	~	Jodie Johnson	
~	~	Paul Kabuubi	
~	~	Joanne Kellaway	
~	~	Amanda Lewis	
~	~	Amy Lewis	
~	~	Teresa Light	
~	~	Cait Rees	
~	~	Ervin Shpuza	

INVESTIGATORS AND COLLABORATORS: SITE STAFF
Staff on site delegation logs

City	Care_Site	Person_Name	Site_PI
~	~	Tracey White	
~	~	Nikki White (nee Staines)	
Haverford West, UK	Withybush General Hospital	Sandra Evens	
Hereford, UK	Hereford County Hospital	Warren Grant	PI
~	~	Susan Anderson	
~	~	Anita Ashton	
~	~	Vishal Bhalla	
~	~	Caitlin Bowden	
~	~	Sophie Boyd	
~	~	Sophie Boyd (nee Evans)	
~	~	Serrafina Carini	
~	~	Jagdish Chana	
~	~	Audrey Cook	
~	~	Sophie Cooper	
~	~	Melanie Evans	
~	~	Maxine Flubacher	
~	~	Janet Forkes	
~	~	Kate Hammerton	
~	~	Andy Hedges	
~	~	Gill Horsfield	
~	~	Jenny Howls	
~	~	Claire Hughes	
~	~	Janine Jones (Birch)	
~	~	Lisa King	
~	~	Laura Lees	
~	~	Rachel Lowe	
~	~	Linda Moseley	
~	~	Naeem Musani	
~	~	Jolanta Pueskacz	
~	~	Catherine Reed	
~	~	Nina Reeve	
~	~	Zara Roberts	
~	~	Timothy Spencer	

INVESTIGATORS AND COLLABORATORS: SITE STAFF

Staff on site delegation logs

City	Care_Site	Person_Name	Site_PI
~	~	David Stow	
~	~	Duncan Stow	
~	~	Harriet Taylor	
~	~	June Thomas	
~	~	Stacey Turner	
~	~	Cara Watson	
~	~	Terry Watson	
~	~	Bethany Wellington	
~	~	Nicola Williamson	
High Wycombe, UK	Wycombe Hospital	Katherine Hyde	PI
~	~	Ami Sabharwal	Ex-PI
~	~	Gerard Andrade	Co-I
~	~	Philip Camilleri	Co-I
~	~	Prabir Chakraborti	Co-I
~	~	Sean O'Cathail	Co-I
~	~	Thinn Pwint	Co-I
~	~	Maggie Aldersley	
~	~	Bhavna Badiani	
~	~	Jasvinder Bains	
~	~	Amarjit Bdesha	
~	~	Ans-Mari Bester	
~	~	Nicola Bowers	
~	~	Chrissie Butcher	
~	~	Janice Carpenter	
~	~	Penny Carter	
~	~	Evelyn Chan	
~	~	Tiffany Chan	
~	~	Christine Collins	
~	~	Anita Cserbane	
~	~	Benjamin Fairfax	
~	~	Claire Fernandez	
~	~	Siobhan Gettings	
~	~	Avinash Gupta	

INVESTIGATORS AND COLLABORATORS: SITE STAFF
Staff on site delegation logs

City	Care_Site	Person_Name	Site_PI
~	~	Neil Haldar	
~	~	Kathryn Herbert	
~	~	Emma Hogbin	
~	~	Manisha Joshi	
~	~	Roisin Kavanagh	
~	~	John Patrick Kelleher	
~	~	Rahul Kurup	
~	~	Erica Lieberman	
~	~	Rossana Mancinelli	
~	~	Sarah Manyangadze	
~	~	Moncy Mathew	
~	~	Susan McLain-Smith	
~	~	Vivek Mohan	
~	~	Aruna Nair	
~	~	Alice Ngumo	
~	~	Ileana Nguyen	
~	~	Catherine Northey	
~	~	Niki Panakis	
~	~	Andrew Protheroe	
~	~	Wasiru Saka	
~	~	Tracey Stammers	
~	~	Helena Stone	
~	~	Michelle Taylor-Siddons	
~	~	Samantha Thomas	
~	~	Sally Trent	
~	~	Neil Trew-Smith	
~	~	Gail Varley	
~	~	Janet Weir	
~	~	Hazel Wynn	
Huddersfield, UK	Huddersfield Royal Infirmary	Uschi Hofmann	PI
~	~	Nicolas Bryan	Co-I
~	~	Lucy Jones	Co-I
~	~	Deivasikamani Ramanujam	Co-I

INVESTIGATORS AND COLLABORATORS: SITE STAFF
Staff on site delegation logs

City	Care_Site	Person_Name	Site_PI
~	~	Samantha Turnbull	Co-I
~	~	Mohammad Irfan Alam	
~	~	Karen Bicknell	
~	~	Barbara Crosse	
~	~	Nicky Daker	
~	~	Stacey Freeth	
~	~	Lisa Gledhill	
~	~	Paula Gomes	
~	~	Lindsay Greenhalgh	
~	~	Denise Hancock	
~	~	Jane Hook	
~	~	Ibrar Hussain	
~	~	Hayley Inman	
~	~	Diane Kelly	
~	~	Mandy Madigan	
~	~	Lear Matapure	
~	~	Adam Mawer	
~	~	Belinda McLean	
~	~	Julie Millward	
~	~	Naledi Mzwimbi	
~	~	Monica Narasimham	
~	~	Rachel Parker	
~	~	Melanie Quesne	
~	~	Hannah Riley	
~	~	Kully Sandhu	
~	~	Lisa Shaw	
~	~	Kathryn Smith	
~	~	Katherine Tighe	
~	~	Christine Turner	
~	~	Rob Turner	
~	~	Miranda Usher	
~	~	Hayley Webster	
~	~	Tracy Wood	

INVESTIGATORS AND COLLABORATORS: SITE STAFF

Staff on site delegation logs

City	Care_Site	Person_Name	Site_PI
~	~	Emma Woodward	
~	~	Sharon Woolley	
Hull, UK	Princess Royal Hospital (Hull)	Robert Dealey	Ex-PI
~	~	Emma Bertram	
~	~	Suzy Bunton	
~	~	Christopher Hamilton	
~	~	Linda Hoggarth	
~	~	Claire Levesley	
~	~	Sarah Moffat	
Inverness, UK	Raigmore Hospital	Neil McPhail	PI
~	~	Anglise Addison	
~	~	Florence Anderson	
~	~	Seonaid Arnott	
~	~	Susan Bain	
~	~	Sudhir Borgaonkar	
~	~	Sandra Brown	
~	~	Karen Callum	
~	~	Audrey Campbell	
~	~	Denise Campbell	
~	~	Fiona Campbell	
~	~	Jane Campbell	
~	~	Margaret Chisholm	
~	~	Kay Kelly	
~	~	Charles Kodikara	
~	~	Michael Loynd	
~	~	Alison Macdonald	
~	~	Angela Macgregor	
~	~	Carol Macgregor	
~	~	Rachel Mackay	
~	~	Laura Maclennan	
~	~	Jude Madeleine	
~	~	Melanie McIlroy	
~	~	Mary McKenzie	

INVESTIGATORS AND COLLABORATORS: SITE STAFF
Staff on site delegation logs

City	Care_Site	Person_Name	Site_PI
~	~	Karina McQuiston	
~	~	Catriona Morrison	
~	~	Sean Neville	
~	~	Alison Nicholls	
~	~	Steve Nicholson	
~	~	Aristoula Papakostidi	
~	~	Marion Paterson	
~	~	Anne Marie Pollock	
~	~	Martin Russell	
~	~	Azmat Sadozye	
~	~	Ian Shread	
~	~	Georgina Simpson	
~	~	Glenda Sinclair	
~	~	Jane Sinclair	
~	~	Anna Skene	
~	~	Joan Stewart	
~	~	Una Taylor	
~	~	Zoe Urquhart	
~	~	David Whillis	
Ipswich, UK	Ipswich Hospital	Robert Brierly	PI
~	~	William Ine	Co-I
~	~	TJ Podd	Co-I
~	~	Deborah Abrams	
~	~	Debbie Austin	
~	~	Gautam Banerjee	
~	~	Sheen Cherian	
~	~	Jennifer Collins	
~	~	Peter Donaldson	
~	~	Charlotte Etheridge	
~	~	Ian Floodgate	
~	~	Mohsen Habib	
~	~	Adiba Hoodbhoy	
~	~	Kerry Howlett (nee Brown)	

INVESTIGATORS AND COLLABORATORS: SITE STAFF
Staff on site delegation logs

City	Care_Site	Person_Name	Site_PI
~	~	Sonia Kerridge	
~	~	Natalie Lloyd	
~	~	Matt Mendoza	
~	~	John Parry	
~	~	Paul Ridley	
~	~	Mandy Riley (nee Evans)	
~	~	Chris Rose	
~	~	Christopher Scrase	
~	~	Julie Simpson	
~	~	Julie Spurgeon	
~	~	Sarah Treece	
~	~	Yvonne Tricker	
~	~	Susan Upson	
~	~	Ramachandran Venkitaraman	
~	~	Joe Wells	
~	~	Angharad Williams	
~	~	Jo Woor	
Keighley, UK	Airedale General Hospital	Simon Brown	PI
~	~	Sohail Mughal	Co-I
~	~	Hayley Bates	
~	~	Louise Binns	
~	~	Carl Booth	
~	~	Lisa Bullough	
~	~	Nathalie Casanova	
~	~	Sue Cheeseman	
~	~	Katy Clarke	
~	~	Michael Crawford	
~	~	Gillian Darnbrook	
~	~	Fiona Farquhar	
~	~	Andrew Gash	
~	~	Jasmine Hartley	
~	~	Ann Henry	
~	~	Helen Henson	

INVESTIGATORS AND COLLABORATORS: SITE STAFF
Staff on site delegation logs

City	Care_Site	Person_Name	Site_PI
~	~	Pip Hill	
~	~	Ganesan Jeyasangar	
~	~	Ruth Johnson	
~	~	Rachel Kennedy	
~	~	Dan Lee	
~	~	Judy McAlister	
~	~	Sharron Parkinson	
~	~	Amy Pendrill	
~	~	Joseph Quinn	
~	~	Charlotte Richardson	
~	~	Satti Saggu	
~	~	Clara Sentamans	
~	~	Alison Shaw	
~	~	Liz Shenton	
~	~	Josie Snell	
~	~	Mandy Swanepoel	
~	~	Alison Swindells	
Kidderminster, UK	Kidderminster General Hospital	Lisa Capaldi	PI
~	~	Kirsty Clarke	Co-I
~	~	Paul Flinders	Co-I
~	~	Ayyaz Munawar	Co-I
~	~	Shaikh Rana	Co-I
~	~	Mark Churn	
~	~	Kate Field	
~	~	Monica Gauntlett	
~	~	Linda Higgins	
~	~	Hayley Hodson	
~	~	M Habib Khan	
~	~	Emma Marshall	
~	~	Hugh Morrow	
~	~	Sarah Moss	
~	~	Zeeshaan Parvez	
~	~	Patricia Rimell	

INVESTIGATORS AND COLLABORATORS: SITE STAFF
Staff on site delegation logs

City	Care_Site	Person_Name	Site_PI
~	~	Alison Rosoman	
~	~	Veronica Rowlands	
~	~	Sally Stringer (pr. Davis)	
~	~	Helen Tranter	
~	~	Jayne Tyler	
~	~	Ann White	
~	~	Julie Wollaston	
Kilmarnock, UK	Crosshouse Hospital	Margaret McKernan	
Lancaster, UK	Royal Lancaster Infirmary	Sophie Raby	
Larbert, UK	Forth Valley Royal Hospital	Norma Sidek	PI
~	~	Saurabh Borgaonkar	
~	~	Stephanie Brogan (nee Roddie)	
~	~	Maureen Hamill	
~	~	Eilidh Henderson	
~	~	Carolynn Lamb	
~	~	Amy Martin	
~	~	Stephen McKay	
~	~	Nadja Melquiot	
~	~	Adam Peters	
~	~	Lynn Prentice	
~	~	Joanne Robinson	
~	~	John Martin Russell	
~	~	Lesley Symon	
~	~	Seamus Teahan	
~	~	Anne Todd	
~	~	Patricia Turner	
~	~	Sally Young	
~	~	Alison Yule	
Leeds, UK	Cookridge Hospital	Richard Kaplan	
~	~	Anne Kiltie	
~	~	Carmel Loughrey	
Leeds, UK	Leeds General Infirmary	Caroline Bedford	
~	~	Adrian Joyce	

INVESTIGATORS AND COLLABORATORS: SITE STAFF
Staff on site delegation logs

City	Care_Site	Person_Name	Site_PI
Leeds, UK	St James University Hospital (Leeds)	William Cross	PI
~	~	Peter Whelan	Ex-PI
~	~	Naveen Vasudev	Co-I
~	~	Dolapo Ajayi	
~	~	Polapo Ajayi	
~	~	Javeria Akhtar	
~	~	Gemma Austin (nee Glover)	
~	~	Caroline Bedford	
~	~	Ian Boon	
~	~	David Bottomley	
~	~	Janet Brown	
~	~	James Cavanagh	
~	~	Judith Chapman	
~	~	John Chester	
~	~	Jude Clarke	
~	~	Anne Crossley	
~	~	Claire Daisey	
~	~	Luis Daverede	
~	~	Emily Davies	
~	~	Svetoslava Doshmanonska	
~	~	Judith Evans	
~	~	Kevin Franks	
~	~	Catherine Gray	
~	~	Maria Hall	
~	~	Ann Henry	
~	~	Jodene Hill	
~	~	Liz Hudson	
~	~	Satinder Jagdev	
~	~	Sunjay Jain	
~	~	Joseph Joji	
~	~	Adrian Joyce	
~	~	Mercy Kaiga	
~	~	Richard Kaplan	

INVESTIGATORS AND COLLABORATORS: SITE STAFF

Staff on site delegation logs

City	Care_Site	Person_Name	Site_PI
~	~	Richard Khafagy	
~	~	Anne Kiltie	
~	~	Sanjeev Kotwal	
~	~	Sam Lotfi	
~	~	Carmel Loughrey	
~	~	Emma Lundy	
~	~	Jade McCann	
~	~	Angela Morgan	
~	~	Hima Bindu Musunuru	
~	~	Catherine Parbutt	
~	~	Alan Paul	
~	~	Helen Payne	
~	~	Charlotte Pool	
~	~	Stephen Prescott	
~	~	Christy Ralph	
~	~	Hannah Roberts	
~	~	Sue Rodwell	
~	~	Krishna Shastry	
~	~	Sue Sibson	
~	~	Rafal Turo	
~	~	Hannah Wigginton	
~	~	Christopher Williams	
~	~	Lorraine Wiseman	
~	~	Ruiyang Yan	
Lincoln, UK	Lincoln County Hospital	Thiagarajan Sreenivasan	PI
~	~	Prantik Das	Co-I
~	~	Ana Fernandez-Ots	Co-I
~	~	Sindhu Ramarwothy	Co-I
~	~	Alfredo Addeo	
~	~	Simon Archer	
~	~	Suzanne Archer	
~	~	Christian Arias	
~	~	David Ballesteros-Quintail	

INVESTIGATORS AND COLLABORATORS: SITE STAFF
Staff on site delegation logs

City	Care_Site	Person_Name	Site_PI
~	~	Giuseppe Banna	
~	~	Karin Baria	
~	~	Sarah Bell	
~	~	Jayne Borley	
~	~	Susie Butler	
~	~	Diane Carey	
~	~	Helen Carolan	
~	~	Sarah Coombs	
~	~	Olesya Francis	
~	~	Annette Hilldrith	
~	~	Kathryn Hoare	
~	~	Kerri Johnson	
~	~	Andrew Judd	
~	~	Carol Lockwood	
~	~	Ray McDermott	
~	~	Yogesh Nishchal	
~	~	Maryanne Okubanjo	
~	~	Miguel Panades	
~	~	Kathryn Pearson	
~	~	Rhiannan Pegg	
~	~	Gunjan Phalod	
~	~	Jenny Salmon	
~	~	Andrew Sloan	
~	~	Rebecca Spencer	
~	~	Caroline Taylor	
~	~	Janet Tomlinson	
~	~	Elena Umbrarescu	
~	~	Laura Walsh	
~	~	Alyson Wilson	
Liverpool, UK	Royal Liverpool University Hospital	Zafar Malik	PI
~	~	Chinnamani Eswar	Co-I
~	~	Nicola Bermingham	
~	~	Lizzie Dale	

INVESTIGATORS AND COLLABORATORS: SITE STAFF
Staff on site delegation logs

City	Care_Site	Person_Name	Site_PI
~	~	Lynsey Dean	
~	~	Lisa Dobson (nee Child)	
~	~	Sharon Dunn (nee Johnson)	
~	~	Sue Green	
~	~	Julie Griffiths	
~	~	Paul Griffiths	
~	~	Jasima Latif	
~	~	Kevin McDonald	
~	~	Pauline Pilkington	
~	~	Dawn Porter	
~	~	Philip Reynolds	
~	~	Sandra Robinson	
~	~	Peter Robson	
~	~	Nidhi Sibal	
~	~	Katy Treherne	
~	~	Emma Whitby	
~	~	Pembe Yesildag	
Liverpool, UK	University Hospital Aintree	Peter Robson	PI
~	~	Ian Allen	
~	~	Wesley Artist	
~	~	Lucy Berresford	
~	~	Lisa Dobson (nee Child)	
~	~	Rachael Fergusson	
~	~	Julie Griffiths	
~	~	Paul Hill	
~	~	Lorraine Lancaster	
~	~	Haley McCulloch	
~	~	Leigh Pauls	
~	~	Sandra Robinson	
London, UK	Charing Cross Hospital	Alison Falconer	PI
~	~	Stephen Mangar	Co-I
~	~	Najma Ahmed	
~	~	Kwame Ansu	

INVESTIGATORS AND COLLABORATORS: SITE STAFF

Staff on site delegation logs

City	Care_Site	Person_Name	Site_PI
~	~	Gareth Barker	
~	~	Bindu Chikkamuniyappa	
~	~	Ross Dalton-Short	
~	~	Andrea Davis-Cook	
~	~	Steve Edwards	
~	~	Daisy Floyd	
~	~	Jill Gallagher	
~	~	Paul Kabuubi	
~	~	Zohanon Sabine Loko	
~	~	Ethna Mannion	
~	~	Akeema Paul	
~	~	Ibiyemi Sadare (Olaleye)	
~	~	Naveed Sarwar	
~	~	Stephanie Steadman	
~	~	Samantha Weller	
London, UK	Guy's Hospital (London)	Sarah Rudman	PI
~	~	Sarah Howiett	Co-I
~	~	Vishal Manik	Co-I
~	~	Chara Stavraka	Co-I
~	~	Awo Abdi	
~	~	Delali Adjogatse	
~	~	Ajay Aggarwal	
~	~	Fahim Ahmed	
~	~	Rayhan Ahmed	
~	~	Ramin Ajami	
~	~	Susanne Allan	
~	~	Stephanie Argue	
~	~	Caterina Aversa	
~	~	Eva Batovska	
~	~	Ronald Beaney	
~	~	Thomas Bird	
~	~	Trevor Bott	
~	~	Sabeeh Butt	

INVESTIGATORS AND COLLABORATORS: SITE STAFF

Staff on site delegation logs

City	Care_Site	Person_Name	Site_PI
~	~	Declan Cahill	
~	~	Jozer Calara	
~	~	Donna Cassidy	
~	~	Emilia Caverly	
~	~	Charleen Chan Wah Hak	
~	~	Belinda Chitando	
~	~	Simon Chowdhury	
~	~	Chi Yee Chung	
~	~	Sharon Clovis	
~	~	Danielle Crawley	
~	~	Francesca Curran	
~	~	Kafui Dossa	
~	~	Michelle Dutton	
~	~	Deborah Enting	
~	~	Louisa Fleure	
~	~	Angel Garcia-Imhof	
~	~	Tahereh Ghadimi	
~	~	Sharmistha Ghosh	
~	~	Clare Gilson	
~	~	Claire Glendon	
~	~	Charalampos Gousis	
~	~	Teresa Guerrero-Urbano	
~	~	Sarah Hargreaves	
~	~	Peter Harper	
~	~	Simon Hughes	
~	~	Sheeba Irshad	
~	~	Ruth Johnson	
~	~	Eleni Josephides	
~	~	Debra Josephs	
~	~	Lucy Juggins	
~	~	Srivani Kandasamy	
~	~	Matthaius Kapiris	
~	~	Anna Karpathakis	

INVESTIGATORS AND COLLABORATORS: SITE STAFF
Staff on site delegation logs

City	Care_Site	Person_Name	Site_PI
~	~	Muhammad Khan	
~	~	Rosalind Kieran	
~	~	Sarah King	
~	~	Ursula Kirwan	
~	~	Lawrence Krieger	
~	~	Hartmut Kristeleit	
~	~	Cheryl Lawrence	
~	~	Archie Macnair	
~	~	Thubeena Manickavasagar	
~	~	Louisa McDonald	
~	~	Sharon McPherson	
~	~	Vasiliki Michalarea	
~	~	Stephen Morris	
~	~	Vinod Mullassery	
~	~	Ngozi Muoneke	
~	~	Janette Nichol	
~	~	Emma O'Connor	
~	~	Temi Olusi	
~	~	Anna Parker	
~	~	Elias Pintus	
~	~	Rick Popert	
~	~	Vivien Quan	
~	~	Antonio Querol-Rubiera	
~	~	Lucy Reed	
~	~	Philip Reynolds	
~	~	Catherine Rogers	
~	~	Hannah Rush	
~	~	Linda Shephard	
~	~	Sumeet Sisodia	
~	~	Susie Slater	
~	~	Helen Snow	
~	~	Anita Soma	
~	~	Thomas Spencer	

INVESTIGATORS AND COLLABORATORS: SITE STAFF

Staff on site delegation logs

City	Care_Site	Person_Name	Site_PI
~	~	Rushan Sylva	
~	~	Kiruthikah Thillai	
~	~	Rebecca Todd	
~	~	Daniel Tong	
~	~	Gerry Trillana	
~	~	Eirini Tsotra	
~	~	Nikolaos Tsoukalas	
~	~	Jennifer Turner	
~	~	Suzanne Vizor	
~	~	Mark Voskoboynik	
~	~	Sally Walker	
~	~	Rebecca Way	
~	~	Kate Williams	
~	~	Yin Wu	
~	~	Kamarul Zaki	
London, UK	Hammersmith Hospital	Alison Falconer	PI
~	~	Stephen Mangar	Co-I
~	~	Ilyas Ali	
~	~	Steve Edwards	
~	~	Nikki Kettley	
~	~	Emily Pickford	
~	~	Regina Storch	
London, UK	King George Hospital	Neil Fisher	
~	~	Ramachandran Subramaniam	
London, UK	Queen Elizabeth Hospital (Woolwich)	Sindu Vivekanandan	PI
~	~	Vinod Mullessey	Ex-PI
~	~	Vinod Mullassery	Co-I
~	~	Elias Pintus	Co-I
~	~	Rayhan Ahmed	
~	~	Shahreen Ahmed	
~	~	Jagdev Bains	
~	~	Laura Beschizza	
~	~	Belinda Chitando	

INVESTIGATORS AND COLLABORATORS: SITE STAFF
Staff on site delegation logs

City	Care_Site	Person_Name	Site_PI
~	~	Sharai Chitando	
~	~	Suzanne Chukundah	
~	~	Miriam Cottle	
~	~	Nadia El-Sayed	
~	~	Martha Handousa	
~	~	Rachel Harper	
~	~	Hazel Harrop	
~	~	Nigel Holmes	
~	~	Simon Hughes	
~	~	Abhijit Jadhav	
~	~	Abel Jalloh	
~	~	Bridget Kabagambe	
~	~	Arunansu Kar	
~	~	Sagira Khatun	
~	~	Hartmut Kristeleit	
~	~	Maria Liskova	
~	~	Luke Maidment	
~	~	Nick Maisey	
~	~	Joyce Maravi	
~	~	Jennifer Martin	
~	~	Theodorah Nago	
~	~	Melody Ncube	
~	~	Eti Omoregie	
~	~	Samia Pilgrim	
~	~	Lee Porin	
~	~	Philip Reynolds	
~	~	Thomas Sarkodie	
~	~	Aarti Shah	
~	~	Anne-Marie Vindidu	
~	~	Shanna Wilson	
London, UK	Royal Free Hospital	Sarah Needleman	PI
~	~	Maria Vilarino-Varela	Ex-PI
~	~	Magdalena Kubiak	Co-I

INVESTIGATORS AND COLLABORATORS: SITE STAFF
Staff on site delegation logs

City	Care_Site	Person_Name	Site_PI
~	~	Nicola Rosenfelder	Co-I
~	~	Emily Scott	Co-I
~	~	Kate Smith	Co-I
~	~	Grant Stewart	Co-I
~	~	Naomi Anderson	
~	~	Juniebel Cooke	
~	~	Emma Douch	
~	~	Sara Fawcitt	
~	~	Jessica Hunt	
~	~	Claire Jarvis	
~	~	Marisa Lanzman	
~	~	Ruochen Li	
~	~	Su Fung Lo	
~	~	Kharishma Makani	
~	~	Angela McCadden	
~	~	Sabina Melander	
~	~	Aarti Nandani	
~	~	Lorna O'Shea	
~	~	Anna Osadcow	
~	~	Katherine Pigott	
~	~	Hannah Powell	
~	~	Kaliyanee Ramtohul	
~	~	Daniel Smith	
~	~	Tesha Suddason	
~	~	Elizabeth Woodford	
London, UK	Royal Marsden Hospital (London)	Vincent Khoo	PI
~	~	Ewan Chapman	Co-I
~	~	Laillah-Crystal Banda	
~	~	Trevor Bott	
~	~	Karen Brooks	
~	~	Karen Chan	
~	~	Rosalind Eeles	
~	~	Nicola Harman	

INVESTIGATORS AND COLLABORATORS: SITE STAFF
Staff on site delegation logs

City	Care_Site	Person_Name	Site_PI
~	~	Holly Hogan	
~	~	James Lowe	
~	~	Nicola Lucas	
~	~	Chloe McCormack	
~	~	Jennifer Morrison	
~	~	Vedang Murthy	
~	~	Annette Musallam	
~	~	Marisa Pinto Peixoto	
~	~	Suraya Quadir	
~	~	Alison Reid	
~	~	Debbie Rolfe	
~	~	Bernard Sill	
~	~	Bernard Siu	
~	~	Ruth Stafferton	
~	~	Helen Stidwell	
~	~	Sarah Storrs	
~	~	Debra Townsend-Thorn	
~	~	Nicholas Van As	
~	~	Vijitha Vijayakumar	
~	~	Li Wancheung	
London, UK	St Bartholomews Hospital (London)	Karen Tipples	PI
~	~	Paula Wells	Co-I
~	~	Marina Baccarini	
~	~	P Cathcart	
~	~	Samantha Chetiyawardana	
~	~	Fatjon Dekaj	
~	~	Shahanara Ferdous	
~	~	Stephanie Gibbs	
~	~	Denise Humfress	
~	~	Resmi Jayachandran	
~	~	Janet Kiff	
~	~	Cheryl Lawrence	
~	~	Wing-Kin Liu	

INVESTIGATORS AND COLLABORATORS: SITE STAFF

Staff on site delegation logs

City	Care_Site	Person_Name	Site_PI
~	~	Sebastien Martin	
~	~	Alastair Nicholson	
~	~	Jude Nixon	
~	~	Janet Oladimeji	
~	~	Hannah Payne	
~	~	Oscar Riches	
~	~	Jonathon Shamash	
~	~	Cavitha Vivekananthan	
London, UK	St Georges Hospital (London)	Mehran Afshar	PI
~	~	Laura Camburn	Co-I
~	~	Jason Chow	Co-I
~	~	Nia Alsamarrai	
~	~	Michael Brown	
~	~	Sue Cromarty	
~	~	Alice Dainty	
~	~	Deirdre Daly	
~	~	Serena Dover	
~	~	Gelareh Eslamian	
~	~	Claire Gilmartin	
~	~	Sophie Golden	
~	~	Jane Gregg	
~	~	Hakim Guessous	
~	~	Anne Haldeos	
~	~	Sam Hollingworth	
~	~	Geoffrey Howell	
~	~	Mohammed Mahgoub	
~	~	Roxane Mather	
~	~	Sophie McGrath	
~	~	Asha Mistry	
~	~	Uforma Ogrigri	
~	~	Chandni Patel	
~	~	Lisa Pickering	
~	~	Mark Quarrell	

INVESTIGATORS AND COLLABORATORS: SITE STAFF

Staff on site delegation logs

City	Care_Site	Person_Name	Site_PI
~	~	Debbie Rolfe	
~	~	Helen Tighe	
~	~	Juel Tuazon	
~	~	Robert Varro	
London, UK	St Marys Hospital (London)	Alison Falconer	PI
~	~	Melloney Allnutt	
~	~	Gareth Barker	
~	~	Angela Chamberlain	
~	~	Bindu Chikkamuniyappa	
~	~	Laura Custins	
~	~	Andrea Davis-Cook	
~	~	Steve Edwards	
~	~	Daisy Floyd	
~	~	Gillian Hornzee	
~	~	Joy Liao	
~	~	Joy Liau	
~	~	Zohanon Sabine Loko	
~	~	Stephen Mangar	
~	~	Akeema Paul	
~	~	Severine Rey	
~	~	Simon Stewart	
London, UK	University College Hospital	Ursula McGovern	PI
~	~	Richard Kaplan	Co-I
~	~	Mark Linch	Co-I
~	~	Heather Payne	Co-I
~	~	Adrienne Abioye	
~	~	Didem Agdiran	
~	~	Javeria Akhtar	
~	~	Hannah Ansell	
~	~	Uzma Asghar	
~	~	Natasha Aslam	
~	~	Aileen Austria	
~	~	Holly Baker (nee. Wing)	

INVESTIGATORS AND COLLABORATORS: SITE STAFF
Staff on site delegation logs

City	Care_Site	Person_Name	Site_PI
~	~	Ignacio Blanch	
~	~	Judith Cave	
~	~	Noan-Minh Chau	
~	~	Patricia Danaswamy	
~	~	Reena Davda	
~	~	Danny Garrett	
~	~	Annelies Gillesen	
~	~	Roshni Goel	
~	~	Stephen Harland	
~	~	Yemi Ilumoka	
~	~	Bihani Kularatne	
~	~	Jane Leach	
~	~	Suzy Lowi	
~	~	John Masters	
~	~	Anita Mitra	
~	~	Dieo Ottaviani	
~	~	Kristian Warnes	
~	~	Agnieska Zielonka	
~	~	Helene Zilkha	
London, UK	University College London	Holly Baker (nee. Wing)	
London, UK	University Hospital Lewisham	Aarti Shah	
London, UK	Whittington Hospital (London)	Simon Wan	
Maidstone, UK	Maidstone Hospital	Patryk Brulinski	PI
~	~	Delali Adjogatse	
~	~	Claire Baldry	
~	~	Sharon Beesley	
~	~	Jess Brady	
~	~	Vivienne Breen	
~	~	Jane Brown	
~	~	Su Burrage	
~	~	Clare Calvert	
~	~	Amanda Clarke	
~	~	Laura Clayton	

INVESTIGATORS AND COLLABORATORS: SITE STAFF
Staff on site delegation logs

City	Care_Site	Person_Name	Site_PI
~	~	Emma Craske	
~	~	Alison Davison	
~	~	Anna English	
~	~	Clary Evans	
~	~	Matthew Fittall	
~	~	Gavin Fossey	
~	~	Louise Hooper-Gilham	
~	~	Carmel Jope	
~	~	Emma Kipps	
~	~	Kathryn Lees	
~	~	Sarah Martins	
~	~	Romaana Mir	
~	~	Jane Murray	
~	~	Ian Pamphlett	
~	~	Joanne Patterson	
~	~	Ann Phillips	
~	~	Alison Richards	
~	~	Verity Roberts	
~	~	Alicia Synowiec	
~	~	Henry Taylor	
~	~	Katy Taylor	
~	~	Amie Thomas	
~	~	Lisa Tribe	
~	~	Joanne Williams	
~	~	Claudia Woodger	
Manchester, UK	Christie Hospital	Noel Clarke	PI
~	~	Ruth Conroy	Co-I
~	~	Christoph Oing	Co-I
~	~	Ali Al-Hashimi	
~	~	Susan Arrand	
~	~	Sreeja Aruketty	
~	~	Ian Bottomley	
~	~	Anna Bowron	

INVESTIGATORS AND COLLABORATORS: SITE STAFF

Staff on site delegation logs

City	Care_Site	Person_Name	Site_PI
~	~	Michael Braun	
~	~	Anna Bruzzan	
~	~	Megan Bunce	
~	~	Emma Burke	
~	~	Sharon Capper	
~	~	Clara Chan	
~	~	Stephen Chin	
~	~	Ananya Choudhury	
~	~	Richard Cowan	
~	~	Catherine Coyle	
~	~	Sue Davison	
~	~	Sarah-Ellen Ellen (née McCarthy)	
~	~	Tony Elliott	
~	~	Thiraviyam Elumalai	
~	~	Kim Fair	
~	~	Stefanie Fisder	
~	~	Laura Flanagan	
~	~	Silke Gillessen	
~	~	Lynne Gilmore	
~	~	Sarah Green	
~	~	Amber Hart	
~	~	Charlotte Heywood	
~	~	Andrew Hudson	
~	~	Cathryn James	
~	~	A Jegannathen	
~	~	Cathryn Jones	
~	~	Ather Kazmi	
~	~	Jacqueline Livsey	
~	~	John Logue	
~	~	Emma Lowther	
~	~	Jeanette Lyons	
~	~	Damian McCall	
~	~	Damian McCaul	

INVESTIGATORS AND COLLABORATORS: SITE STAFF

Staff on site delegation logs

City	Care_Site	Person_Name	Site_PI
~	~	Samah Mughal	
~	~	Roonak Nazari	
~	~	Kate O'Connor	
~	~	Jackie O'Dwyer	
~	~	Joanne Oliver	
~	~	Ekugbe Onogbe	
~	~	Ekugbe Onoge	
~	~	Alkesh Patel	
~	~	Kamlesh Patel	
~	~	Maria Petsa	
~	~	Catherine Pettersen	
~	~	Vijay Ramani	
~	~	Catherine Redshaw	
~	~	Vijay Sangar	
~	~	Sue Seifi	
~	~	Sarah-Ellen Smith	
~	~	Yee Pei Song	
~	~	Willemijn Spoor	
~	~	Martin Swinton	
~	~	Viv Thomas	
~	~	David Thompson	
~	~	Shaun Tolan	
~	~	Anna Tran	
~	~	Trishna Uttamlal	
~	~	Marie Woolley	
~	~	Lucy Worsley	
~	~	James Wylie	
~	~	You Yone	
~	~	salina tsui	
Manchester, UK	Withington Hospital	Vijay Sangar	PI
~	~	Vijay Ramani	Co-I
~	~	Humera Ahmed	
~	~	Linda Bailey	

INVESTIGATORS AND COLLABORATORS: SITE STAFF

Staff on site delegation logs

City	Care_Site	Person_Name	Site_PI
~	~	Vivienne Benson	
~	~	Julie Bramley	
~	~	Rebecca Corless	
~	~	Tania Cutts	
~	~	Annie Duffy	
~	~	Beatriz Duran Jimenez	
~	~	A. Emara	
~	~	Kathryn Fellows	
~	~	Anna Gipson	
~	~	Stephanie Hargreaves	
~	~	Helen Haydock	
~	~	Tarnya Hulme	
~	~	Damian McCall	
~	~	Thobekile Mthethwa	
~	~	Fiona Murtagh	
~	~	Lillian Partington	
~	~	Lindsay Piper	
~	~	Tracey Platt	
~	~	Catherine Redshaw	
~	~	Karen Robb	
~	~	Janet Smith	
~	~	Lorraine Turner	
~	~	James Wylie	
Manchester, UK	Wythenshawe Hospital	Vijay Sangar	PI
~	~	Linda Bailey	
~	~	Vivienne Benson	
~	~	Angela Chrisopoulou	
~	~	Annie Duffy	
~	~	Beatriz Duran Jimenez	
~	~	A. Emara	
~	~	Julie Fielding	
~	~	Angela Gowrie	
~	~	Wendy Guest	

INVESTIGATORS AND COLLABORATORS: SITE STAFF

Staff on site delegation logs

City	Care_Site	Person_Name	Site_PI
~	~	Sarah Liptrott	
~	~	Claire McGuire	
~	~	Kirsty Melia	
~	~	Thobekile Mthethwa	
~	~	Lindsay Piper	
~	~	Tracey Platt	
~	~	Kathryn Slevin	
Margate, UK	Queen Elizabeth The Queen Mother Hospital	Carys Thomas	PI
~	~	Albert Edwards	Co-I
~	~	Jessica Little	Co-I
~	~	Natasha Mithal	Co-I
~	~	Rakesh Raman	Co-I
~	~	Jennifer Turner	Co-I
~	~	Ifigenia Vasiliadou	Co-I
~	~	Louise Allen	
~	~	Bonny Appleby	
~	~	Sharon Beesley	
~	~	Hayley Blackgrove	
~	~	Tracy Boakes	
~	~	Patryk Brulinski	
~	~	Julie Buckley	
~	~	Miguel Capo-Mir	
~	~	Natalie Catt	
~	~	Mathilda Cominos	
~	~	Denise Crawford	
~	~	Nikki Crisp	
~	~	Steve Dann	
~	~	Julie-Ann Davies	
~	~	Susan Drakeley	
~	~	Clary Evans	
~	~	Sam Gibson	
~	~	Andrew Gillian	
~	~	Louise Gladwell	

INVESTIGATORS AND COLLABORATORS: SITE STAFF

Staff on site delegation logs

City	Care_Site	Person_Name	Site_PI
~	~	Coral Greenstreet	
~	~	Sandra Holness	
~	~	Laura Kehoe	
~	~	Sue Kelly	
~	~	Rachel Larkins	
~	~	Kathryn Lees	
~	~	Sarah Lightfoot	
~	~	Sarah Lines	
~	~	Margaret Lipsham	
~	~	Sydnie Loveland	
~	~	Rohit Malde	
~	~	Kim Mears	
~	~	Sharon Middleton	
~	~	Arafat Mirza	
~	~	Kannon Nathan	
~	~	Udaiveer Panwar	
~	~	Claire Pelham	
~	~	Karen Robinson	
~	~	Susan Rogers	
~	~	Lesley Rose	
~	~	Cindy Slater	
~	~	Mathini Sridharan	
~	~	Stephane Tankoua	
~	~	Katy Taylor	
~	~	Kim Travis	
~	~	Alba Tubau	
~	~	Kathleen (Kathy) Walsh	
~	~	Paula Whichelo	
~	~	Claire White	
~	~	Jo Williams	
~	~	Joanne Williams	
~	~	Elizabeth Williamson	
~	~	Victoria Williamson	

INVESTIGATORS AND COLLABORATORS: SITE STAFF

Staff on site delegation logs

City	Care_Site	Person_Name	Site_PI
~	~	Marian Wood	
~	~	Linda Wray	
~	~	Hilary Zurakovsky	
Middlesbrough, UK	James Cook University Hospital	Clive Peedell	PI
~	~	Alison Barnes	
~	~	Helen Carver	
~	~	David Chadwick	
~	~	Alison Chilvers	
~	~	Helen Dunn	
~	~	Claire Elliott	
~	~	Vicky Hanlon	
~	~	John Hardman	
~	~	Anne Hardwick	
~	~	Keith Harland	
~	~	Charlotte Jacobs(née Kitching)	
~	~	Paul Jones-King	
~	~	Mohammed Kagzi	
~	~	Sarah Kiddell	
~	~	Carol Long	
~	~	Emanuela Mahmoud	
~	~	Sarah McAuliffe	
~	~	Julia McBride	
~	~	Lynne Naylor	
~	~	Lisa Peacock (nee Wayman)	
~	~	Julie Potts	
~	~	Steven Pratt	
~	~	Fiona Rowling	
~	~	Luca Settimo	
~	~	Devadasan Shakespeare	
~	~	Agnieszka Skotnicka	
~	~	Emma Thompson	
~	~	Jane Thompson	
~	~	Katherine Tyler	

INVESTIGATORS AND COLLABORATORS: SITE STAFF
Staff on site delegation logs

City	Care_Site	Person_Name	Site_PI
~	~	Hans Van der Voet	
~	~	Andrea Watson	
~	~	David Wilson	
~	~	Jason Wong	
~	~	Maha Zarroug	
Newcastle upon Tyne, UK	Newcastle General Hospital	Judith Moore	
Newcastle-upon-Tyne, UK	Freeman Hospital	Ashraf Azzabi	PI
~	~	John Frew	Co-I
~	~	Shahid Iqbal	Co-I
~	~	Rhona McMenemin	Co-I
~	~	Ian Pedley	Co-I
~	~	Craig Alderson	
~	~	Katie Bain	
~	~	Lucy Blackwell	
~	~	Lauren Boal	
~	~	Penny Bradley	
~	~	Elle Cameron	
~	~	Ian Campbell	
~	~	Roger Carr	
~	~	Kay Carson	
~	~	Robert Chandler	
~	~	Caroline Dobeson	
~	~	Hannah Downs	
~	~	Sue Farrell	
~	~	Hazel Forsyth	
~	~	Elaine Greaves	
~	~	Noor Harris	
~	~	Amanda Henderson	
~	~	Andrew Herridge	
~	~	Ben Hood	
~	~	Ann Hudson	
~	~	Laura Jameson	
~	~	Thomas Jarvis	

INVESTIGATORS AND COLLABORATORS: SITE STAFF
Staff on site delegation logs

City	Care_Site	Person_Name	Site_PI
~	~	Xue Jiang	
~	~	Irene Jobson	
~	~	Mark Johnson	
~	~	Sunita Kholi	
~	~	Emma King	
~	~	Sunita Kollu	
~	~	Lavanya Mariappan	
~	~	Hazel Masson	
~	~	Peter Murphy	
~	~	Lesley Naik	
~	~	Gemma O'Neill	
~	~	Sarah Osborne	
~	~	Edgar Paez	
~	~	Elizabeth Reay	
~	~	Georgia Ross	
~	~	Sarah Rowling	
~	~	Jenny Smith	
~	~	Marianne Smith	
~	~	Naeem Soomro	
~	~	Carole Stobbart	
~	~	Julie Thohig	
~	~	Dianne Turner	
~	~	Dianne Wake	
~	~	Nichola Waugh	
Newport, UK	St Mary's Hospital (Newport)	Alison Brown	
~	~	Elizabeth Harrison	
~	~	Kudingila Madhava	
~	~	Tracey Tidbury	
~	~	Cindy Whitbread	
North Shields, UK	North Tyneside General Hospital	Mark Johnson	
Northampton, UK	Northampton General Hospital	Rachel Gabitass	
Northwood, UK	Mount Vernon Hospital	Peter Hoskin	PI
~	~	Viwod Mullassery	Ex-PI

INVESTIGATORS AND COLLABORATORS: SITE STAFF
Staff on site delegation logs

City	Care_Site	Person_Name	Site_PI
~	~	Hamoun Rozati	Co-I
~	~	Sara Abbassi	
~	~	Mohammed Abdul-Latif	
~	~	Farhan Ahmed	
~	~	Roberto Alonzi	
~	~	Nicola Anyamene	
~	~	Freya Ball	
~	~	Dolan Basak	
~	~	Rose Bell	
~	~	Neel Bhuva	
~	~	Sam Bosompem	
~	~	Jennifer Chard	
~	~	Lai Cheng Yew	
~	~	Helen Cladd	
~	~	Lucy Collins	
~	~	Janaka Cooray	
~	~	Nicola Cutmore	
~	~	Nazma Damani	
~	~	Paolo De Jesu	
~	~	Jeanette Dickson	
~	~	Kari Evans	
~	~	Jessica Finch	
~	~	Shiv Gayadeen	
~	~	Shaista Harpeer	
~	~	Olivia Hatcher	
~	~	Robert Hughes	
~	~	Rakhi Jain	
~	~	Suzanne Jenkins	
~	~	Bhanthi Kanagaratnam	
~	~	Sapna Kaur	
~	~	Rachael Khong	
~	~	Joanne Kosmin	
~	~	Paulina Kowalewska	

INVESTIGATORS AND COLLABORATORS: SITE STAFF

Staff on site delegation logs

City	Care_Site	Person_Name	Site_PI
~	~	Shakeda Lakha	
~	~	Sonia Li	
~	~	Elaine Lousley	
~	~	Henry Mandeville	
~	~	Jessica Milner	
~	~	Russell Moule	
~	~	Peter Ostler	
~	~	Kasia Owczarczyk	
~	~	Hannah Phillips	
~	~	Alice Ramsden	
~	~	Aamna Rashid	
~	~	Tahmina Shakil	
~	~	Mausam Singhera	
~	~	Linda Swaney	
~	~	David Tan	
~	~	Hannah Tharmalingam	
~	~	Harsha Vara	
~	~	Charlotte Westbury	
~	~	M Williams	
~	~	Katie Wood	
~	~	David Woolf	
~	~	Huiqi Yang	
~	~	Lai-Cheng Yew	
~	~	Kent Yip	
~	~	Claire Zane	
Nottingham, UK	Nottingham University Hospitals, City Campus	Santhanam Sundar	PI
~	~	Sadia Abdullah	Co-I
~	~	Eliot Chadwick	Co-I
~	~	Junhao Lim	Co-I
~	~	Rohan Tharaka	Co-I
~	~	Georgina Walker	Co-I
~	~	Leanne Alder	
~	~	Alex Blades	

INVESTIGATORS AND COLLABORATORS: SITE STAFF
Staff on site delegation logs

City	Care_Site	Person_Name	Site_PI
~	~	Matthew Brazkiewicz	
~	~	Louise Brookes	
~	~	Katie Carter	
~	~	Rena Chauhan	
~	~	Rachael Chivers	
~	~	Chin Chong	
~	~	Owen Cole	
~	~	Jade Eggleton	
~	~	Susan Elliott	
~	~	Charlotte Ellis	
~	~	Carol Gooch	
~	~	Stacey Green	
~	~	Lucy Howard	
~	~	Camille Hutchinson	
~	~	Daniel Kumar	
~	~	Adele Malson	
~	~	Jamie Mills	
~	~	Kayleigh Mills	
~	~	Kathryn Moore	
~	~	Asmaa Sa Omer	
~	~	Maeve Pomeroy	
~	~	Tin Sang-Tsang	
~	~	Daniel Saunders	
~	~	Ian Sayers	
~	~	Ewan Shawcroft	
~	~	Tania Slater	
~	~	Anita Stevenson	
~	~	Phillipa Sum	
~	~	Jacob Szolin-Jones	
~	~	Sarah Taylor	
~	~	Rohan Tharakan	
~	~	Hannah Thurlow	
~	~	Caitlin Todd	

INVESTIGATORS AND COLLABORATORS: SITE STAFF

Staff on site delegation logs

City	Care_Site	Person_Name	Site_PI
~	~	Sarah Widdowson	
Nuneaton, UK	George Eliot Hospital	Yakhub Khan	PI
~	~	Inderjit Atwal	
~	~	Jacob Bourne	
~	~	Andrew Chan	
~	~	Rachel Fergusson	
~	~	Sarah Fergusson	
~	~	Kerry Flahive	
~	~	Jessica Gunn	
~	~	Michaela Hill	
~	~	Pritpal Klear	
~	~	Jeanette Knapp	
~	~	Judith Lake	
~	~	Holly Lawrence	
~	~	Alison McCallum	
~	~	Andrea Mills	
~	~	Albert Mislang	
~	~	Sabiya Nasima	
~	~	Rachael Oates	
~	~	Winni Singh	
~	~	Melanie Taylor	
~	~	Andrew White	
~	~	Jenna Williams	
Oldham, UK	Royal Oldham Hospital	Ruth Conroy	PI
~	~	Ananya Choudhury	Co-I
~	~	Parth Desai	Co-I
~	~	Ehab Ibrahim	Co-I
~	~	Shaveta Mehta	Co-I
~	~	Anna Tran	Co-I
~	~	Mohammad Abutarb	
~	~	Joanne Allsop	
~	~	Hadia Ashraf	
~	~	Suzanne Bland	

INVESTIGATORS AND COLLABORATORS: SITE STAFF

Staff on site delegation logs

City	Care_Site	Person_Name	Site_PI
~	~	Wendy Cook	
~	~	Anthea Cree	
~	~	Kanal Gupta	
~	~	Ruth Halford	
~	~	Terence Hinton	
~	~	Shabaz Hussain	
~	~	Joanne Johnson	
~	~	Dawn Johnstone	
~	~	Richard Jones	
~	~	Helen Joyce	
~	~	Stephen Kennedy	
~	~	Victoria Lavin	
~	~	Mark Livingstone	
~	~	Jacqueline Livsey	
~	~	Peter Mbanu	
~	~	Jemma McLaughlin	
~	~	Leena Mistry	
~	~	Udeme Ohia	
~	~	Anna Pracz	
~	~	Kamala Ramatar	
~	~	Joanne Reed	
~	~	Agata Rembielak	
~	~	Dellesa Robinson	
~	~	Lyndsay Scarratt	
~	~	Shazril Imran Shaukat	
~	~	Amy Slack	
~	~	Kirstie Smith	
~	~	Hwoeifen Soohoo	
~	~	Richard Walshaw	
Oxford, UK	Churchill Hospital	Andrew Protheroe	PI
~	~	Daniel Ajzensztejn	Co-I
~	~	Gerard Andrade	Co-I
~	~	Philip Camilleri	Co-I

INVESTIGATORS AND COLLABORATORS: SITE STAFF
Staff on site delegation logs

City	Care_Site	Person_Name	Site_PI
~	~	Meenali Chitnis	Co-I
~	~	David J Cole	Co-I
~	~	Benjamin Fairfax	Co-I
~	~	Avinash Gupta	Co-I
~	~	Katherine Hyde	Co-I
~	~	Ami Sabharwal	Co-I
~	~	Robert Stuart	Co-I
~	~	Gemma Austin (nee Glover)	
~	~	Magdalena Benysek	
~	~	Lauren Booker	
~	~	Jane Boutflower	
~	~	Rosita Broderick	
~	~	Leigh Burns	
~	~	Anju Chalin	
~	~	Henry Chesson	
~	~	Richard Cousins	
~	~	Charlotte Davies	
~	~	Hugo De La Pena	
~	~	Ana De Veciana	
~	~	Jane Gibbard	
~	~	Will Goodman	
~	~	Trish Green	
~	~	Tessa Greenhalgh	
~	~	Elizabeth Hadley	
~	~	Silke Hahnewald	
~	~	Rachel Hart	
~	~	Katherine Jacob	
~	~	Patrycja Jastrzebska	
~	~	Evanthia Komninidou	
~	~	Sarah Lawrey	
~	~	Sarah Markus	
~	~	Kerrie Marston	
~	~	Paul Colin Miller	

INVESTIGATORS AND COLLABORATORS: SITE STAFF

Staff on site delegation logs

City	Care_Site	Person_Name	Site_PI
~	~	Matthew Mooney	
~	~	Sandra Mukkath	
~	~	Ann Murphy	
~	~	Julie Pinder	
~	~	Mark Prentice	
~	~	Thinn Pwint	
~	~	Laura Robledo	
~	~	Naveen Sankighatta	
~	~	Elaine Sugden	
~	~	Swapna Thummala	
~	~	Mark Tuthill	
~	~	Usharani Devi Wahengbam	
~	~	James Wakelin	
~	~	Robert Watson	
~	~	Sandie Wellman	
~	~	Kelly Wigglesworth	
~	~	Jo Wilson	
~	~	Martha Woodward	
~	~	Simon Wyatt	
~	~	Hazel Wynn	
Paisley, UK	Royal Alexandra Hospital	Tiago Rodrigues	
Poole, UK	Poole Hospital	Sue Brock	PI
~	~	Perric Crellin	Co-I
~	~	Joseph Davies	Co-I
~	~	Yogesh Nishchal	Co-I
~	~	Neal Beamish	
~	~	Hilary Blaney	
~	~	Deryck Burton	
~	~	Felicity Clapp	
~	~	Elizabeth Clarke	
~	~	Teresa Coffin	
~	~	Joe Davies	
~	~	Nichola Downs	

INVESTIGATORS AND COLLABORATORS: SITE STAFF

Staff on site delegation logs

City	Care_Site	Person_Name	Site_PI
~	~	Savina Elitova	
~	~	Maxine Flubacher	
~	~	Sally Gillespie	
~	~	Louise Heckford	
~	~	Amanda Iskender	
~	~	Lyn Jackson	
~	~	Stephanie Jones	
~	~	May Lwin	
~	~	Fiona Mellor	
~	~	Sally Munden	
~	~	Kate Mutendera	
~	~	Sara Orford	
~	~	Sarah Patch	
~	~	Sharon Power	
~	~	Sandy Pressdee	
~	~	Sophie Rix	
~	~	Susan Saxby	
~	~	Lee Tbaily	
~	~	Becky Troke	
~	~	Kate Urquhart	
~	~	Craig Vincent	
~	~	Emma Wesley	
~	~	Roger Wheelwright	
~	~	Delia Whiteman	
~	~	Emma Williams	
~	~	Elizabeth Woodward	
~	~	Seonaid Wright	
Portadown, UK	Craigavon Area Hospital	Judith Carser	PI
~	~	Fionnuala Houghton	Co-I
~	~	Leanne McCourt	
Portsmouth, UK	Queen Alexandra Hospital	Joanna Gale	PI
~	~	Shyamkia Acharige	Co-I
~	~	Oluwatobi Adeagbo	Co-I

INVESTIGATORS AND COLLABORATORS: SITE STAFF

Staff on site delegation logs

City	Care_Site	Person_Name	Site_PI
~	~	Giuseppe Banna	Co-I
~	~	Joanna Hack	Co-I
~	~	Harliana Mohd Yusof	Co-I
~	~	Syed Shah	Co-I
~	~	Jillian Andrews	
~	~	Kathy Blight	
~	~	Daniel Bloomfield	
~	~	Jack Broadfoot	
~	~	Tracy Callen	
~	~	Caroline Chau	
~	~	Jeng Heng Ching	
~	~	Heather Cuell	
~	~	Alisha Damani	
~	~	Charlotte Davies	
~	~	Tracey Dobson	
~	~	Sarah Ellis	
~	~	Wendy Golding	
~	~	Mya Gyi	
~	~	Jennifer Hale	
~	~	Dominic Hodgson	
~	~	Chloe Holden	
~	~	Joni Howells	
~	~	Eleanor Jones	
~	~	Robert Keating	
~	~	Kudingila Madhava	
~	~	Nataliya Martynyuk	
~	~	Lorna Meadows	
~	~	Badrriyya Mohamedali	
~	~	Yoodhvir Nagar	
~	~	Mark Noble	
~	~	Mila Roca	
~	~	Megan Rowley	
~	~	Wendy Stacey	

INVESTIGATORS AND COLLABORATORS: SITE STAFF
Staff on site delegation logs

City	Care_Site	Person_Name	Site_PI
~	~	Anna Stephenson	
~	~	Azarel Virgo	
~	~	Mary Wands	
~	~	Catrin Watkinson	
~	~	Alice White	
~	~	Robert Williams	
Preston, UK	Royal Preston Hospital	Alison Birtle	PI
~	~	Natalie Charnley	Co-I
~	~	Nicola Flaum	Co-I
~	~	Christina Hague	Co-I
~	~	Duleer Majeed	Co-I
~	~	Omi Parikh	Co-I
~	~	Sophie Raby	Co-I
~	~	Jose Rico	Co-I
~	~	Yee Pei Song	Co-I
~	~	Marcus Wise	Co-I
~	~	Amanda Alty	
~	~	Nafisa Arden	
~	~	Mandy Armstrong	
~	~	Andrea Ashton	
~	~	Katherine Ashton	
~	~	Hazel Aston	
~	~	David Barber	
~	~	Margaret Brunton	
~	~	Shelia Calvert	
~	~	Claire Corless	
~	~	Stephanie Cornthwaite	
~	~	William Croxford	
~	~	Sharon Curran	
~	~	Falalu Danwata	
~	~	Rose Ellard	
~	~	Davide Garau	
~	~	Cassandra Gleeson	

INVESTIGATORS AND COLLABORATORS: SITE STAFF

Staff on site delegation logs

City	Care_Site	Person_Name	Site_PI
~	~	Shahzad Gul	
~	~	Caroline Hatch	
~	~	Billy Hefferon	
~	~	Claire Hennigan	
~	~	Louise Hough	
~	~	Haiyan Huang	
~	~	Deepsi Khatiwada	
~	~	Patricia Knight	
~	~	Anna Macpherson	
~	~	Andrew Martyniak	
~	~	Dominic Mounsey	
~	~	Tanmay Mukhopadhyay	
~	~	Hemant Patel	
~	~	Hazel Preston	
~	~	Sarah Preston	
~	~	Christina Robinson	
~	~	Roy Shentall	
~	~	Norma Sidek	
~	~	Win Soe	
~	~	Martin Swinton	
~	~	Catherine Thompson	
~	~	Nina Vekaria	
~	~	Catherine Walmsley	
~	~	Rebecca Wilby (nee Hall)	
~	~	Deborah Williamson	
Reading, UK	Royal Berkshire Hospital	Paul Rogers	PI
~	~	Osamah Al-Asadi	Co-I
~	~	Rowena Cazalet	Co-I
~	~	Rebecca Johnson	Co-I
~	~	Ali Abbas	
~	~	Abdolnasser Aminiraouf	
~	~	Jane Atkinson	
~	~	Gabrielle Ball	

INVESTIGATORS AND COLLABORATORS: SITE STAFF

Staff on site delegation logs

City	Care_Site	Person_Name	Site_PI
~	~	Gagan Bhatnagar	
~	~	Richard B Brown	
~	~	Debbie Cartwright	
~	~	James Church	
~	~	Claire Connolly	
~	~	Kristy Coomber	
~	~	Nicola Dallas	
~	~	Catherine Deytrikh-Smith	
~	~	Juliette Dye	
~	~	Shawn Ellis	
~	~	Fiona Everson	
~	~	Suzanne Foxwell	
~	~	Maxine Gauntlett	
~	~	Anna Gillham	
~	~	Sanita Gurm	
~	~	Royda Hadi	
~	~	Silke Hahnewald	
~	~	Jo Hand	
~	~	Elizabeth Haydon	
~	~	Kirsty Horwood	
~	~	Allison Hunt	
~	~	Sian James	
~	~	Phillipa Johnstone	
~	~	Robert Jones	
~	~	Thomas Kindley	
~	~	Wioletta Kowalczyk-Williams	
~	~	Christina Lewis	
~	~	Geraldine Mason	
~	~	Sean O'Cathail	
~	~	Helen O'Donnell	
~	~	Omotola Ogunnigbo	
~	~	Tolu Okeke	
~	~	Pooja Pabari	

INVESTIGATORS AND COLLABORATORS: SITE STAFF
Staff on site delegation logs

City	Care_Site	Person_Name	Site_PI
~	~	Stephen Parr	
~	~	Kate Preston	
~	~	Helen Purdon	
~	~	Norma Shields	
~	~	Georges Sinclair	
~	~	Emma Vowell	
~	~	Phillip Webb	
~	~	Simon Wyatt	
~	~	Andreia da Cruz	
Redditch, UK	Alexandra Hospital	Lisa Capaldi	PI
~	~	Mujtaba Syed-Khaja	Co-I
~	~	Maggie Brown	
~	~	Stephanie Cook	
~	~	Jonathan Davies	
~	~	Joanna Hamilton	
~	~	Alison Harrison	
~	~	Hayley Hodson	
~	~	Jeanette Knapp	
~	~	Bartlomeij Kurec	
~	~	Asha Sivapalasuntharam	
~	~	Helen Tranter	
~	~	Jennifer Young	
Redhill, UK	East Surrey Hospital	Eva Letalova	
Romford, UK	Oldchurch Hospital	Neil Fisher	
Romford, UK	Queen's Hospital (Romford)	Kathryn Tarver	PI
~	~	Stephanie Gibbs	Ex-PI
~	~	Amani Chowdhury	
~	~	Dalisay Domingo	
~	~	Parveen Dugh	
~	~	Revanth Jannapureddy	
~	~	Mohammed Rashid Khan	
~	~	Helen Mackenzie	
~	~	Tina Mills-Baldock	

INVESTIGATORS AND COLLABORATORS: SITE STAFF
Staff on site delegation logs

City	Care_Site	Person_Name	Site_PI
~	~	Simerjyot Mudhar	
~	~	Samuel Mugari	
~	~	Neale O'Brien	
~	~	Ana-Marie Pena-Remorin	
~	~	Yousaf Razzak	
~	~	Jonathon Shamash	
~	~	Ramachandran Subramaniam	
Runcorn, UK	Halton Hospital	Ian Allen	
~	~	Duncan Knowles	
~	~	Carrie Lowthian	
~	~	Rebecca Madew (nee Tinker)	
~	~	Nemonie Marriott	
~	~	Andrea Young	
Salford, UK	Salford Royal Hospital	Noel Clarke	PI
~	~	Tony Elliott	Co-I
~	~	Euan Green	Co-I
~	~	Maurice Lau	Co-I
~	~	Anna Tran	Co-I
~	~	Rachael Allen	
~	~	Angela Ashton	
~	~	Chris Betts	
~	~	Nicholas Boxall	
~	~	Richard Cowan	
~	~	Soney Dharmaprasad	
~	~	Claire Dickson	
~	~	Claire Duncan (nee Keatley)	
~	~	Christine Farnworth	
~	~	Helen Farrell	
~	~	Kathryn Fry	
~	~	Siny George	
~	~	Kay Goulden	
~	~	Samia Hanif	
~	~	Ashley Harris	

INVESTIGATORS AND COLLABORATORS: SITE STAFF

Staff on site delegation logs

City	Care_Site	Person_Name	Site_PI
~	~	Leah Harter	
~	~	Joanne Henry	
~	~	Jason Howard	
~	~	Jean Jellicoe	
~	~	Richard Jones	
~	~	Elina Jose	
~	~	Claire Keatley	
~	~	Sarah Kirk	
~	~	Kieran O'Flynn	
~	~	Anne-Marie Peers	
~	~	Danielle Platt	
~	~	Catherine Redshaw	
~	~	David Shackley	
~	~	Mark Stapleton	
~	~	Melanie Taylor	
~	~	Vicky Thomas	
~	~	Cellins Vinod	
~	~	Oliver Wadsworth	
~	~	Jill Youd	
Scarborough, UK	Scarborough General Hospital	Mohammad Muneeb Khan	PI
~	~	Mohan Hingorani	Ex-PI
~	~	Simon Hawkyard	Co-I
~	~	Khaliq Rehman	Co-I
~	~	Alison Ames	
~	~	Donna Anderson	
~	~	Lisa Armitage	
~	~	Fizzah Asif	
~	~	Laura Barman	
~	~	Chloe Box	
~	~	Kevin Brame	
~	~	Pippa Carlton-Rylance	
~	~	Courtney Cole	
~	~	Poppy Cottrell-Howe	

INVESTIGATORS AND COLLABORATORS: SITE STAFF

Staff on site delegation logs

City	Care_Site	Person_Name	Site_PI
~	~	Cheryl Donne	
~	~	Nabil El-Mahdawi	
~	~	Arran Fletcher	
~	~	Joanne Fletcher	
~	~	Vic Gacek	
~	~	Tracey Hawkes	
~	~	Sacha Honour	
~	~	Diana Ionita	
~	~	Adnan Kabir	
~	~	Sarah Kent	
~	~	Richard Khafagy	
~	~	Janine Mallinson	
~	~	Russell Morgan	
~	~	Tania Neale	
~	~	Polly Needs	
~	~	Anne Nunn	
~	~	Carol Popplestone	
~	~	Ian Renwick	
~	~	Andrew Robertson	
~	~	Alicia Rodgers	
~	~	Abigail Rowbotham	
~	~	Jacqui Smith	
~	~	Rachel Spooner	
~	~	Amie Stewart	
~	~	Jane Taylor	
~	~	Alison Turnbull	
~	~	Paul Wood	
Sheffield, UK	Weston Park Hospital	Carmel Pezaro	PI
~	~	Omar Din	Co-I
~	~	Shabbir Rawther	Co-I
~	~	Virgil Sivoglo	Co-I
~	~	Jess Aldred	
~	~	Cyper Allan	

INVESTIGATORS AND COLLABORATORS: SITE STAFF
Staff on site delegation logs

City	Care_Site	Person_Name	Site_PI
~	~	Mymoona Alzouebi	
~	~	Ryan Asher	
~	~	Lynne Ashmore	
~	~	Lucy Birch	
~	~	Joanne Bird	
~	~	Susan Bishop	
~	~	Katie Bowen	
~	~	Janet Brown	
~	~	Richard Brown	
~	~	Sarah Brown	
~	~	Roger Burkinshaw	
~	~	Chloe Clegg	
~	~	Gemma Dale	
~	~	Tathagata Das	
~	~	Julia Disney	
~	~	Linda Evans	
~	~	Catherine Ferguson	
~	~	Leigh Fiorentino	
~	~	Alexandra Firth	
~	~	Steffy George	
~	~	Kate Gibbins	
~	~	Elizabeth Hodgkinson	
~	~	Mark Holliday	
~	~	Marion Hutchinson	
~	~	Peter Kirkbride	
~	~	James Lester	
~	~	Rebecca Lomax-Allen	
~	~	Eileen Marsh	
~	~	John Martindale	
~	~	Jessica Medcalf	
~	~	Louise Murray	
~	~	Prashanth Sanganalmath	
~	~	Ruta Segamogaite	

INVESTIGATORS AND COLLABORATORS: SITE STAFF

Staff on site delegation logs

City	Care_Site	Person_Name	Site_PI
~	~	Roseleen Sheehan	
~	~	Janine Smedley (nee McCabe)	
~	~	Lucy Smith	
~	~	Anne Smythe	
~	~	Catherine Spalton	
~	~	Rachel Toes	
~	~	Lucy Walkington	
~	~	Katherine Williams	
~	~	Kim Wood	
Shrewsbury, UK	Royal Shrewsbury Hospital	Narayanan Srihari	PI
~	~	Ravi Prashant	Co-I
~	~	Riquella Abbott	
~	~	Huzeifa Abdel	
~	~	Marion Adams	
~	~	Beshar Allos	
~	~	Shazad Aslam	
~	~	Mandy Bates	
~	~	Erica Beaumont	
~	~	Mandy Beekes	
~	~	James Best	
~	~	Rajanee Bhana	
~	~	Lisa Capaldi	
~	~	Danielle Childs	
~	~	Lisa Evans	
~	~	Gill Ferguson	
~	~	Huzeifa Gadir	
~	~	Qamar Ghafoor	
~	~	Nicola Henderson	
~	~	Hayley Hughes	
~	~	Nicola Jones	
~	~	Sanal Jose	
~	~	Siobhan Kilbane	
~	~	Verity King	

INVESTIGATORS AND COLLABORATORS: SITE STAFF

Staff on site delegation logs

City	Care_Site	Person_Name	Site_PI
~	~	Sunita Kurian-Downer	
~	~	Jenny Lakin	
~	~	Anna Law	
~	~	Gemma Lee	
~	~	Michael Leigh	
~	~	Rachel McGregor	
~	~	Elena Michael	
~	~	Helen Moore	
~	~	Emma Neeves	
~	~	Karen Nicholas	
~	~	Catherine Orrell	
~	~	Lucy Pennant	
~	~	Craig Pickering	
~	~	Suzanne Pope	
~	~	Sally Potts	
~	~	Renee Poulsom	
~	~	Aitzaz Qaisar	
~	~	Catherine Santiago	
~	~	Gemma Searle	
~	~	Jenny Simm	
~	~	Harpreet Singh	
~	~	Sandra Smith	
~	~	Andy Taylor	
~	~	Alison Tilley	
~	~	Mathai Varghese	
~	~	Natasha Wallbank	
~	~	Emma Weaver	
~	~	Rebecca Wilcox	
~	~	Sundus Yahya	
~	~	Angela Yeomans	
~	~	Abel Zachariah	
South Shields, UK	South Tyneside District Hospital	Ashraf Azzabi	PI
~	~	Amy Burns	

INVESTIGATORS AND COLLABORATORS: SITE STAFF

Staff on site delegation logs

City	Care_Site	Person_Name	Site_PI
~	~	Maxine Goldsbrough	
~	~	Sally Hall	
~	~	Judith Moore	
~	~	Sue Morrison	
~	~	Ruth Tindle	
Southampton, UK	Southampton General Hospital	Simon Crabb	PI
~	~	Emma Brown	Co-I
~	~	Tessa Greenhalgh	Co-I
~	~	Chloe Holden	Co-I
~	~	Harish Reddy	Co-I
~	~	Caroline Andrews	
~	~	Liane Armstrong	
~	~	Holly Burton	
~	~	Nikki Carney	
~	~	Chris Coyle	
~	~	Kirsty Cumming	
~	~	Lucy Elswood	
~	~	Archana Gadve	
~	~	Julie Gwilt	
~	~	Annelise Haskell	
~	~	Catherine Heath	
~	~	Julie Kennedy	
~	~	Donna Kimber	
~	~	Yanli Li	
~	~	Maureen McAuley	
~	~	Victoria McFarlane	
~	~	Graham Mead	
~	~	Carolyn Mitchell	
~	~	Fabiola Morales-Azofra	
~	~	Susan Morton	
~	~	Carina Mundy	
~	~	Oyeleye Oyebola	
~	~	Nikki Prewitt	

INVESTIGATORS AND COLLABORATORS: SITE STAFF
Staff on site delegation logs

City	Care_Site	Person_Name	Site_PI
~	~	Leanne Reader	
~	~	Rebecca Rice	
~	~	Adele Ruiz	
~	~	Lorraine Street	
~	~	Sau-Mon Tsang	
~	~	Shauna Wakefield	
~	~	Matthew Wheater	
~	~	Aneta Zahorska	
Southport, UK	Southport and Formby District General Hospital	Manal Alameddine	PI
~	~	Neeraj Bhalla	Ex-PI
~	~	Dawn Barker	
~	~	Margaret Brunton	
~	~	Lisa Dobson (nee Child)	
~	~	Chinnamani Eswar	
~	~	Ken Gardner	
~	~	Julie Griffiths	
~	~	Laurie Lomax	
~	~	Marie McBride	
~	~	Teresa Monahan	
~	~	Heidi Moran	
~	~	Anna Morris	
~	~	Sandra Robinson	
~	~	Linda Schinkel	
~	~	Angela Scullion	
~	~	Asha Sivapalasuntharam	
~	~	Ann Wearing	
St Leonards-on-Sea, UK	Conquest Hospital	Caroline Manetta	PI
~	~	Atikah Ayaz	
~	~	Theresa Baumber	
~	~	Sharon Beesley	
~	~	Sarah Draper	
~	~	Steve Garnett	
~	~	Duncan Gilbert	

INVESTIGATORS AND COLLABORATORS: SITE STAFF
Staff on site delegation logs

City	Care_Site	Person_Name	Site_PI
~	~	Sarah Goodwin	
~	~	Joanna Howard	
~	~	Kay Jones-Skipper	
~	~	Kathryn Lees	
~	~	Lauren McCrisken	
~	~	Fiona McKinna	
~	~	Roger Plail	
~	~	Gail Pottinger	
~	~	Aspasia Soultati	
~	~	Jo-Anne Taylor	
~	~	Mark Whitfield	
Stevenage, UK	Lister Hospital	Robert Hughes	PI
~	~	Stephen Almond	
~	~	Anna Anosova	
~	~	Alkhaldi Ashraf	
~	~	Mawuelikem Assoku	
~	~	Corinne Bradshaw	
~	~	Clare Collins	
~	~	Sura Dabbagh	
~	~	Martin Ebon	
~	~	Jemma Gilmore	
~	~	Sunita Gohil	
~	~	Vicky Hills	
~	~	Rachel Low	
~	~	Leena Mukherjee	
~	~	Sayyida Nembhard	
~	~	Nikhil Oommen	
~	~	Katie Poole	
~	~	Natalie Rahim	
~	~	Anita Rana	
~	~	Roisin Schimmel	
~	~	Jonathan Towler	
~	~	Alice Valle	

INVESTIGATORS AND COLLABORATORS: SITE STAFF
Staff on site delegation logs

City	Care_Site	Person_Name	Site_PI
~	~	David Ward	
~	~	Steven Watkins	
~	~	Elen Witness	
~	~	David Woolf	
Stockport, UK	Stepping Hill Hospital	John Logue	PI
~	~	Adebanji Adeyoju	
~	~	Wasim Akhtar	
~	~	Carmel Anandadas	
~	~	Eleanor Anscombe	
~	~	Miriam Avery	
~	~	Paul Berry	
~	~	Aelens Brauckman	
~	~	Stephen Bromage	
~	~	Richard Brough	
~	~	Louise Brown	
~	~	Stephen CW Brown	
~	~	Jean Cheetham	
~	~	Pat Clitheroe	
~	~	Tracie Cocks	
~	~	Gerald Collins	
~	~	Sarah Connolly nee McKenna	
~	~	Sam Corcoran	
~	~	Catherine Coyle	
~	~	Catherine Fox	
~	~	Christina Gilmour	
~	~	Emma Goodwin	
~	~	Susan Graham	
~	~	Umi Hatimy	
~	~	Helen Haydock	
~	~	Nicola Hermitage	
~	~	Emma Hewitt	
~	~	Sheila Hodgkinson	
~	~	Susan Hopkins	

INVESTIGATORS AND COLLABORATORS: SITE STAFF
Staff on site delegation logs

City	Care_Site	Person_Name	Site_PI
~	~	Apurna Jegannathen	
~	~	Zoe Jordan	
~	~	Anna Kellingray	
~	~	Alissa Kent	
~	~	John Kilmartin	
~	~	Magda Kujawa	
~	~	Abigail Mackley	
~	~	Patrick O'Reilly	
~	~	Oluwademilade Odewumi	
~	~	Lucy Orrell	
~	~	Abigail Pemberton	
~	~	Benjamin Ralphs	
~	~	Mkyla Reilly	
~	~	David Ross	
~	~	Andrew Sinclair	
~	~	Emma Taylor	
~	~	Jill Taylor	
~	~	Satish Venkateshan	
~	~	Katrina Wade	
~	~	Jonathan Wong	
~	~	Donald van Welsenes	
Stockton-on-Tees, UK	North Tees General Hospital	Devadasan Shakespeare	
Stockton-on-Tees, UK	University Hospital of North Tees	Darren Leaning	PI
~	~	Alison Chilvers	
~	~	Helen Dunn (nee Carey)	
~	~	Emma Jameson	
~	~	Hyder Latif	
~	~	Abdul Mian	
~	~	Victor Palit	
~	~	Moira Percival	
~	~	Sarah Pitcairn	
~	~	Leigh Pollard	
~	~	Lynda Poole	

INVESTIGATORS AND COLLABORATORS: SITE STAFF
Staff on site delegation logs

City	Care_Site	Person_Name	Site_PI
~	~	Pam Race	
~	~	Devadasan Shakespeare	
~	~	Andrew Sigsworth	
~	~	Helen Wardle (nee Wilson)	
~	~	Bill Wetherill	
Stoke-on-Trent, UK	Royal Stoke University Hospital	Salil Vengalil	PI
~	~	Fawzi Adab	
~	~	Eden Ball	
~	~	Rajanee Bhana	
~	~	Isabel Breeze	
~	~	Marion Evans	
~	~	Grace Gough	
~	~	Robert Green	
~	~	Emma Jackson	
~	~	Christopher Luscombe	
~	~	Alison Myatt	
~	~	Katrina Parkinson	
~	~	Angela Peake	
~	~	Sharon Rollison	
~	~	Elizabeth Sellars	
~	~	Rowena Smith	
~	~	Julie Storer	
~	~	Alison Tute	
~	~	Liberty Verueco	
~	~	Angela Ward	
~	~	Elizabeth Williamson	
Sunderland, UK	Sunderland Royal Hospital	Ashraf Azzabi	PI
~	~	Rachel Pearson	Co-I
~	~	Ian Pedley	Co-I
~	~	Kathryn Wright	Co-I
~	~	Rod Beard	
~	~	Stephen Butler	
~	~	Jane Cole	

INVESTIGATORS AND COLLABORATORS: SITE STAFF
Staff on site delegation logs

City	Care_Site	Person_Name	Site_PI
~	~	Michelle Edwards	
~	~	Terri Haldane	
~	~	Christine Harle	
~	~	Amanda Howey	
~	~	Vivienne Hullock	
~	~	Shahid Iqbal	
~	~	Stephen Laybourne	
~	~	Paula Newton	
~	~	Julia Scott	
~	~	Karen Shield	
~	~	Fiona Wakinshaw	
Sutton Coldfield, UK	Good Hope Hospital	Daniel Ford	PI
~	~	Mark O'Beirn	Co-I
~	~	Kamaldeep Ajimal	
~	~	Shobit Baijal	
~	~	Chen Bartlett	
~	~	Ellen Drew	
~	~	Steve Hay	
~	~	Lubna Khan	
~	~	Alison Maidment	
~	~	Beena Mistry	
~	~	Katy Moore	
~	~	Rachael O'Beney	
~	~	Janet Prentice	
~	~	Sarah Rogers	
~	~	Sundip Sohanpal	
~	~	Lorna Swaddle	
~	~	Helen Taylor	
~	~	Helen Thomas	
~	~	James Whitehouse	
Sutton, UK	Royal Marsden Hospital (Sutton)	Chris Parker	PI
~	~	Douglas Brand	Co-I
~	~	Angela Pathmanathan	Co-I

INVESTIGATORS AND COLLABORATORS: SITE STAFF

Staff on site delegation logs

City	Care_Site	Person_Name	Site_PI
~	~	Nora Sundahl	Co-I
~	~	Fatima Ahmed	
~	~	Rookmeen Alighan	
~	~	Eva Batovska	
~	~	Martha Bullimore	
~	~	Sue Cromarty	
~	~	Claire Crowley	
~	~	Kirsty Cuthbertson	
~	~	David Dearnaley	
~	~	Rosalind Eeles	
~	~	Lucy Featherstone	
~	~	Janine Flohr	
~	~	Amir El Ghazal	
~	~	Zaynah Gurreebun	
~	~	Laura Hennelly	
~	~	Adham Hijab	
~	~	Alan Horwich	
~	~	Robert Huddart	
~	~	Nick Hunnings	
~	~	Tiaan Jacobs	
~	~	Bernadette Johnson	
~	~	Kelly Jones	
~	~	Vincent Khoo	
~	~	Susan Lalondrelle	
~	~	Alexander Macnab	
~	~	Chloe McCormack	
~	~	Gerard McVey	
~	~	Sally Moore	
~	~	Annette Musallam	
~	~	Jenni Parmar	
~	~	Ray Shepherd	
~	~	Victoria Sjolin	
~	~	Helen Stidwell	

INVESTIGATORS AND COLLABORATORS: SITE STAFF

Staff on site delegation logs

City	Care_Site	Person_Name	Site_PI
~	~	Alex Tan	
~	~	Alison Tree	
~	~	Ruth Woode-Amissah	
Sutton-in-Ashfield, UK	King's Mill Hospital	Georgina Walker	PI
~	~	Daniel Saunders	Ex-PI
~	~	Louise Brookes	Co-I
~	~	Benjamin Masters	Co-I
~	~	Sadia Abdullah	
~	~	Samantha Boam	
~	~	Andrew Brocklehurst	
~	~	Jamie-Rae Burgoyne	
~	~	Eliot Chadwick	
~	~	Muhammad Gill	
~	~	Robert Goldspring	
~	~	Steve Haigh	
~	~	Shila Hamzepur	
~	~	Rebecca Holmes	
~	~	Lauren Jones	
~	~	Jun Lim	
~	~	Wayne Lovegrove	
~	~	Samantha March	
~	~	Victoria Moore	
~	~	Dominic Nash	
~	~	Michael Ocathail	
~	~	Linda Otter	
~	~	Andrea Palfreman	
~	~	James Price	
~	~	Lisa Rahn	
~	~	Wai Hou Sam	
~	~	Terri-Ann Sewell	
~	~	Sarah Shelton	
~	~	Katie Slack	
~	~	Fiona Smith	

INVESTIGATORS AND COLLABORATORS: SITE STAFF
Staff on site delegation logs

City	Care_Site	Person_Name	Site_PI
~	~	Susan Smith	
~	~	Sarah Taylor	
~	~	Elena Umbrarescu	
~	~	Lynne Wade	
~	~	Margaret Wheatley	
~	~	Inez Wynter	
Swansea, UK	Singleton Hospital	Ahmed Shaheen	PI
~	~	Rhian Davies	Co-I
~	~	Helen Fitzgerald	Co-I
~	~	Nia Jackson	Co-I
~	~	Sheena Lam	Co-I
~	~	Aijaz Lone	Co-I
~	~	Wael Mohamed	Co-I
~	~	Mau-Don Phan	Co-I
~	~	Fiona Williams	Co-I
~	~	Carl Ackland	
~	~	Russell Banner	
~	~	Gianfilippo Bertelli	
~	~	Lynne Breeze-Jones	
~	~	David Brown	
~	~	Jayne Caparros	
~	~	Helen Cheley	
~	~	Karen Chesters	
~	~	Amanda Cook	
~	~	Emma Dangerfield	
~	~	Nicola Davies	
~	~	Lisa Ellis	
~	~	Elizabeth Evans	
~	~	Stuart Evans	
~	~	Tracey Ford	
~	~	Alex Franklin	
~	~	Ricky Fraser	
~	~	Lorraine Gammon	

INVESTIGATORS AND COLLABORATORS: SITE STAFF
Staff on site delegation logs

City	Care_Site	Person_Name	Site_PI
~	~	Sharath Gangadhara	
~	~	Judith Gooding	
~	~	Sarah Gwynne	
~	~	Emily Harris (n. Marchant)	
~	~	Amanda Jackson	
~	~	Chelsea Jenkins	
~	~	Maria Johnstone	
~	~	Gillian Jones	
~	~	Lewis Jones	
~	~	Ashok Kumar	
~	~	Satish Kumar	
~	~	Donna Lear	
~	~	Nicola Lemon	
~	~	Jason Lester	
~	~	James Morgan	
~	~	Gillian Palmer	
~	~	Angharad Phillips	
~	~	Brian Phillips	
~	~	Karen Phillips	
~	~	Susie Pitcher	
~	~	Gail Povey	
~	~	Euan Pratt	
~	~	Delia Pudney	
~	~	Leanne Quinn	
~	~	Amy Quinton	
~	~	Alex Richards	
~	~	Mair Roberts	
~	~	Mark Rogers	
~	~	Michelle Romano	
~	~	N Sindgi	
~	~	Alison Stretch	
~	~	Ellen Tait	
~	~	Katie Tanner	

INVESTIGATORS AND COLLABORATORS: SITE STAFF
Staff on site delegation logs

City	Care_Site	Person_Name	Site_PI
~	~	Anne Thomas	
~	~	Nia Viney	
~	~	John Wagstaff	
~	~	Gillian Willetts	
~	~	Dawn Withers	
~	~	Naomi Woods	
~	~	Charlotte Young	
Swindon, UK	Great Western Hospital	Omar Khan	PI
~	~	Gerard Andrade	
~	~	Aiste Baltramaityte	
~	~	Rebecca Belcher	
~	~	Graham Brown	
~	~	Christopher Clarke	
~	~	David J Cole	
~	~	Amanda Colston	
~	~	Sarah Cotton	
~	~	Nicola Cowling	
~	~	Shiroma De Silva-Minor	
~	~	Jan Dodge	
~	~	Fahad Fazal	
~	~	Victoria Gibson	
~	~	Sarah Grayland	
~	~	Lesley Haxton	
~	~	Ellie Hewitt	
~	~	Esme Hill	
~	~	Raj Jampana	
~	~	Ania Jones	
~	~	Jean Kordula	
~	~	Lynsey Kyeremeh	
~	~	Donna Lake	
~	~	Jonathan Lewis	
~	~	Mike Lewis	
~	~	Catherine Lewis Clarke	

INVESTIGATORS AND COLLABORATORS: SITE STAFF
Staff on site delegation logs

City	Care_Site	Person_Name	Site_PI
~	~	Sarah Long	
~	~	Dorothe Maramak	
~	~	Dorota Marciniak	
~	~	Laura McCafferty	
~	~	Sue Meakin	
~	~	Aruna Medisetti	
~	~	Rachel Messenger	
~	~	Chanelle Meyer	
~	~	David Newell	
~	~	Tim Owen	
~	~	Debbie Palmer	
~	~	Cerila Parajes	
~	~	Sally-Ann Parkin (nee) Lee	
~	~	Ronak Patel	
~	~	Suzannah Pegler	
~	~	Caroline Pensotti	
~	~	Tracey Sargent	
~	~	Deborah Scott	
~	~	Karen Smith	
~	~	Ellen Starling	
~	~	Joseph Stevens	
~	~	Emma Wakefield	
~	~	Helen Winter	
~	~	Vivian Zinyemba	
Taunton, UK	Musgrove Park Hospital	Emma Gray	PI
~	~	John Graham	Ex-PI
~	~	Nicola Cox	Co-I
~	~	Mohini Varughese	Co-I
~	~	John Allinson-Smith	
~	~	Jan Ashcroft	
~	~	Nita Beacham	
~	~	Hannah Berry	
~	~	Ian Bodger	

INVESTIGATORS AND COLLABORATORS: SITE STAFF
Staff on site delegation logs

City	Care_Site	Person_Name	Site_PI
~	~	Joanne Botten	
~	~	Lisa Bowern	
~	~	Darren Brady	
~	~	Christina Branfield	
~	~	Rebecca Brown	
~	~	Clair Brunner	
~	~	Richard Burgess	
~	~	Alison Chedham	
~	~	Rachel Coe	
~	~	Hayley Cornall	
~	~	Susan Crouch	
~	~	Nicola Cutmore	
~	~	Rebecca Denslow	
~	~	Jarrod Dunn	
~	~	Michelle Farrar	
~	~	Abby Farzaneh	
~	~	Simon Goldsworthy	
~	~	Fiona Goodchild	
~	~	Amanda Groves	
~	~	Clair Hinton	
~	~	Lucy Howell-Drewett	
~	~	Joseph Jelski	
~	~	Odunayo Kalejaiye	
~	~	Joan Kemp	
~	~	Manjusha Keni	
~	~	Catherine Lane	
~	~	Lynn Leat	
~	~	Fen Lewen	
~	~	Angela Locke	
~	~	Ruaraidh MacDonagh	
~	~	Sue Mahoney	
~	~	Anna Masamba	
~	~	Judith Mathie	

INVESTIGATORS AND COLLABORATORS: SITE STAFF
Staff on site delegation logs

City	Care_Site	Person_Name	Site_PI
~	~	Sara Myers	
~	~	Sayyida Nembhard	
~	~	Samantha Northover	
~	~	Corinne Pawley	
~	~	George Plataniotis	
~	~	Ceri Poyntz-wright	
~	~	Rebecca Purnell	
~	~	Gihan Ratnayake	
~	~	Guillermo Reina-Ruiz	
~	~	Joanne Rogers	
~	~	Joy Rowe	
~	~	Tamlyn Russell	
~	~	Amy Sawyer	
~	~	Alison Snell	
~	~	Claire Sowerby	
~	~	Luke Stephens	
~	~	Moira Tait	
~	~	Karen Tanner	
~	~	Joanne Taylor	
~	~	Mary Tighe	
~	~	Rebecca Tucker	
~	~	Rebecca Twemlow	
~	~	Elena Umbrarescu	
~	~	Rebecca Wallbutton	
~	~	Joshua Woollven	
~	~	Jasmine Youens	
~	~	Robert Zorica	
Taunton, UK	Taunton and Somerset Hospital	Jan Ashcroft	
~	~	Jarrod Dunn	
~	~	Ruaraidh MacDonagh	
~	~	Judith Mathie	
~	~	Rebecca Tucker	
Torquay, UK	Torbay District General Hospital	Anna Lydon	PI

INVESTIGATORS AND COLLABORATORS: SITE STAFF

Staff on site delegation logs

City	Care_Site	Person_Name	Site_PI
~	~	Fiona Roberts	Co-I
~	~	Michele Allison	
~	~	Kenneth Almedilla	
~	~	Emmie Arbury	
~	~	Victoria Bell	
~	~	Martyn Blundell	
~	~	Lauren Blunt	
~	~	Jo Blurton	
~	~	Mark Brennan	
~	~	Catherine Brookman	
~	~	Shelley Chamberlain	
~	~	Melody Cross	
~	~	Donna Cuffe	
~	~	Stacey Davies	
~	~	Sue Forbes	
~	~	Angela Foulds	
~	~	Helen Greedus	
~	~	Andrew Harford-Brown	
~	~	Helen Kimber	
~	~	Magdi Kirollos	
~	~	Ingrid Koehler	
~	~	Sally Maddison	
~	~	Catherine Marshall	
~	~	Robert Mason	
~	~	Seamus McDermott	
~	~	Jorg Michels	
~	~	Lyn Micklewright	
~	~	Amy Millington	
~	~	Sophie Norman	
~	~	Louise Paatz	
~	~	Janet Palmer	
~	~	Kirsty Pearce	
~	~	Christine Rawlings	

INVESTIGATORS AND COLLABORATORS: SITE STAFF

Staff on site delegation logs

City	Care_Site	Person_Name	Site_PI
~	~	Sarah Rees	
~	~	Rajaguru Srinivasan	
~	~	Lorraine Thornton	
~	~	Elaine Vandecandalaere	
~	~	Amanda Vian	
~	~	Beverley Watkins	
~	~	Erica Watts	
~	~	Sally Wells	
~	~	Linda Welsh	
~	~	Sarah Wright	
Warrington, UK	Warrington Hospital	Isabel Syndikus	PI
~	~	Shaun Tolan	Co-I
~	~	Lucy Berresford	
~	~	Lisa Dobson (nee Child)	
~	~	Jade Keenan	
~	~	Duncan Knowles	
~	~	Lisa Lee	
~	~	Carrie Lowthian	
~	~	Rebecca Madew (nee Tinker)	
~	~	Nemonie Marriott	
~	~	Philip Reynolds	
~	~	Sandra Robinson	
~	~	Andrea Young	
Warwick, UK	Warwick Hospital	Andrew Chan	PI
~	~	Maggie Brown	
~	~	Judith Chettle	
~	~	Jacqui Harris	
~	~	Lyn Hartwell	
~	~	Julia Jones	
~	~	Linda Maher	
~	~	Helen Millage	
~	~	Emily Noonan	
~	~	Eilish O'Neill	

INVESTIGATORS AND COLLABORATORS: SITE STAFF
Staff on site delegation logs

City	Care_Site	Person_Name	Site_PI
~	~	Jackie Sears	
~	~	Lucy Shafiq	
~	~	Andrew Stockdale	
~	~	Donna Walsh	
~	~	Frances Walsh	
~	~	Jo Williams	
Westcliff on Sea, UK	Southend University Hospital	Imtiaz Ahmed	PI
~	~	Abby Cyriac	Co-I
~	~	David Tsang	Co-I
~	~	Sue Bowman	
~	~	Kelly Buckhorn	
~	~	Thomas Carr	
~	~	Olivia Chan	
~	~	Stuart Chandler	
~	~	Lesley Cranfield	
~	~	Tracey Davies	
~	~	Terry Dowling	
~	~	Lesley Googe	
~	~	Kathryn Hawkesford	
~	~	Andrew Ho	
~	~	Ken Kennedy	
~	~	Joana Kyte	
~	~	Richard Lodge	
~	~	Tanatswa Mabhoyi	
~	~	Katrina Maitland	
~	~	Lesley Nichols	
~	~	Shanas Noor	
~	~	Ololade Omodunbi	
~	~	Sreekanth Palvai	
~	~	Meera Patel	
~	~	Jan Prejbisz	
~	~	Amdadur Rahman	
~	~	Usha Ravichandran	

INVESTIGATORS AND COLLABORATORS: SITE STAFF

Staff on site delegation logs

City	Care_Site	Person_Name	Site_PI
~	~	Sheila Reece	
~	~	Rachel Sadan	
~	~	Naveed Sarwar	
~	~	Ryan Wong	
~	~	Nuhu Yaroson	
Weston Super Mare, UK	Weston General Hospital	Serena Hilman	PI
~	~	Thomas Bird	Co-I
~	~	Tom Wells	Co-I
~	~	Kathy Beard	
~	~	Sandra Beech	
~	~	Debbie Coles	
~	~	Donna Cotterill	
~	~	Harvey Dymond	
~	~	Symeon Eleftheriadis	
~	~	Rajesh Gamare	
~	~	Denise Leighton-Price	
~	~	Hugh Lloyd-Jones	
~	~	Jennifer Maby	
~	~	Andrew McKendrick	
~	~	Kristina Owens	
~	~	Dave Pack	
~	~	Glenn Saunders	
~	~	Dawn Simmons	
~	~	Marjorie Tomlinson	
~	~	Rachel Warinton	
~	~	Susan Wilkinson	
Whitehaven, UK	West Cumberland Hospital	Fiona Douglas	PI
~	~	Anil Kumar	PI
~	~	Angela Birt	
~	~	Christopher Brewer	
~	~	Alan Denholm	
~	~	Charlotte Eyles	
~	~	Grace Fryer	

INVESTIGATORS AND COLLABORATORS: SITE STAFF
Staff on site delegation logs

City	Care_Site	Person_Name	Site_PI
~	~	Tim Marshalsea	
~	~	Patricia Nicholls	
~	~	Jonathan Nicoll	
~	~	Muhammad Rahman	
~	~	Norma Sidek	
~	~	Fiona Spence	
~	~	Jenna Wildey	
~	~	Beverley Wilkinson	
~	~	Joanne Wilkinson	
~	~	Fergus Young	
Wigan, UK	Royal Albert Edward Infirmary	Anna Tran	PI
~	~	Euan Green	Co-I
~	~	Steve Adejumo	
~	~	Julie Barnes	
~	~	David J Bell	
~	~	Jenny Bradshaw	
~	~	Jennifer Cannon	
~	~	Richard Cowan	
~	~	Louise Devereaux	
~	~	Alison Doran	
~	~	Sonia Evans	
~	~	Diane Forrest	
~	~	Elian Green	
~	~	Paul Higham	
~	~	Claire Hill	
~	~	Andrew Hudson	
~	~	Su Kim	
~	~	Sarah Kirk	
~	~	Andrew McPartlin	
~	~	Karen Moss	
~	~	Muthuswarmy Nagarajan	
~	~	Michael Parks	
~	~	Angela Power	

INVESTIGATORS AND COLLABORATORS: SITE STAFF

Staff on site delegation logs

City	Care_Site	Person_Name	Site_PI
~	~	Catherine Redshaw	
~	~	Tonia Louise Selby	
~	~	Dianna Thompson	
~	~	Zoe Trumper	
~	~	Marissa Walters	
Winchester, UK	Royal Hampshire County Hospital	Sangeeta Paisey	PI
~	~	Rao Vuyyuru	Co-I
~	~	Andrew Adamson	
~	~	Louise Beattie	
~	~	Julie Conti	
~	~	Victoria Corner	
~	~	Angela Firth	
~	~	Liz Happle	
~	~	Ina Hoad	
~	~	Lesley Hollister	
~	~	Abigail Hughes	
~	~	Lauriane Kerwood	
~	~	Carley Merritt	
~	~	Christina Narh	
~	~	Fasar Sarwar	
~	~	Jackie Smith	
~	~	Anna Song	
Wolverhampton, UK	New Cross Hospital	Ian Sayers	PI
~	~	Syed Abdullah Bukhari	Co-I
~	~	Amrita Solanki	Co-I
~	~	Amarpal Bains	
~	~	Ann Bentley	
~	~	Emily Carter	
~	~	Vanda Carter	
~	~	Mark Churn	
~	~	Peter Cooke	
~	~	Georgi Georgiev	
~	~	Anna Grant	

INVESTIGATORS AND COLLABORATORS: SITE STAFF

Staff on site delegation logs

City	Care_Site	Person_Name	Site_PI
~	~	Kay Hadlington	
~	~	Uttara Karnik	
~	~	Kelly Kauldhar	
~	~	Pek Keng-Koh	
~	~	Christine Kirk	
~	~	Claire Lomas	
~	~	Nataliya Martynyuk	
~	~	Joanne Mundy	
~	~	Renita Pawaroo	
~	~	Bajinder Rai	
~	~	Jason Rogers	
~	~	Sharon Rudge	
~	~	Gurminder Sahota	
~	~	Emma Sharman	
~	~	Debbie Spruce	
~	~	Arvind Tripathy	
~	~	Davina Warrender	
Worcester, UK	Worcestershire Royal Hospital	Lisa Capaldi	PI
~	~	Menna Fouda	Co-I
~	~	Kamalnayan Gupta	Co-I
~	~	Ayyaz Munawar	Co-I
~	~	Susan Anderson	
~	~	Khin Aye	
~	~	Dagmara Bak	
~	~	Jo Bowen	
~	~	Kristy Cleary	
~	~	Sue Davies	
~	~	Paul Flinders	
~	~	Janet Forkes	
~	~	Monica Gauntlett	
~	~	Alison Harrison	
~	~	Jennifer Healey-Mariano	
~	~	Hayley Hodson	

INVESTIGATORS AND COLLABORATORS: SITE STAFF
Staff on site delegation logs

City	Care_Site	Person_Name	Site_PI
~	~	Amanda Holdsworth	
~	~	Bartlomeij Kurec	
~	~	Zeeshaan Parvez	
~	~	Jayadevkumar Pawadshetti	
~	~	Heather Perry	
~	~	Patricia Rimell	
~	~	Alison Rosoman	
~	~	Asha Sivapalasuntharam	
~	~	Sally Stringer (pr. Davis)	
~	~	Jacob Taylor	
~	~	Helen Tranter	
~	~	Jayne Tyler	
~	~	Ann White	
~	~	Nicola Williams	
Worthing, UK	Worthing Hospital	Ashok Nikapota	PI
~	~	David Bloomfield	Ex-PI
~	~	Irvin Balagosa	
~	~	Stephanie Brown	
~	~	Fiona Castell	
~	~	Dawn Crowe (nee Hughes)	
~	~	Marian Flynn-Batham	
~	~	Linda Folkes	
~	~	Sarah Funnell	
~	~	Jeanette Gilbert	
~	~	Raquel Gomez-Marcos	
~	~	Celia Gonzalez	
~	~	Sarah House	
~	~	Helen Jones	
~	~	Sarah Kimber	
~	~	Jordi Margalef	
~	~	Leanne Mills	
~	~	Sally Moore	
~	~	George Plataniotis	

INVESTIGATORS AND COLLABORATORS: SITE STAFF
Staff on site delegation logs

City	Care_Site	Person_Name	Site_PI
~	~	Susan Rockall	
~	~	Matthew Smith	
~	~	Yvette Thirlwall	
~	~	Tan Tsawayo	
~	~	Nikki Turner	
~	~	Wendy Wood	
Yeovil, UK	Yeovil District Hospital	Tim Porter	PI
~	~	Sabri Ahmed	Co-I
~	~	Erica Beaumont	Co-I
~	~	Joanna Allison	
~	~	Zenaida Armstrong	
~	~	Claire Barron	
~	~	Nigel Beer	
~	~	Kate Beesley	
~	~	Debbie Cole	
~	~	Sunil Daryanani	
~	~	Sarah De Bruijn	
~	~	David Donaldson	
~	~	Tracey Duckett	
~	~	Shirley Fox	
~	~	Emma Gray	
~	~	Hassan Hameed	
~	~	Michelle Kotze	
~	~	David Laws	
~	~	Jess Perry	
~	~	Lucy Pippard	
~	~	Charlotte Reeves	
~	~	Kerry Rennie	
~	~	Geoffrey Sparrow	
~	~	Amanda Sweet	
~	~	Pamela White	
York, UK	University of York	Mark Schulpher	
York, UK	York District Hospital	Paul Brittain	

INVESTIGATORS AND COLLABORATORS: SITE STAFF
Staff on site delegation logs

City	Care_Site	Person_Name	Site_PI
~	~	Claire Brookes	
~	~	Flor Davies	
~	~	Cheryl Donne	
~	~	Mark Fearnley	
~	~	Sally Gilroy	
York, UK	York Teaching Hospital	Joji Joseph	PI
~	~	Ben Blake-James	Co-I
~	~	David Bottomley	Co-I
~	~	Russ Wilson	Co-I
~	~	Mark Aldous	
~	~	Ornella Belvedere	
~	~	Paul Brittain	
~	~	Claire Brookes	
~	~	Poppy Cottrell-Howe	
~	~	Tracey Dorey	
~	~	Mark Elliott	
~	~	Richard Evans	
~	~	Fereshteh Fallah	
~	~	Jayne Hammond	
~	~	Tom Hearfield	
~	~	Jo Ingham	
~	~	Laura Jeffery	
~	~	Kay Kell	
~	~	Prithivi Maheswaran	
~	~	Lisa Mole	
~	~	Daniel Petty	
~	~	Kate Ritchie	
~	~	Abigail Rowbotham	
~	~	Paula Strider	
~	~	Debora Twydell	
~	~	John Wightman	
~	~	Paul Wood	
~	~	Emily Worrall	

INVESTIGATORS AND COLLABORATORS: SITE STAFF

Staff on site delegation logs

City	Care_Site	Person_Name	Site_PI
Basel, CH	Universitätsspital Basel	Cyrill Rentsch	PI
~	~	Frank Stenner-Liewen	Co-I
~	~	Alexander Bachmann	
~	~	Nicole Ebinger	
~	~	Mana Farsad	
~	~	Eloise Kremer	
~	~	Simone Marini	
~	~	Kristina Muller	
~	~	Nicole Neumann	
~	~	N Ott	
~	~	Heike Puschel	
~	~	Christoph Rochlitz	
~	~	Bettina Seifest	
~	~	M Timmermann	
~	~	Stephen Wyler	
Bellinzona, CH	Istituto Oncologico della Svizzera Italiana	Ricardo Pereira Mestre	PI
~	~	Enrico Roggero	PI
~	~	Ngwa Che Azinwi	
~	~	Carolina De Almeida	
~	~	Maria Delgrande	
~	~	Vittoria Espeli	
~	~	Eloise Kremer	
~	~	Anna Llado	
~	~	Barbara Marongiu	
~	~	Michele Moro	
~	~	Gianfranco Pesce	
~	~	Sabine Van Den Bosch	
Berne, CH	Inselspital (University Hospital Berne)	Jörg Beyer	PI
~	~	Daniel Aebersold	
~	~	Anna-Katharina Herrmann	
~	~	Eloise Kremer	
~	~	Anselm Lafita	
~	~	Susan Meierhans	

INVESTIGATORS AND COLLABORATORS: SITE STAFF
Staff on site delegation logs

City	Care_Site	Person_Name	Site_PI
~	~	Timo Nannen	
~	~	Kathi Ochsner	
~	~	Simone Rimoldi	
~	~	Beat Roth	
~	~	George Thalmann	
~	~	Barbara Uhlmann	
~	~	Antje Ulrich	
~	~	Martin Waeber	
Biel, CH	Spitalzentrum Biel	Markus Borner	PI
~	~	Silvia Hanselmann	
~	~	Eloise Kremer	
~	~	Annette Winkler Vatter	
~	~	Béatrice Zimmerli Schwab	
Chur, CH	Kantonsspital Graubünden	Raeto Strebel	PI
~	~	Richard Cathomas	
~	~	Dirk Kienle	
~	~	Eloise Kremer	
~	~	Gabriela Manetsch	
~	~	M Mark	
~	~	Radmila Moudry	
~	~	Michael Schwitter	
~	~	Roger von Moos	
Lausanne, CH	Centre Hospitalier Universitaire Vaudois (CHUV)	Dominik Berthold	PI
~	~	May-Lucie Meyer	Co-I
~	~	Alice Abdallah	
~	~	Tewfik Abedlaziz	
~	~	Veronica Aedo	
~	~	Catherine Bender	
~	~	Galaad Bernard	
~	~	Yohan Boillat	
~	~	Floriane Bouilly	
~	~	Anna-Sophia Briod	
~	~	Carmen Castagna	

INVESTIGATORS AND COLLABORATORS: SITE STAFF
Staff on site delegation logs

City	Care_Site	Person_Name	Site_PI
~	~	Anabela Costa	
~	~	Antonella Diciolla	
~	~	Nathalie Divorne	
~	~	Akram Farhat	
~	~	Sabine Galland	
~	~	Sylvie Haudidier	
~	~	Fernanda Herrera	
~	~	Agnes Hiou Feige	
~	~	Nicole James Faresse	
~	~	Patrice Jichlinski	
~	~	Eloise Kremer	
~	~	Fabrice Lalubin	
~	~	Sofiya Latifyan	
~	~	Cynthia Leclerc	
~	~	Margaret McLauchlan	
~	~	Benangene Midez	
~	~	Sophia Murel	
~	~	Kaniana Ntanga Muambayi	
~	~	Rebecca Oppenheim	
~	~	Angela Orcurto	
~	~	Louis Parisod	
~	~	Claire Perrinjaquet	
~	~	Alexandra Rideau	
~	~	Hans-peter Roth	
~	~	Marc Schnety	
~	~	Cosette Schuler	
~	~	Norlene Silva	
~	~	Sandra Toffanin	
~	~	Geert Van Driessche	
~	~	Sophie Voegtlin	
~	~	Aline Voidey	
~	~	Celine Yerly	
~	~	Jean-Philippe Zurcher	

INVESTIGATORS AND COLLABORATORS: SITE STAFF

Staff on site delegation logs

City	Care_Site	Person_Name	Site_PI
Liestal, CH	Kantonsspital Liestal	Vanessa Fuhrer	
~	~	Eloise Kremer	
~	~	Andreas Lohri	
~	~	Simone Marini	
St Gallen, CH	Kantonsspital St Gallen	Daniel Engeler	PI
~	~	Aurelius Omlin	Co-I
~	~	Christian Rothermundt	Co-I
~	~	Christoph Schwab	Co-I
~	~	Dominik Abt	
~	~	Silke Gillessen	
~	~	Claudia Hormann	
~	~	Mannel Jungi	
~	~	Eloise Kremer	
~	~	Sigrid Patel	
~	~	Stefan Prensser	
~	~	Sibylle Schapper	
~	~	Karin Zuern	
~	~	Karin Zurn	
St. Gallen, CH	Klinik fur Urologie	Claudia Hormann	
~	~	Sibylle Schapper	
Winterthur, CH	Kantonsspital Winterthur	Hubert John	Ex-PI
~	~	Beatrice Brinkers	
~	~	Natalie Fisher	
~	~	Nicole Kradolfer	
~	~	Eloise Kremer	
~	~	Claudia Langer	
~	~	Muller	
~	~	Veronika Nagy	
~	~	Martina Pfitzner	
~	~	Miklos Pless	
~	~	Sabina Schacher	
~	~	SusyAnn Shaw	
~	~	Cindy Wanger	

INVESTIGATORS AND COLLABORATORS: SITE STAFF
Staff on site delegation logs

City	Care_Site	Person_Name	Site_PI
Zurich, CH	Hirslanden Medical Centre	Razvan Popescu	PI
~	~	Katja Kilcher	
~	~	Eloise Kremer	
~	~	Helen Leemann	
~	~	Eva Lehmann Fueter	
~	~	Sylvie Nuc	
~	~	Klaus Schalk	
~	~	Belinda Schegg	
~	~	Louise Seiler	
~	~	Melanie Stahel	
~	~	Michelle Suppiger	
Zurich, CH	Triemlispital	Donat Durr	PI
~	~	Maximillian Asanger	
~	~	Camillo Cetuzzi	
~	~	Irene Hones	
~	~	Eloise Kremer	
~	~	Alexandra Pfister	
~	~	Karin Scheuch	
~	~	Daniele Siciliano	
~	~	Stefan Suter	
Zurich, CH	University Hospital Zurich	Daniel Fetz	
~	~	Eloise Kremer	
~	~	Michael Muntener	
~	~	Cedric Poyet	
~	~	Frank Stenner-Liewen	
